# Growth Cone *MKK7* mRNA Targeting Regulates MAP1b-Dependent Microtubule Bundling to Control Neurite Elongation

**DOI:** 10.1371/journal.pbio.1001439

**Published:** 2012-12-04

**Authors:** Daniel Feltrin, Ludovico Fusco, Harald Witte, Francesca Moretti, Katrin Martin, Michel Letzelter, Erika Fluri, Peter Scheiffele, Olivier Pertz

**Affiliations:** 1 Institute for Biochemistry and Genetics, Department of Biomedicine, University of Basel, Basel, Switzerland; 2 Biozentrum, University of Basel, Basel, Switzerland; University of Cambridge, United Kingdom

## Abstract

Local mRNA translation in neurons has been mostly studied during axon guidance and synapse formation but not during initial neurite outgrowth. We performed a genome-wide screen for neurite-enriched mRNAs and identified an mRNA that encodes mitogen-activated protein kinase kinase 7 (MKK7), a MAP kinase kinase (MAPKK) for Jun kinase (JNK). We show that *MKK7* mRNA localizes to the growth cone where it has the potential to be translated. MKK7 is then specifically phosphorylated in the neurite shaft, where it is part of a MAP kinase signaling module consisting of dual leucine zipper kinase (DLK), MKK7, and JNK1. This triggers Map1b phosphorylation to regulate microtubule bundling leading to neurite elongation. We propose a model in which *MKK7* mRNA localization and translation in the growth cone allows for a mechanism to position JNK signaling in the neurite shaft and to specifically link it to regulation of microtubule bundling. At the same time, this uncouples activated JNK from its functions relevant to nuclear translocation and transcriptional activation.

## Abstract 2

Growth cone localization of *MKK7* mRNA switches the classic function of MKK7 protein in transcriptional control to regulation of microtubules necessary for neuronal outgrowth.

## Author Summary

MRNA transcripts are usually translated into proteins in the cytosol. Some mRNAs, however, are first transported to specific subcellular regions where they are translated to perform localized functions. This is especially important in highly polarized cells such as neurons. Here, we find that a transcript encoding the signaling molecule mitogen-activated protein kinase kinase 7 (MKK7) localizes to the growth cone of neuronal processes, where it most likely is translated. This is surprising, because MKK7 has been linked to activation of transcription in the nucleus in response to cellular insults. We find that growth cone *MKK7* mRNA transcript localization, and potentially its translation at this subcellular localization, allows the MKK7 protein to switch its function and regulate the microtubule cytoskeleton, which is necessary for neuronal outgrowth. This occurs through the formation of a spatially regulated MKK7 signaling domain in the neurite that performs this highly specialized function.

## Introduction

Local mRNA targeting and translation is an important aspect of the regulation of axonal guidance and the remodeling of synapses in the nervous system. In axons, local translation of β-*actin* mRNA has been implicated in growth cone turning [1]. Local translation of *RhoA* mRNA regulates axonal growth cone collapse [2]. Local translation of the transcription factors ELK at the synapse [3] and CREB in the axon [4] allows for retrograde signaling between these organelles and the nucleus. Local mRNA translation is also observed in response to neuronal injury. In this context, local translation of *Stat3* mRNA, and its subsequent retrograde shuttling to the nucleus induces survival signals [5]. A combination of gene expression profiling and neuronal process purification techniques, has unveiled complex local transcriptomes that depend on the neurite identity (axon/dendrite) [6]–[9], the differentiation state (young versus old axons [10]) and on neurite injury [11]. This suggests that a large variety of local translational programs regulate distinct neuronal functions depending on specific cellular states during development. However, local mRNA targeting and translation has not yet been explored during the initial process of neurite outgrowth, before axon-dendrite specification.

Jun N-terminal kinases (JNK1–3) are a subgroup of mitogen-activated kinases (MAPKs) that control transcriptional programs and the neuronal cytoskeleton [12]. While JNKs were originally considered to regulate neuronal survival or death in response to stress and injury, significant JNK activity is maintained in neurons even in the absence of stress [13]. Consistently, it recently became clear that JNKs are essential regulators of neurite growth and regeneration [14]. In this context, JNKs have been implicated in the regulation of microtubule (mt) dynamics through phosphorylation of a variety of proteins. JNKs phosphorylate and regulate mt-associated proteins (MAPs), consequently, JNK1-deficient mice display loss of axonal mt integrity [15]. JNKs phosphorylate the catastrophy promoting factors stathmins [16] and the protein SCG10 [17], which exhibits mt depolymerizing activity. JNKs also phosphorylate doublecortins to modulate neurite outgrowth and neuronal migration [18]. Finally, JNKs have also been implicated in the regulation of axonal specification [19], which might be regulated by the JNK scaffold protein (JNK interacting protein 1 [JIP1]) [20]. The three JNK isoforms are directly phosphorylated and controlled by two MAP kinase kinase (MAP2Ks), mitogen-activated protein kinase kinase 4 and 7 (MKK4 and MKK7, [21]), which are also known to be phosphorylated during neurite outgrowth of cerebellar granule neurons [13]. Further upstream, these MAP2Ks can in turn be phosphorylated by a wide variety of MAP3Ks [21],[22]. While it is clear that JNKs are essential for the control of neuronal mts, the molecular details of how the JNK signaling network is spatio-temporally wired to control distinct (and sometimes opposing) mt regulating activities versus nuclear translocation and regulation of transcriptional responses remains unknown.

Here we perform a genome-wide screen and identify 80 mRNAs that are significantly enriched in neurites of N1E-115 neuronal-like cells. We identify the neurite-enriched *MKK7* mRNA, which localizes to growth cones, where it most likely is translated, and subsequently activated in the neurite shaft through phosphorylation. We propose a model in which growth cone localization of *MKK7* mRNA, and possibly its translation at this subcellular localization triggers a spatio-temporal JNK signaling module in the neurite to specifically regulate mt bundling and neurite elongation.

## Results

### Genome-wide Screen Identifies mRNAs That Localize to the Growth Cone

**Figure 1:**
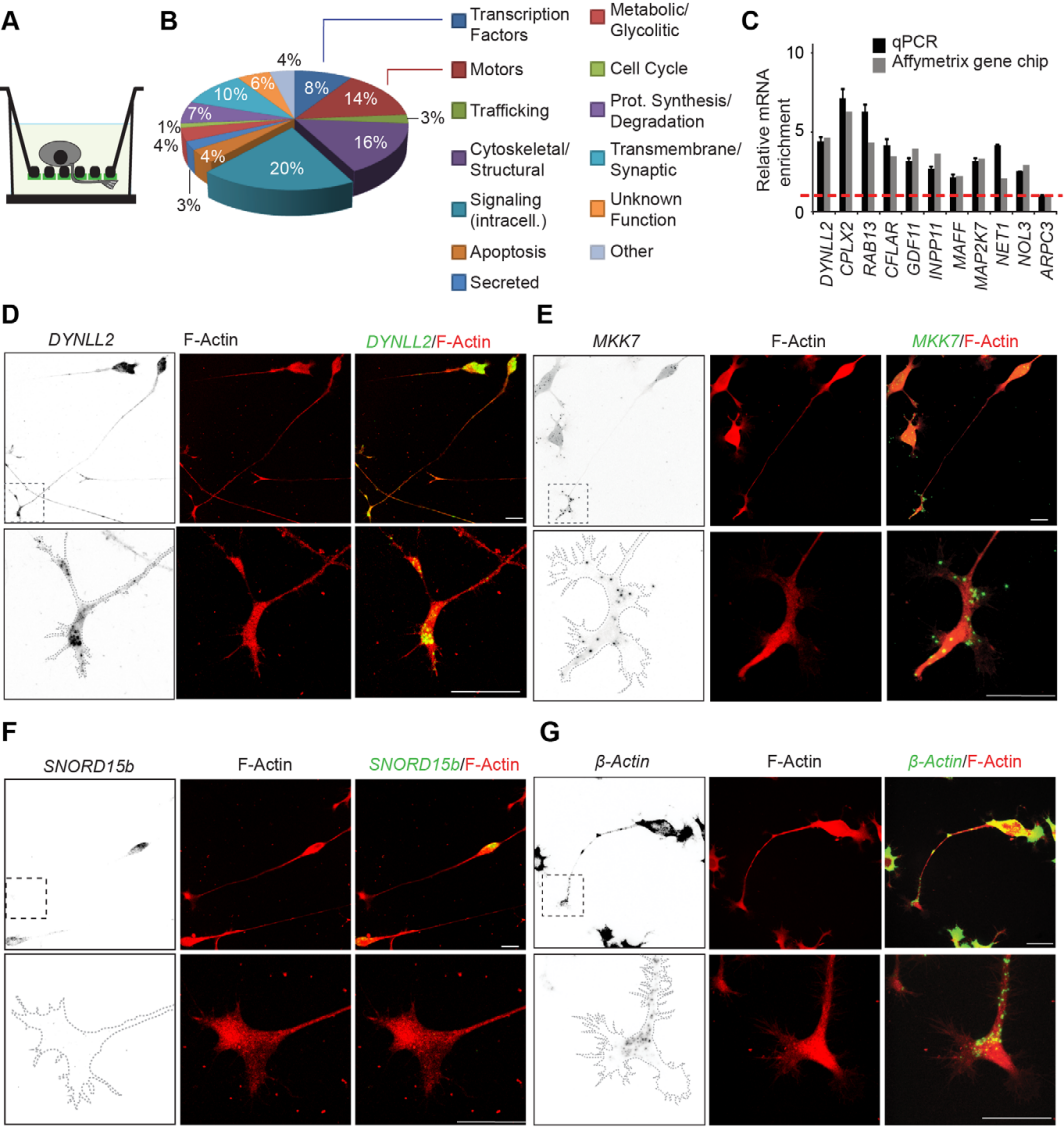
Genome-wide screen for neurite-localized mRNAs. (A) Schematics of microporous filter technology that allows for neurite purification. Asymmetric laminin coating allows to drive neurites to the filter bottom. 3-µm pore size restricts somata to the filter top. (B) Gene ontology analysis of classes of mRNAs identified. (C) Validation of mRNA neurite enrichment of selected genes. Equal amounts of purified neurite and soma mRNA fractions were subjected to RT-qPCR using specific primers for each gene. A ratio of the relative mRNA abundance in both fractions was then calculated. *n* = 2 experiments, error bars represent standard error of the mean (SEM). (D–G) Confocal fluorescence micrographs of FISH for *DYNLL2*, *MKK7*, β*-actin* mRNAs, and *SNORD15b* non-coding *RNA* in differentiated N1E-115 cells. Left panels: FISH (inverted black and white contrast [ibw]; middle panels: phalloidin-staining; right panels: overlay FISH [green]/phalloidin-staining [red]). Lower panels show magnification of the growth cone re-acquired using higher magnification. (D and E) *DYNLL2* and *MKK7* mRNAs, identified to be enriched in the neurite, are predominantly found in punctuate structures in the growth cone. (F) *SNORD15b* non-coding RNA, identified to be soma-enriched, localizes specifically to the soma. Note that the FISH probe most likely detects an unprocessed mRNA pool, explaining the cytosolic localization of the signal. (G) The bulk of β-actin mRNA is found in the soma, but a subtle pool is also present in the growth cone. Scale bars: 25 µm.

To identify on a genome-wide scale neurite-localized mRNAs during neuronal outgrowth, we used our previously described microporous filter technology [23] to fractionate neurites from the soma of N1E-115 neuronal-like cells (Figure 1A). Similar approaches have been previously used to study local mRNA translation in dendrites [24]. In this model system, differentiated N1E-115 cells are plated on a 3-µm microporous filter that has been coated with laminin on the bottom part (Figure 1A). This leads to neurite outgrowth to the bottom filter surface, allowing biochemical separation of neurites from the soma. Total RNA from purified neurite and soma equivalents was analyzed by Affymetrix gene chip technology to quantitate relative mRNA abundance. This revealed 80 mRNAs that are significantly enriched in the neurite fraction (Table S1), and encode a wide variety of different functions (Figure 1B). To validate the enrichment of mRNAs in the neurite, we used quantitative PCR coupled with reverse transcription (RT-qPCR) to compare relative mRNA abundance in both fractions and observed similar results as for the gene chip experiment (Figure 1C). We also explored the localization of a panel of these neurite-enriched mRNAs using fluorescence in situ hybridization (FISH) experiments in N1E-115 cells (Figures 1D, 1E, S1A, and S1B). While some mRNA is present in the soma, these mRNAs predominantly localized to bright punctate structures in growth cones, suggesting association with ribonucleoprotein particles (RNPs) [25]. Soma-enriched transcripts consisted mostly of small nucleolar RNAs. FISH showed that such a non-coding RNA, *Snord15b*, specifically localized to the soma and was absent from the growth cone (Figure 1F). Because β*-actin* mRNA was previously documented to be localized in neuronal growth cones [1], but was not found in our genome-wide screen, we also evaluated its subcellular localization in N1E-115 cells (Figure 1G). While β*-actin* mRNA was evident in growth cones, the bulk of the mRNA was in the soma. Thus, our purification approach, which relies on relative comparison of mRNA levels in purified neurite and soma equivalents, will only detect transcripts that are robustly enriched in the neurite, but will miss mRNAs of which only a small portion of the total pool localizes to the growth cone. The control sense FISH probes for all these transcripts exhibited very little signal (Figure S1C–S1H).

### MKK7 Activates a Spatio-Temporal JNK Signaling Module in the Neurite Shaft

**Figure 2:**
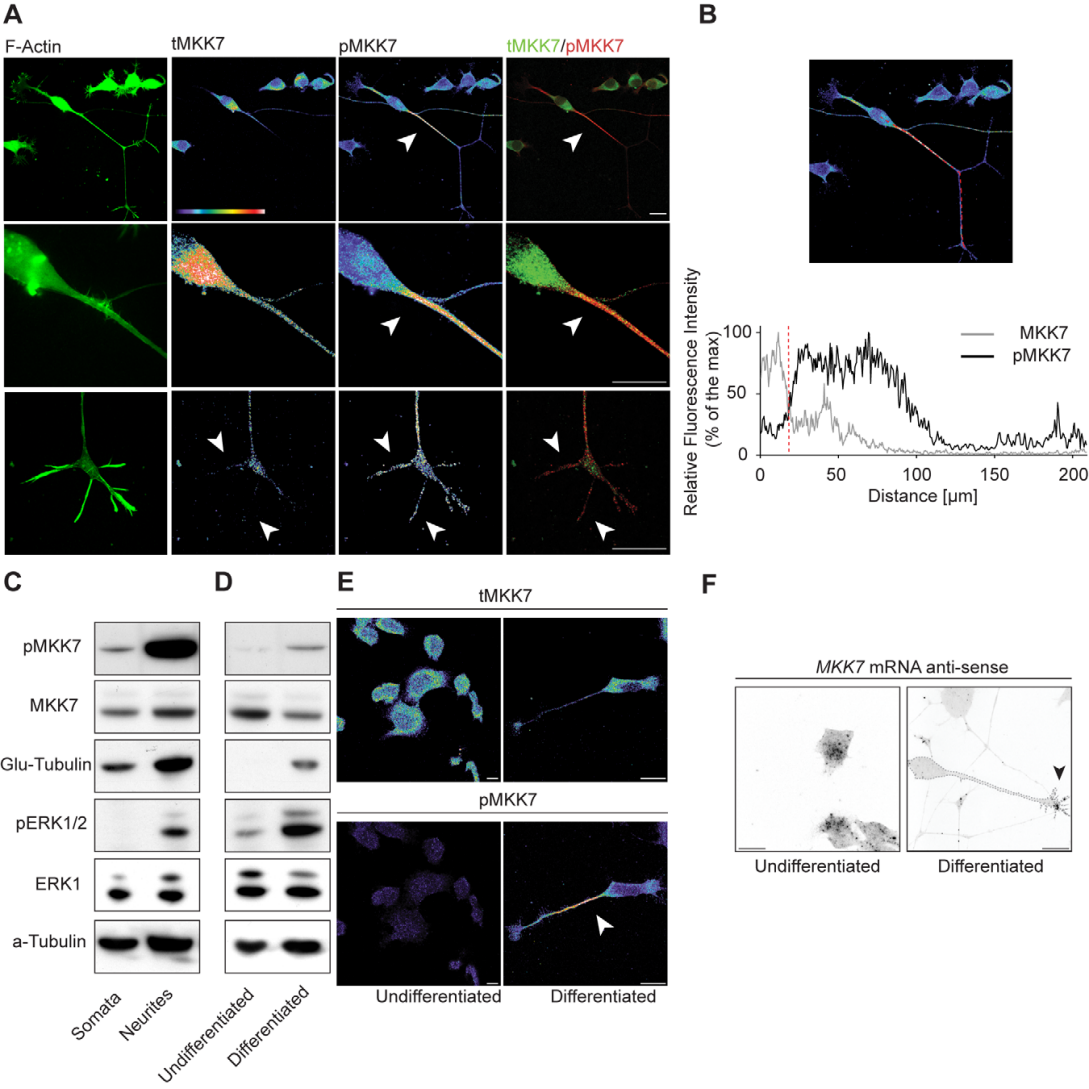
MKK7 is phosphorylated in the neurite in differentiated N1E-115 cells. (A) Representative confocal fluorescent micrographs of N1E-115 cells immunostained for tMKK7 and pMKK7. F-actin signal (phalloidin staining) is also shown. First row: global view, second row: close-up of neurite base, third row: close-up of neuronal growth cone. Images are shown in native fluorescence color, with color-coded fluorescence intensities (warm and cold colors represent high and low fluorescence intensities, respectively), or green/red color composites. Note that because confocal microscopy is used to image the subcellular location of pMKK7, the characteristic neurite to soma decreasing signal cannot be attributed to volume effects. The close-up pictures have been re-acquired at a higher zoom to provide maximal resolution. (B) Fluorescence intensity profile in tMKK7 and pMKK7 micrographs along line. Red dotted line on the graph represents neurite-soma interface. (C) MKK7 phosphorylation status in purified neurite and soma lysates. Equal amount of purified neurite and soma lysates were probed with different antibodies using Western blot analysis. Glu-tubulin and phospho-Erk1/2 (pERK1/2) serves as quality controls for neurite purification as previously described [23]. Total Erk1 (tERK1) and α-tubulin serve as loading controls. (D and E) MKK7 phosphorylation status in undifferentiated versus differentiated N1E-115 cells. (D) Global quantitation of tMKK7 and pMKK7. Equal amounts of cell lysates from N1E-115 cells in the undifferentiated or differentiated state were analyzed by Western blot. (E) Representative confocal micrographs of non-differentiated and differentiated N1E-115 cells. Cells were probed for pMKK7 and tMKK7 by immunostaining. Images are color-coded for fluorescence intensity and scaled identically. Note that elevated pMKK7 signal occurs solely in the neurite. (F) MKK7 mRNA localization in non-differentiated and differentiated N1E-115 cells. Representative confocal micrographs of *MKK7* mRNA FISH experiments are shown. Images are shown in ibw contrast. Scale bars: 25 µm.

We chose to further study the functional significance of growth cone *MKK7* mRNA localization. As a first approach, we evaluated the subcellular localization of total MKK7 protein (tMKK7) and its activated, phosphorylated form (pMKK7). In N1E-115 cells, tMKK7 displayed a cytosolic subcellular location and was excluded from the nucleus (Figure 2A, upper row). In contrast, pMKK7 was observed predominantly in the neurite shaft and exhibited a decreasing gradient from the base of the neurite to the growth cone, with a very sharp loss of pMKK7 at the base of the neurite (Figures 2A, middle row, and quantitated in 2B and S2A). This striking subcellular pMKK7 pattern was observed in 84.8%±3.1% of cells observed (*n* = 2 experiments with 50 cells counted in each experiment). A small pMKK7 pool was also significantly enriched in filopodia, but to a much lower extent than in the neurite shaft (Figure 2A, bottom row). Western blot analysis of biochemically purified neurite and soma fractions also revealed an increase of pMKK7 in the neurite, while tMKK7 was equally distributed in both fractions (Figure 2C). In contrast, the phosphorylated form of the other JNK-specific MAPKK MKK4 (pMKK4) was found to display a distinct distribution than pMKK7, being high in the soma and the growth cone and decreasing in the neurite (Figure S3A).

To further understand the role of MKK7 in neurite outgrowth, we compared pMKK7, tMKK7, and *MKK7* mRNA levels and subcellular localization in non-differentiated (e.g., cells without neurites) and differentiated N1E-115 cells. Western blot analysis revealed an increase in pMKK7 level concomitant with differentiation while tMKK7 remained constant (Figure 2D). Immunostaining revealed that this raise in pMKK7 resulted exclusively from the neurite localized pMKK7 pool (Figure 2E). In both cases, tMKK7 was excluded from the nucleus. At the transcript level, *MKK7* mRNA was found throughout the cytosol of non-differentiated cells (Figure 2F). During differentiation, 53%±3% (*n* = 23 cells) of *MKK7* mRNA RNPs relocalized to the growth cone (Figure 2F). Globally, these results show that there is an increase of pMKK7 level associated with cell differentiation and neurite outgrowth. This pMKK7 pool specifically localizes to the neurite, and might therefore regulate cytoskeletal rather than nuclear functions. At the same time, this correlates with a redistribution of *MKK7* mRNA from the cytosol to the growth cone.

**Figure 3:**
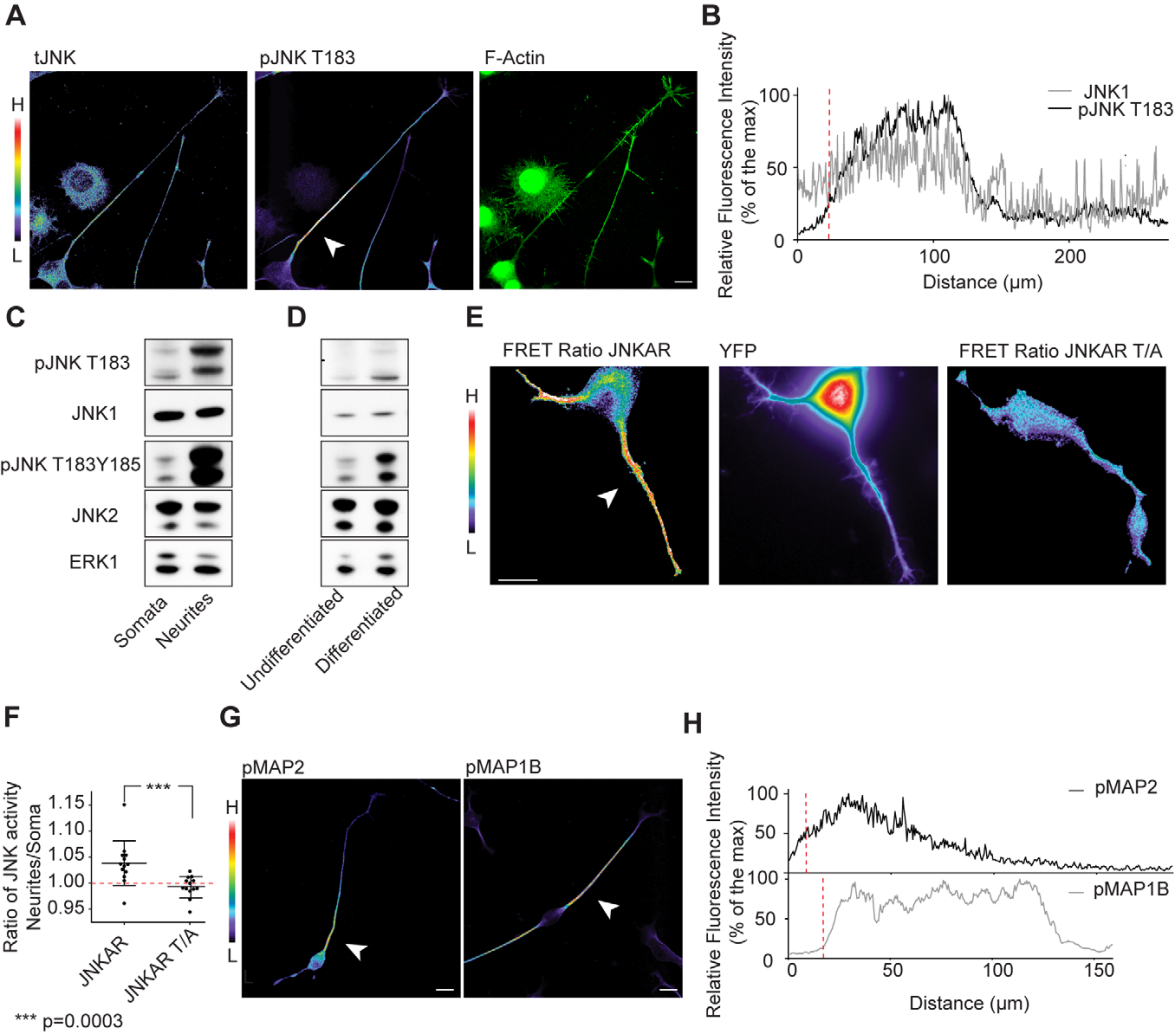
JNKs and its downstream effectors MAPs are phosphorylated in the neurite shaft. (A) Representative confocal fluorescent micrographs of differentiated N1E-115 cells immunostained for total JNK (tJNK) and pJNK T183. Images are color coded for staining intensity so that warm and cold colors represent high (H) and low (L) signal intensity. F-actin image (phalloidin staining) is represented in native fluorescence color. Arrowhead points to high pJNK T183 signal in the neurite shaft. (B) Representative fluorescence intensity profile in tJNK and pJNK T183 micrographs. (C and D) JNK T183 and T183Y185 phosphorylation status in purified neurite and soma lysates (C), and in non-differentiated versus differentiated N1E-115 cells (D). Equal amount of lysates of purified neurites and somata (C), or cells in the undifferentiated and differentiated states (D) were probed with different antibodies using Western blot analysis. (E) Analysis of JNK activity in single living cells using JNKAR FRET probe. Left panel: FRET emission ratio using JNKAR probe; middle panel: JNKAR probe distribution (YFP channel); right panel: emission ratio using JNKAR T/A non-phosphorylatable probe. FRET emission ratio pictures are color-coded so that warm and cold colors represent high and low JNK activation or probe localization. Arrowhead points to neurite-localized JNK activity. (F) Ratio of mean JNK activities in the neurite versus the soma are shown for wt or a non-phosphorylatable T/A mutant. Mean ± standard deviation (SD) is shown, *n* = 15 cells. (G) Representative confocal fluorescent micrographs of differentiated N1E-115 cells immunostained for phospho-MAP2 (pMAP2) or phosho-MAP1b (pMAP1b). Images are color-coded as mentioned elsewhere. Arrowheads point to the characteristic signal accumulation at the base of the neurite. (H) Representative fluorescence intensity profile in pMAP2 and pMAP1b micrographs. Scale bars: 25 µm.

We then evaluated the signaling events downstream of MKK7, and observed activation of JNKs in the neurite. We found that total JNK (tJNK) was evenly distributed throughout the cell and excluded from the nucleus (Figure 3A). An antibody that detects the activated, mono-phosphorylated form of JNK isoforms 1, 2, and 3 (pJNK T183) specifically stained the neurite in an identical pattern than pMKK7 (Figures 3A, 3B, and quantitated in S2B). This striking subcellular pJNK T183 pattern was observed in 82.8%±1.7% of cells observed (*n* = 2 experiments with 50 cells counted in each experiment). In contrast, an antibody that recognizes the dually phosphorylated form of JNKs (pJNK T183Y185) prominently stained the growth cone (Figures S3B and quantitated in S2C). Biochemical experiments showed that both phosphorylated JNK forms were also enriched in purified neurites (Figure 3C), and increased concomitantly with differentiation (Figure 3D). Beyond the ability of these JNK isoforms of being phosphorylated in the neurite, we also directly measured JNK activity in single living cells using a fluorescence resonance energy transfer (FRET)-based reporter for JNK activation (JNKAR) [26]. We observed JNK activity throughout the neurite, including the growth cone, but not in the soma (Figure 3E). Time-lapse analysis showed that this neurite-localized pool of JNK activation was stable for timescales of minutes (Video S1). A non-phosphorylatable JNKAR T/A mutant probe did not exhibit increased FRET signal in the neurite (Figure 3E and 3F). The observation that the FRET activation pattern does not precisely recapitulate the striking pMKK7 and pJNK T183 patterns can be explained because JNKAR is a cytosolic probe that will rapidly diffuse upon phosphorylation, and because JNKAR can be phosphorylated by all JNK isoforms. These results formally show the existence of localized JNK activity in neurites.

We then evaluated the subcellular localization of multiple mt-regulating JNK substrates in their phosphorylated form. Immunostaining for the phosphorylated forms of MAP1b and MAP2 recapitulated the subcellular distributions of pMKK7 and pJNK T183 (Figures 3G, 3H, and quantitated for pMAP1b in S2D). In contrast, phospho-stathmin and JNK interacting proteins (JIP1 and JIP3, scaffolds for JNK signaling) localized to growth cones and recapitulated the subcellular location of pMKK4 and pJNK T183Y185 (Figure S3C–S3E). Phospho-doublecortin was homogeneously distributed in the neurite and did not display the characteristic gradient exhibited by pMKK7, pJNK T183. and pMAP1b and (Figures S3F and quantitated in S2E). This suggests the existence of distinct spatio-temporal JNK signaling modules in the neurite. Altogether, these results suggest a link between growth cone localized *MKK7* mRNA and activation of MKK7 protein and downstream effector pathways in the neurite.

### MKK7 Controls Neurite Elongation by Regulating mt Bundling

**Figure 4:**
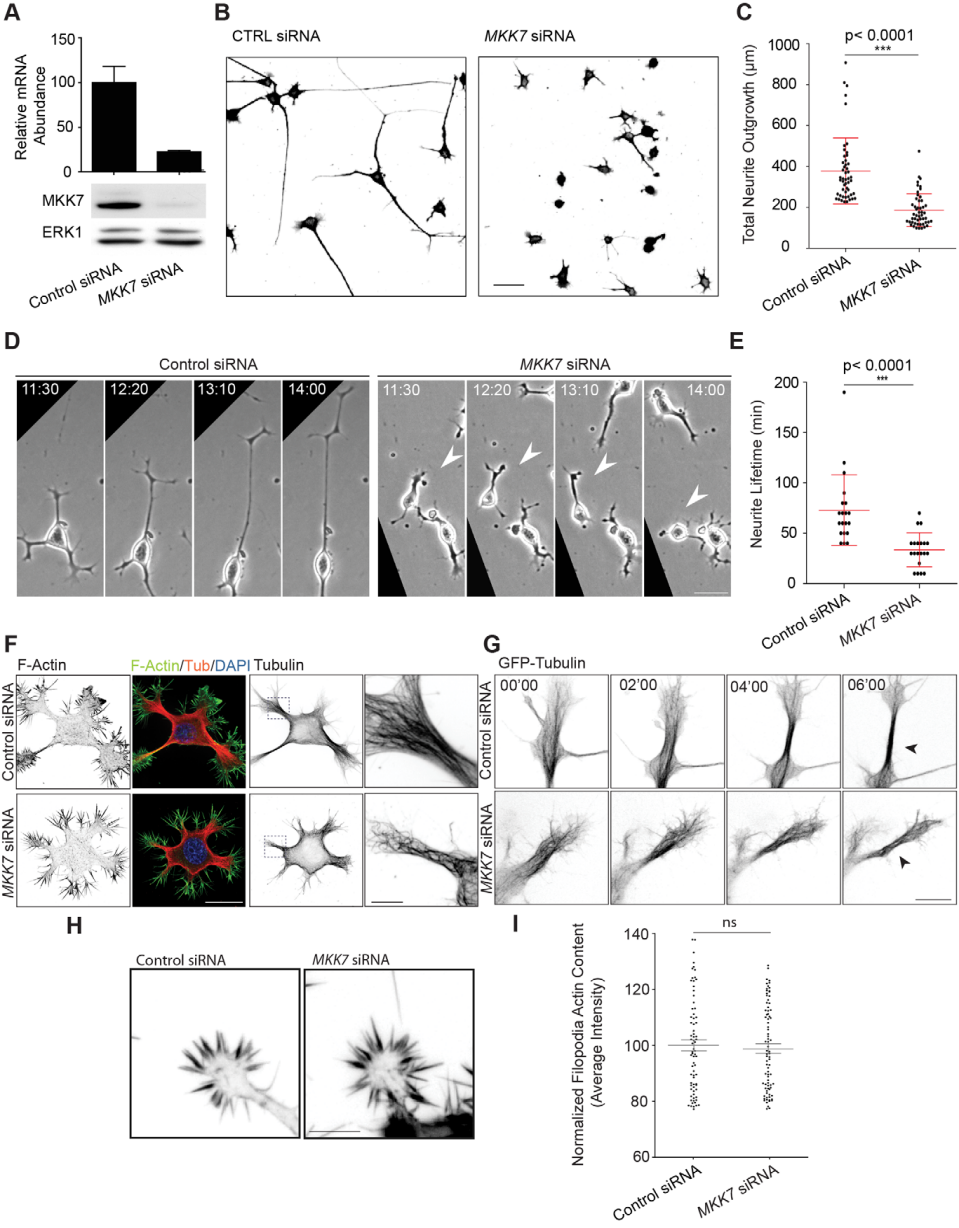
MKK7 controls neurite elongation through regulation of mt bundling in the neurite shaft. (A) *MKK7* KN efficiency. Equal amount of RNA or protein cell lysates of control or MKK7 siRNA-transfected cells were assessed by RT-qPCR (*n* = three experiments) or Western blot. (B) Representative micrographs of α-tubulin immunostained control of MKK7 siRNA-transfected cells. SiRNA-transfected N1E-115 cells were differentiated through serum starvation and replated on laminin-coated coverslips. ibw contrast is shown. Scale bars: 50 µm. (C) Total neurite outgrowth/cell measurements of (B). Measurements of the 10% of the cells with longest neurites are shown. Error bars represent SD, *n* = 500 cells. (D) Neurite outgrowth dynamics of control or MKK7 siRNA transfected cells. Differentiated, siRNA-transfected N1E-115 cells were replated on laminin-coated coverslips and imaged using phase-contrast time-lapse microscopy. Arrowheads point to neurite retraction events. Scale bars: 40 µm. (E) Quantification of neurite extension lifetime. Neurite extension lifetime was manually measured on multiple time lapses. Error bars represent SD, *n* = 20 cells. (F) Confocal fluorescent micrographs of differentiated control and *MKK7* KN N1E-115 cells mmunostained for α-tubulin, phalloidin, and DAPI. Composite images or ibw contrast are shown. Close-ups (re-acquired at higher magnification) show the state of the mt cytoskeleton at high resolution. Scale bars: 25 µm, 12 µm (close-up). (G) Time-lapse confocal video microscopy of control and *MKK7* KN N1E-115 cells expressing GFP-tubulin. Note the parallel bundling of mts in control cells versus curly, bent mts that are unable to coalesce in a bundle in the *MKK7* KN cells (shown by arrowheads). Scale bar: 12 µm. (H) F-actin micrographs of growth cones of control and *MKK7* KN N1E-115 cells. Cells were stained with phalloidin and imaged with identical conditions using an epifluorescence microscope. Scale bar: 5 µm. (I) Normalized mean fluorescence intensity of F-actin signals in single filopodia. *n* = 50 filopodia from five growth cones.

To study the function of MKK7 during neurite outgrowth, we used RNA interference to knockdown (KN) *MKK7* mRNA (Figure 4A). This led to a potent reduction in neurite outgrowth (Figure 4B and 4C). To explore if this is a global cell differentiation or a specific cytoskeletal defect, we examined the cell morphology of control and *MKK7* KN cells. In the undifferentiated state, N1E-115 cells display flat lammelipodial protrusions that are not sensitive to *MKK7* KN (Figure S4A). During differentiation N1E-115 cells lift up, adopt a round and loosely adherent soma, and extend neurites. Upon differentiation, *MKK7* KN cells still rounded up (Figure S4A, and quantitated in S4B), but were only able to initiate short neurites that were still capped by a growth cone (Figure S4A). Evaluating the dynamics of the neurite outgrowth process using phase contrast time-lapse microscopy revealed that neurites of *MKK7* KN cells were highly unstable and frequently retracted precluding the formation of long neurites (Figure 4D and 4E; Video S2). These results show that MKK7 is an essential regulator of neurite elongation.

We then explored which cytoskeletal defects could be the cause of this effect. Because, the signaling events downstream of MKK7 are highly likely to involve mts [15],[17],[27], we evaluated the mt cytoskeleton in fixed, immunostained, as well as live cells expressing green fluorescent protein (GFP)-tubulin. Newly formed neurites in control cells displayed highly parallel, mt bundles. In contrast, neurites from *MKK7* KN cells displayed curly and bent mts that were unable to coalesce in obvious bundles (Figure 4F and 4G; Video S3). *MKK7* KN cells were able to recruit mts to the growth cone (Figures S4C, quantitated in S4D) and did not display any defects in centrosome formation (Figure S4E and S4F, observed in all of 20 cells/condition). To exclude the possibility that frequent neurite collapse is the result of a defect in actin dynamics, we examined the F-actin cytoskeleton in growth cones. In the morphodynamic state of extension, no obvious defect was observed when F-actin density was quantitated in growth cone filopodia (Figure 4H and 4I). This was also apparent when growth cone F-actin dynamics were studied using the Lifeact-GFP probe (Video S4) [28].

We conclude that MKK7 specifically regulates mt bundling in the neurite, most likely to provide the rigidity necessary for outgrowth above a critical length. This is consistent with the idea that the JNK substrates Map1B and MAP2 [15], which are key regulators of mt bundling, are phosphorylated in the neurite [29].

### Identification of Determinants That Allow Growth Cone *MKK7* mRNA Targeting

To explore the functional significance of growth cone *MKK7* mRNA localization, we identified the determinants, often localized in 3′-UTRs [7], which allow mRNA targeting to the growth cone. Mapping of the *MKK7* locus had previously revealed that alternative splicing generates six protein-coding mRNA isoforms, leading to proteins with three different N-termini and two different C-termini [30]. However, in the latter study the 3′-UTR sequences were not taken in account. The ENSEMBL database indicates the existence of two alternative 3′-UTRs, a 138-nucleotide-long 3′-UTR1, and a 2,067-nucleotide-long 3′-UTR2. The latter 3′-UTR2 sequence is generated by retention of the intron situated between exons 13 and 14, leading to insertion of a STOP codon at the end of exon 13 (Figure S5A). As a consequence, 3-UTR2 containing mRNAs encode MKK7 isoforms with truncated C-termini. Furthermore, this also implies that the 3′-UTR1 sequence is contained within the 3′-UTR2 (Figure S5A). Each of the six MKK7 isoforms contains the kinase domain, and except for the report that two specific MKK7 isoforms display different kinase activity in vitro [30], their functional relevance remains unknown [21]. In this work, we collectively refer to the six possible MKK7 protein isoforms as MKK7. Rapid amplification of cDNA ends (3′-RACE) showed that 3′-UTR1 containing mRNAs were expressed in non-differentiated and differentiated cells (unpublished data). However, no specific probe can be produced to detect these 3′-UTR1 containing mRNA species, and it is not possible to measure their abundance in different differentiation states. Higher levels of 3′-UTR2 containing mRNAs were observed during differentiation using quantitative RT-qPCR (Figure S5B).

**Figure 5:**
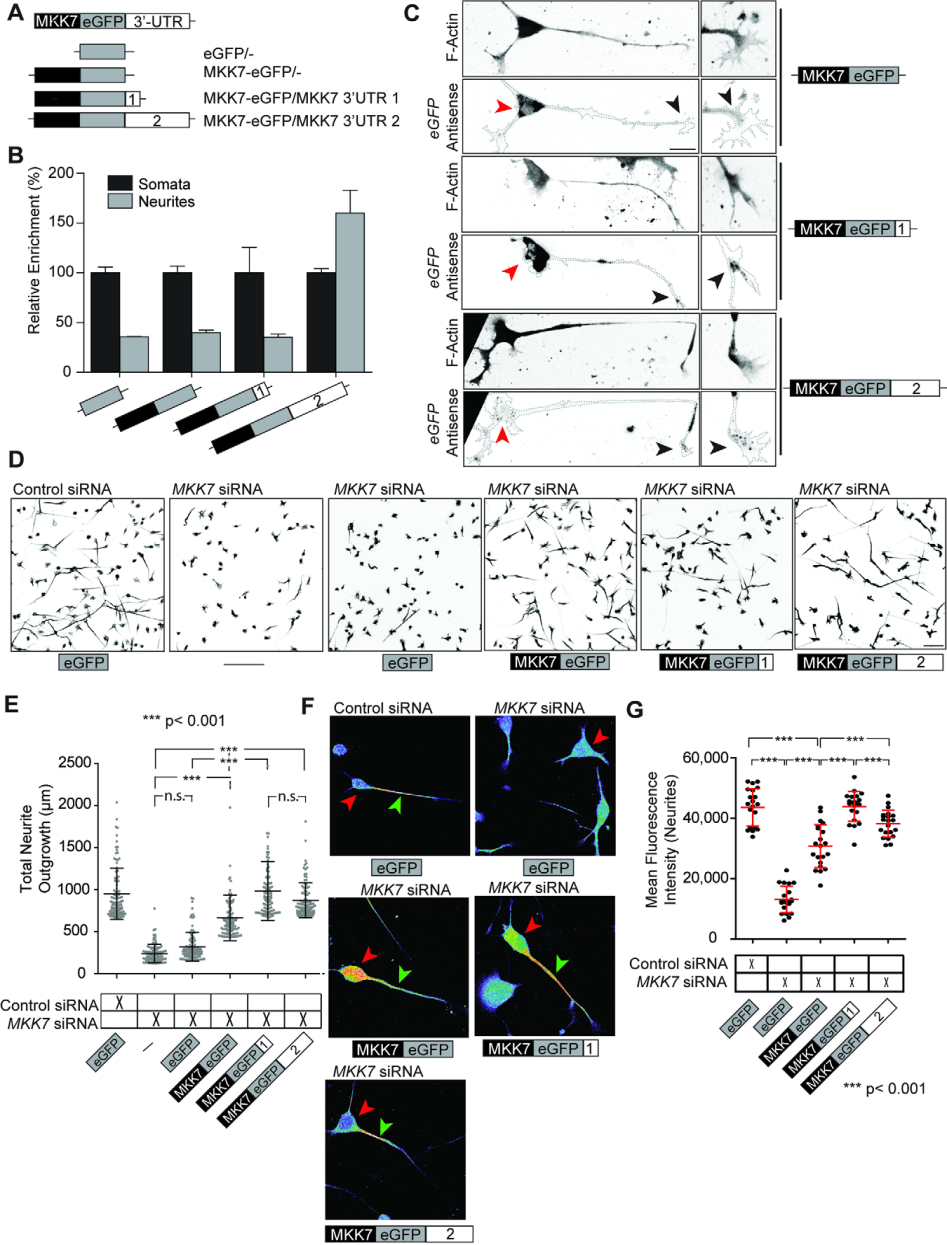
Identification of determinants that allow *MKK7* mRNA localization and functional assessment of *MKK7* mRNA localization. (A) Schematic of MKK7-GFP expression constructs flanked with different 3′-UTR sequences. (B) Relative neurite enrichment of exogenously expressed mRNAs determined as performed in Figure S5D. *n* = two experiments. (C) Subcellular localization of exogenously expressed *MKK7-GFP* mRNAs. Representative FISH micrographs of exogenously expressed *MKK7-GFP/-, MKK7-GFP/3′-UTR1*, and *MKK7-GFP/3′-UTR2* mRNAs using a GFP antisense probe. F-actin signal (phalloidin staining) is also shown. Images are in ibw contrast. Black arrows point to growth cones, red arrows point to somata. Note different mRNA localizations: MKK7-GFP/-, soma; MKK7-GFP/3′-UTR1, soma and growth cone; MKK7-GFP/3′-UTR2, growth cone. Scale bar: 25 µm. (D) Representative micrographs of differentiated N1E-115 *MKK7* KN cells rescued with different exogenously expressed *MKK7-GFP* mRNAs. Cells were immunostained for α-tubulin and are shown in ibw contrast. Note that with siRNA and plasmid transfection efficiencies of 100% and 80%, respectively (unpublished data), average whole population measurements can be made. Scale bar: 100 µm. (E) Neurite outgrowth measurements from micrographs in (D) are shown. Measurements from 10% cells with longest neurites are shown, *n* = 1,000 cells. (F) Representative micrographs of pMKK7 signals of differentiated N1E-115 *MKK7* KN cells rescued with different exogenously expressed MKK7-GFP constructs. Images are color-coded so that warm and cold colors represent high and low pMKK7 signal. Coverslips were stained, imaged, and fluorescence intensities scaled with identical conditions within one experiment. Images were acquired with a confocal microscope with a maximally open pinhole for adequate signal quantification. Red arrowheads point to the soma, green arrowheads point to the neurite. Scale bar: 25 µm. (G) Quantification of pMKK7 signals in the neurite. Mean neurite fluorescence intensities per neurite are shown. Note robust neurite pMKK7 signal recovery with MKK7-GFP/3-UTR1 and 3′UTR2 constructs. Error bars represent SD, *n* = 20 cells.

To evaluate the ability of these 3′-UTR sequences lo localize mRNA transcripts, we flanked these two sequences at the 3′ of a chimeric human β-globin construct that was previously used to study mRNA transport to fibroblast lamellipodia (Figure S5C) [31]. We then exogenously expressed these constructs in N1E-115 cells, biochemically purified neurite and soma fractions, and used RT-qPCR to determine the relative enrichment of the exogenously expressed mRNAs in the neurite and the soma. We found that the 3′-UTR2 allowed enrichment of the β*-globin* mRNA in the neurite, while this was not observed with the 3′-UTR1 (Figure S5D). To study the function of these two distinct 3′-UTR sequences, we engineered siRNA-resistant, GFP-tagged human MKK7 constructs that either remained unflanked, or were flanked with 3′-UTR1 or 3′-UTR2 sequences at their 3′ (Figure 5A). We first validated the relative enrichment of the mRNAs encoded by these exogenously expressed constructs using the neurite purification assay, and observed identical results as with the β-globin constructs (Figure 5B). Evaluating the subcellular localization of these chimeric mRNAs with FISH using an antisense GFP probe, yielded an additional level of complexity (Figures 5C and S6, soma and growth cone mRNAs highlighted by black and red arrowheads, respectively). Exogenously expressed *MKK7-GFP/-* mRNA solely localized to the soma. While the bulk of *MKK7-GFP/3′-UTR1* mRNA also localized to the cell soma, a small mRNA pool was also evident in the growth cone. By contrast, the *MKK7-GFP/3′-UTR2* mRNA robustly localized to the growth cone and was only marginally observed in the soma. This is consistent with our data obtained with a FISH probe recognizing endogenous transcripts containing the 3′-UTR2 sequence (Figure 1E). The contradicting results between our enrichment (using the neurite purification assay) and our localization measurements (using FISH) concerning the 3′-UTR sequence, can be explained because the *MKK7-GFP/3′-UTR1* mRNA is present both in the soma and the growth cone. Here, the large somatic mRNA pool obscures the subtle growth cone pool in neurite purification experiments in which neurite and soma mRNA equivalents are compared.

These results show that both the 3′-UTR1 and 3′-UTR2 sequences allow for growth cone *MKK7* mRNA localization. However, in the case of the 3′-UTR1 a large mRNA pool also resides in the soma.

### Growth Cone *MKK7* mRNA Localization Is Essential for Neurite Elongation, Robust mt Bundling, and Selective MKK7 and JNK T183 Phosphorylation in the Neurite

We then evaluated the ability of these constructs, that allow differential *MKK7* mRNA localization, to rescue MKK7 function in *MKK7* KN cells (Figure 5D and 5E). While the *MKK7-GFP/-* mRNA was able to rescue neurite outgrowth to some extent, it could not restore the level of growth observed in control cells. However, the *MKK7-GFP/3′-UTR1* and *3′-UTR2* mRNAs fully rescued neurite outgrowth, demonstrating an important role for growth cone *MKK7* mRNA localization in this process. This was also apparent when neurite outgrowth dynamics were evaluated. Here, rescue with *MKK7-GFP/-* led to less stable neurite outgrowth dynamics than with *MKK7-GFP/3′-UTR1* or *3′-UTR2* mRNAs (Videos S5 and S6). Furthermore, neurite outgrowth robustness correlated with the ability of the different constructs to rescue mt bundling (Figure S7A and S7B). Overexpression of *MKK7-GFP/3′-UTR1* or *3′-UTR2*, but not *MKK7-GFP/-* mRNAs, led to longer neurites (Figure S8A). Western blot analysis (Figure S8B), and assessment of MKK7 fluorescence intensities (Figure S8C), revealed that all three MKK7-GFP mRNAs led to similar MKK7 protein expression levels.

To understand the relative contributions of the catalytic activity of MKK7, and of the ability of *MKK7* mRNA to localize to the growth cone, we also tested previously described constitutively active (CA) S271D T275E and inactive (CI) S271A T275A MKK7 mutants in the MKK7 KN rescue experiment [32]. We observed that the CA MKK7 still necessitated 3′-UTR1 or 3′-UTR2 sequences to rescue robust neurite outgrowth (Figure S9A and S9B), suggesting that *MKK7* mRNA localization provides an additional layer of regulation on top of basic MKK7 activation. CI MKK7 was not able to rescue neurite outgrowth irrespectively of the presence or absence of any 3′-UTR (Figure S9A and S9B). This indicates that both MKK7 catalytic activity, and *MKK7* mRNA 3′-UTR sequences are required for proper neurite elongation, and excludes the possibility of independent function of the 3′-UTRs.

To functionally link these phenotypes with MKK7 signaling, we evaluated subcellular tMKK7 and pMKK7 patterns in this rescue experiment. At the total protein level, MKK7 exogenously expressed from the different mRNAs all displayed identical subcellular localization patterns (Figure S8D). High protein levels were found in the soma (but were excluded from the nucleus), and were decreasing in the neurite towards the growth cone. Different pMKK7 subcellular patterns were however observed in response to rescue with the different constructs (Figures 5F, quantification of fluorescence profiles in S10A, quantification of fluorescence intensities in 5G and S10B). While control cells displayed the characteristic pMKK7 pattern that is focused at the neurite base, and absent from the cell soma, MKK7 protein originating from soma-localized *MKK7-GFP/-* mRNA was mainly phosphorylated in the soma and exhibited only diffuse signal in the neurite. MKK7 protein originating from somatic and growth cone-localized *MKK7-GFP/3′-UTR1* mRNA was robustly phosphorylated at the neurite base and still to some extent in the soma. Finally, as observed in control cells, MKK7 originating from the solely growth cone-localized *MKK7-GFP/3′-UTR2* mRNA was exclusively phosphorylated at the neurite base as in control cells. These results show a parallel between the specific subcellular localizations of *MKK7* mRNAs and MKK7 protein phosphorylation, which in turn correlate with the robustness of neurite elongation. Similar results were observed when the subcellular localization of the downstream target pJNK T183 phosphosite was evaluated (Figure S11).

Altogether, these results show that *MKK7* mRNA growth cone localization is necessary for specifying neurite-localized pMKK7 and downstream pJNK T183 signaling domains, which in turn modulate mt bundling and neurite elongation.

### *MKK7* mRNA 3′-UTR Sequences Lead to Local Translation within Neuronal Growth Cones

**Figure 6:**
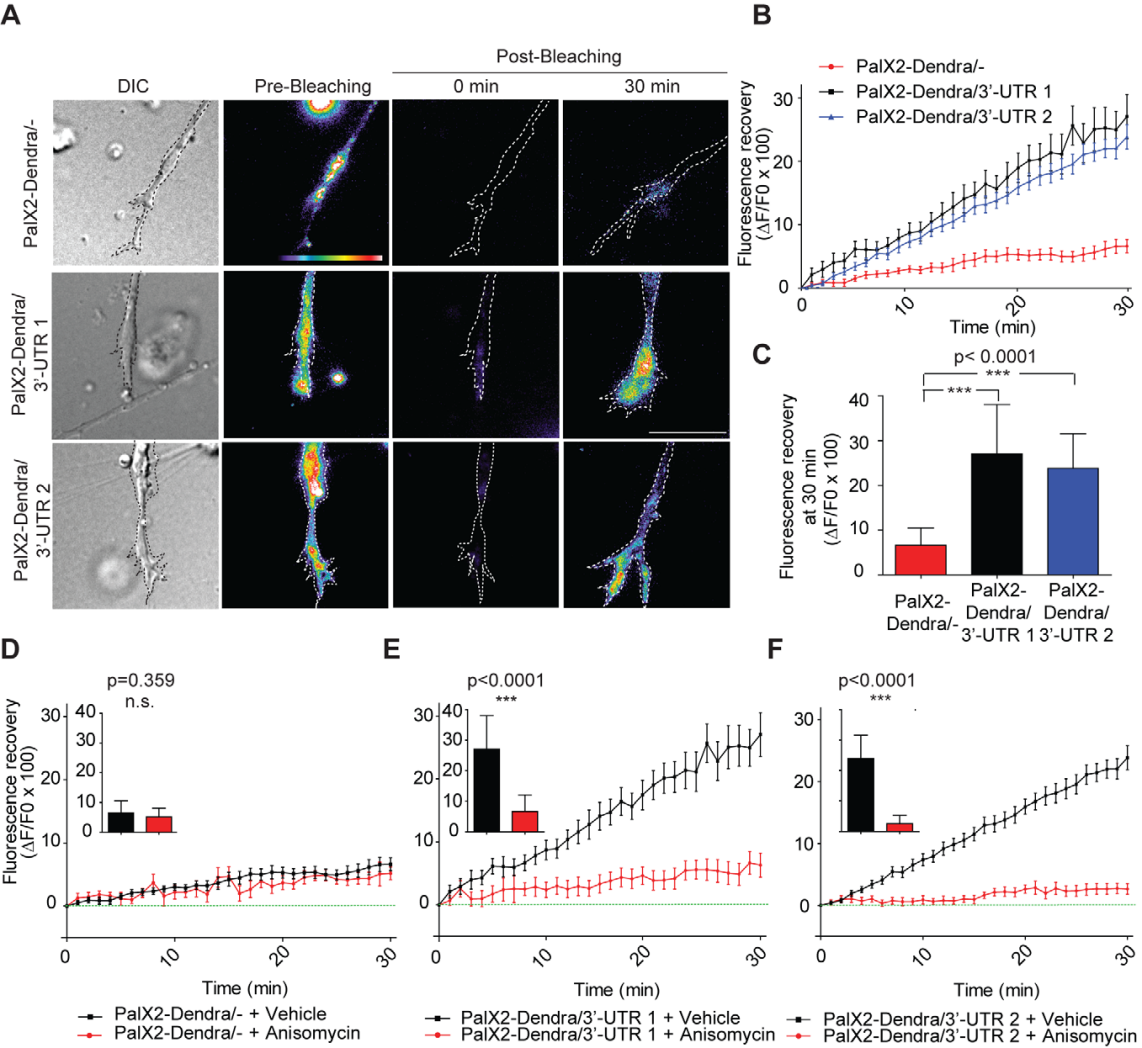
*MKK7* mRNA 3′-UTR sequences lead to growth cone mRNA translation. (A) Representative micrographs of PalX2-Dendra2/-, 3′-UTR1, 3′-UTR2 reporters in live growth cones in the pre- and post-bleaching state at different time points (0 and 30 min). N1E-115 cells were transiently transfected with the PalX2-Dendra2 reporters, differentiated and replated on laminin-coated coverslips for 24 h. One image was acquired in the pre-bleaching state, the growth cone and 50 µm of the neurite were bleached using intense 488 nm laser light, and green fluorescence recovery kinetics were then acquired using time-lapse microscopy. Images are color-coded so that warm and cold colors represent high and low green fluorescence intensity. A differential interference contrast (DIC) image is also shown. Note that the pre-bleaching image is scaled differently from the post-bleaching images because it displays higher fluorescence intensities. Results from two independent experiments were merged. Scale bar: 10 µm. (B) Fluorescence recovery kinetics in growth cones. The ratio of the increase in mean growth cone fluorescence intensity between two consecutive frames (ΔF) over the initial mean growth cone fluorescence (F_0_) immediately post-bleaching times 100 is shown. Mean ± SEM is shown. PalX2-Dendra2/- (*n* = 14 cells); PalX2-Dendra/3′-UTR1 (*n* = 11 cells); PalX2-Dendra/3′-UTR2 (*n* = 16 cells). (C) Mean fluorescence recovery 30 min post-bleaching of the different fluorescent reporters. Error bars represent SD. *n* as in (B). (D–F) Fluorescence recovery kinetics, and mean fluorescence recovery at 30 min of the three reporters in presence and absence of anisomycin. Cells were treated with 40 µM anisomycin 20 min before Dendra2 photobleaching. Error bars represent SEM and SD in the line graphs and in the bar graphs, respectively. (D) Pal2X-Dendra2/- (+vehicle *n* = 14 cells, +anisomycin *n* = 9 cells), (E) Pal2X-Dendra2/3′-UTR1 (+vehicle *n* = 10 cells, +anisomycin *n* = 10 cells), (F) Pal2X-Dendra2/3′-UTR2 (+vehicle *n* = 16 cells, +anisomycin *n* = 11 cells).

To address if *MKK7* mRNA 3′-UTR sequences allow not only localization but also translation in the growth cone, we took advantage of a recently described sensor for local translation, which consists of a membrane-targeted, photoconvertible Dendra2 fluorophore, PalX2-Dendra2 [33]. This sensor was flanked at its 3′ with *MKK7* mRNA 3′-UTR1 or 3′-UTR2 sequences and exogenously expressed in N1E-115 cells. Rather than using photoconversion [33], which in our hands led to poor elimination of the green fluorophore form, we illuminated the growth cone and the first 50 µm of the neurite shaft with intense green light to bleach the Dendra2 fluorophore. Because membrane targeted proteins diffuse at a rate of ∼50 µm/h [34], any newly green fluorescence appearing in the growth cone in a period of less than 1 h should derive from locally translated protein. We observed that PalX2-Dendra2 was exclusively present at the plasma membrane, and absent from vesicular structures, excluding the possibility that trafficking might be the source of any growth cone fluorescence recovery (Figure S12A; Video S7). The PalX2-Dendra2 reporters were also expressed at identical levels allowing for a fair comparison between the different constructs (Figure S12B). We found that PalX2-Dendra2 not flanked with any 3′-UTR sequence poorly recovered fluorescence post bleaching, whereas 3′-UTR1 or 2 flanked reporters displayed robust green fluorescence growth cone recovery (Figure 6A–6C; Video S8). The amplitude and kinetics of fluorescence recovery were comparable to those observed for local translation of RanBP1 mRNA in response to neuronal injury [35]. Furthermore, this fluorescence recovery was sensitive to the translation inhibitor anisomycin (Figure 6D–6F).

These results formally show that the 3′-UTR1 and 3′-UTR2 sequences in the *MKK7* mRNA leads to growth cone translation.

### Functional Characterization of a Neurite-Localized JNK Signaling Module

**Figure 7:**
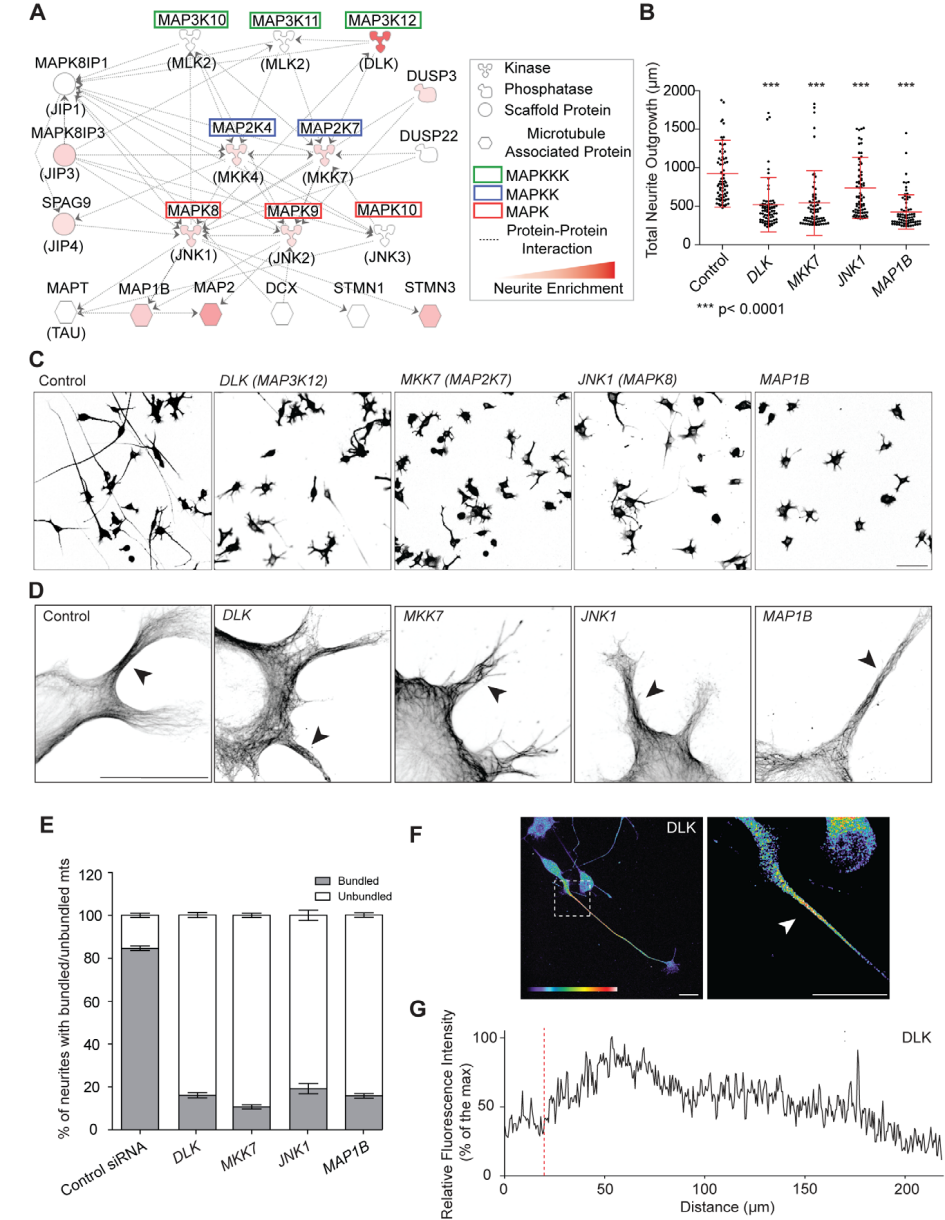
Characterization of a neurite-localized JNK signaling network. (A) JNK signaling network identified in the neurite proteome. Hugo gene (top) and common protein names (bottom, in brackets) are shown. Signaling network was built using Ingenuity Pathway software package, taking into consideration proteins from our proteomics screen that are either significantly enriched in the neurite (quantitated by red color code for the degree of enrichment) or at least present in the neurite and the soma (color-coded in white). Soma-localized proteins were ignored. Each line represents a documented direct protein-protein interaction. (B) Total neurite outgrowth length measurements in response to KN of gene products from the JNK network that phenocopy the *MKK7* KN phenotype. Measurements of the 10% cells with longest neurites are shown. Mean ± SD is shown, *n* = 700 cells for each siRNA. Full siRNA screen dataset is shown in Figure S13. (C) Representative micrographs of α-tubulin immunostained control of siRNA-transfected cells in ibw contrast. Scale bar: 100 µm. (D) Loss of mt bundling capability induced by siRNA-mediated loss of functions of these proteins. High resolution confocal micrographs of siRNA-transfected, differentiated N1E-115 cells stained for α-tubulin are shown. Arrowheads point to neurites with the representative phenotypes. Scale bar: 25 µm. (E) Quantification of occurrence of mt bundling phenotypes observed in (D). Percentage of neurites with bundled/unbundled mts are shown. SEM from two sets of quantifications of 30 cells are shown. Approximately, four neurites/cell were evaluated. (F) Representative confocal fluorescent micrographs of differentiated N1E-115 cells immunostained for DLK. Image is color coded for staining intensity so that warm and cold colors represent high and low signal intensity. Scale bar: 25 µm. (G) Representative fluorescence intensity profile of DLK localization as shown in (F). Red line represents neurite/soma interface.

MKK7 is likely to interact with a large signaling network including multiple MAPKKKs, MAPKKs, MAPKs, phosphatases, scaffold proteins, and mt-regulating JNK substrates. To pinpoint the specific JNK signaling module that controls mt bundling and neurite elongation, we mined our previously published neurite and soma N1E-115 proteome dataset [23] for neurite-localized JNK interacting proteins (Figure 7A; Table S2). We then performed an siRNA screen targeting these gene products and evaluated neurite length and dynamics. The screen was performed in triplicate and some variation was observed (Figure S13A–S13C). KN efficiency was assessed when antibodies were available (Figure S13D). We found that KN of the MAPKKK dual leucine zipper kinase (*DLK*, *MAP3K12*), *JNK1* (*MAPK8*), and MAP1b (*MAP1B*) always recapitulated the *MKK7* KN phenotype in terms of neurite length (Figure 7B and 7C), instable neurite outgrowth (Video S9), and loss of mt-bundling in the neurite (Figure 7D and 7E). KN of the other gene products did not lead to any obvious phenotype. Indeed, we cannot rule out that in some cases, we might miss some phenotypes because of low KN efficiency or penetrance. We also observed that the MAPKKK DLK displayed similar subcellular localization at the protein level as pMKK7, pJNK T183, and pMAP1b (Figures 7F, 7G, and S2F), further suggesting that DLK, MKK7, JNK1, and MAP1b are part of a JNK signaling network controlling neurite elongation.

To formally address the possibility that these components are organized in a linear signaling module, we systematically knocked down all signaling network components one by one using siRNA and used immunostaining to evaluate pMKK7, pJNK T183, and pMAP1b status, comparing short neurites from control and KN cells. As controls, we also evaluated pMKK4 status, and performed identical experiments in *JNK2* KN cells. *DLK* and *MKK7* KN led to loss of pMKK7, pJNK T183, and pMAP1b signals in neurites (Figures S14 and S15). Consistently with the hierarchy of the proposed signaling network, *JNK1* KN did not affect neurite-localized pMKK7, but led to loss of pJNK1 and pMAP1b signals (Figure S16). *JNK2* KN did not affect phosphorylation of any of the signaling network components (Figure S17), neither were pMKK4 signals affected in response to KN of the different components (Figures S14, S15, S16, S17). Globally, these results formally show that DLK, MKK7, and JNK1 are a part of a linear JNK signaling network dedicated to MAP1b phosphorylation in the neurite, allowing for mt bundling and efficient neurite elongation.

### JNK Signaling Network in Hippocampal Neurons

**Figure 8:**
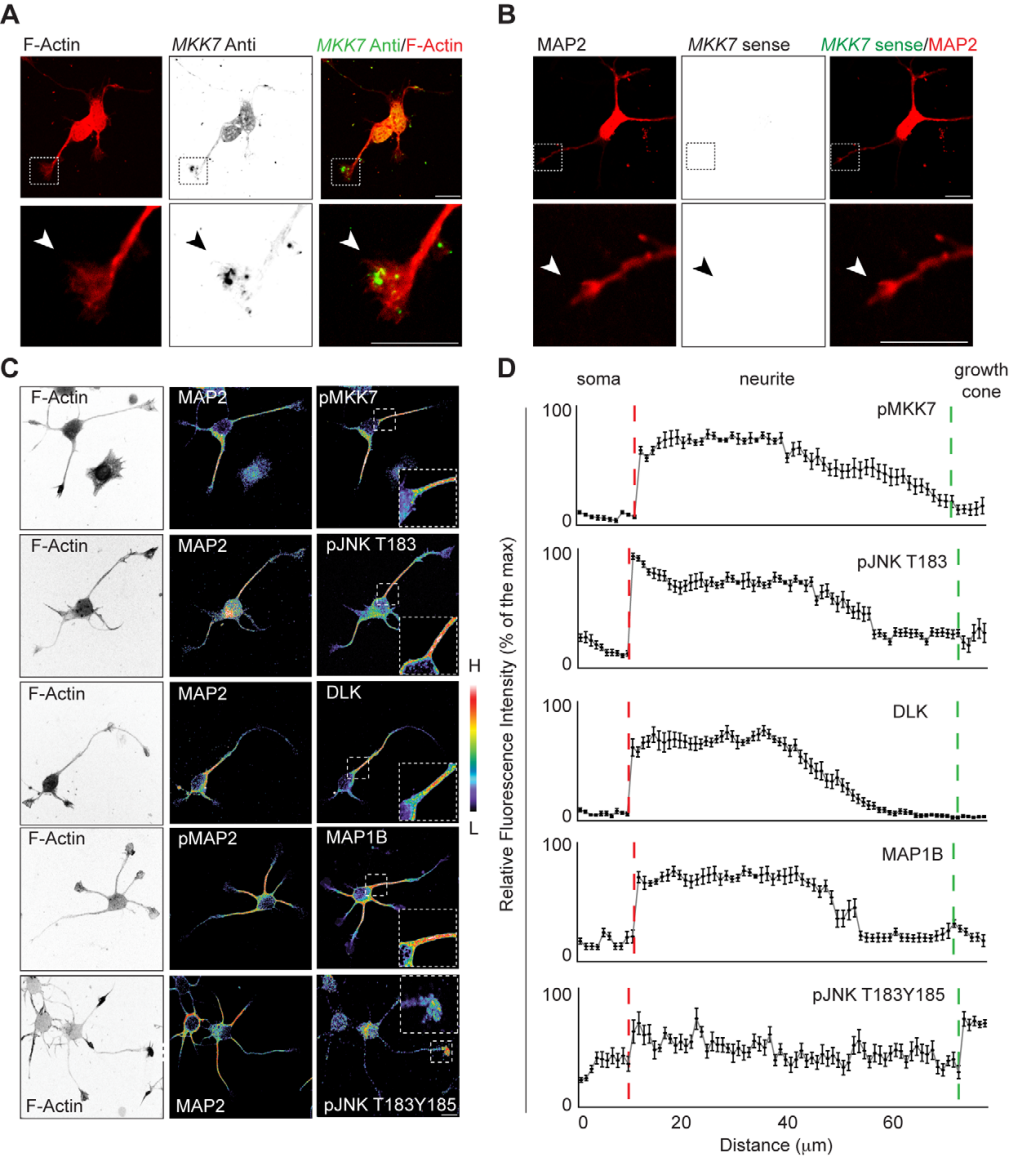
Validation of neurite-localized JNK network in hippocampal neurons. E18 hippocampal neurons were plated on poly-D-lysine–coated coverslips. Cells were fixed at DIV 1. (A) Confocal fluorescence micrographs of *MKK7* mRNA FISH using antisense probe. Top panels: fluorescence signal in ibw contrast. Bottom panels: composite image of FISH and phalloidin immunostaining. Arrowheads point to *MKK7* mRNA. FISH probe was directed against the 3′-UTR2 region. (B) Sense MKK7 mRNA FISH control. Fluorescence intensities were scaled as in (A). (C) Confocal fluorescence micrographs of neurons immunostained for different components. Images are color coded for fluorescence intensity so that warm and cold colors represent high and low signal intensity, respectively. F-actin panels are shown in ibw contrast. (D) Averaged fluorescence intensity profiles along chosen neurites. Red dotted lines represent the soma/neurite interface whereas blue dotted line represents neurite/growth cone interface. Fluorescence images were subjected to line scan analysis with a 70-µm long line. The mean ± SEM of normalized fluorescence intensity profiles from ten neurites are shown. Scale bars: 10 µm.

**Figure 9:**
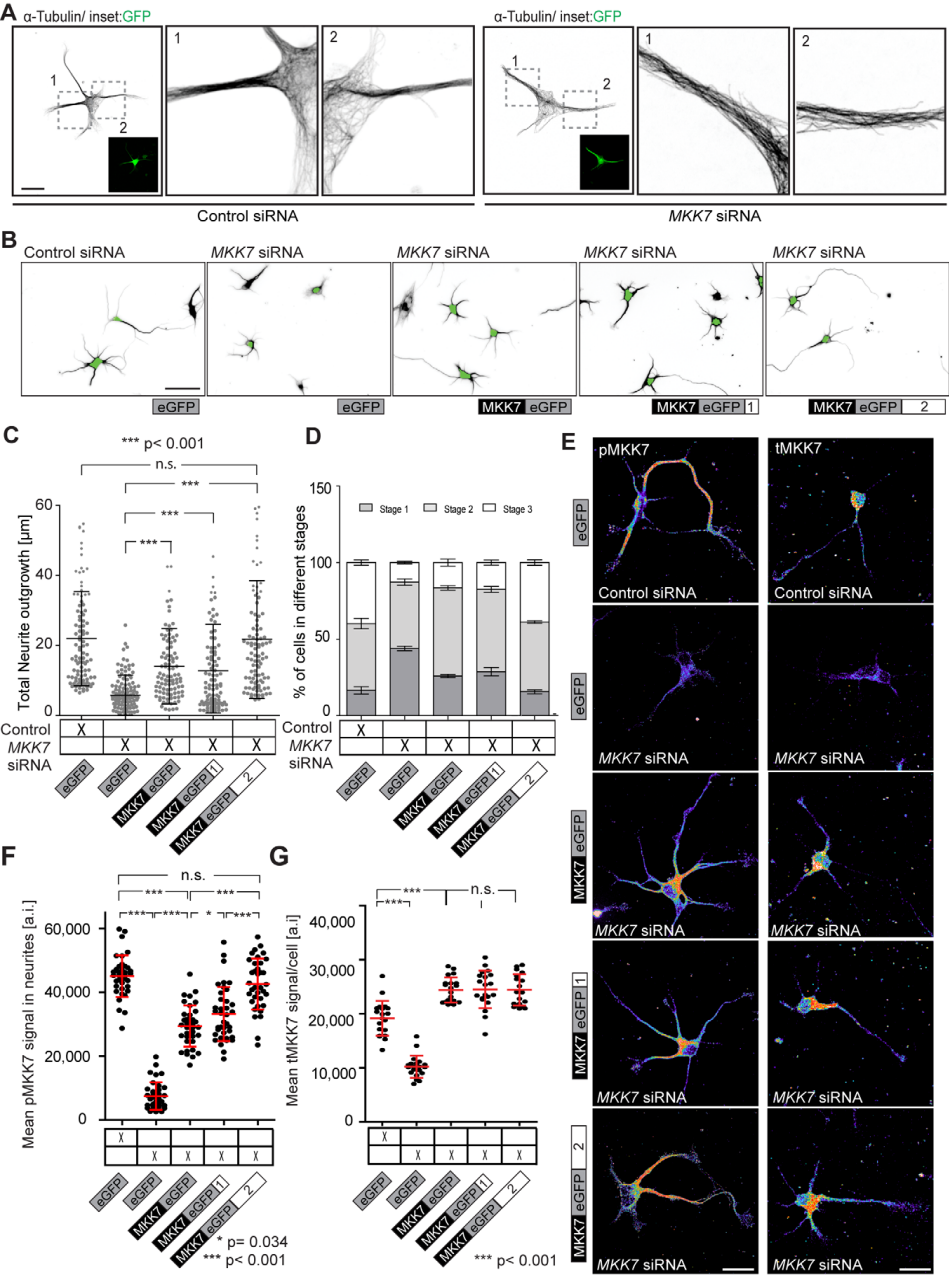
MKK7 function in hippocampal neurons. (A) Representative high resolution micrographs of α-tubulin-stained control or *MKK7* siRNA transfected cells. E18 hippocampal neurons were co-electroporated with a control or *MKK7* siRNA pool and a GFP expression vector, allowed to attach to a poly-D-lysine coated coverslip and fixed a DIV 1. Inset represents GFP signal identifying transfected cells. Note loss of mt bundling in the *MKK7* KN cells. Scale bar: 10 µm. (B) Representative micrographs of control or *MKK7* KN hippocampal neurons rescued with plasmids encoding *GFP*, *MKK7-GFP/-*, *MKK7-GFP/3′-UTR1*, and *MKK7-GFP/3′-UTR2* mRNAs. E18 hippocampal neurons were co-electroporated with a control or *MKK7* siRNA and/or different MKK7-GFP constructs and allowed to attach to a PLL-coated coverslip and fixed a DIV 2. Cells were immunostained for α-tubulin and GFP. α-tubulin is shown in ibw contrast. Green soma signal represents immunostained GFP signal to identify transfected cells. This was also used to mark the soma for automated neurite segmentation and outgrowth analysis. Scale bar: 20 µm. (C) Total neurite outgrowth analysis of GFP-positive cells shown in (B). Measurements from *n* = 150 cells are shown. (D) Axon specification status analysis of GFP-positive cells shown in (B). Number of cells were visually scored as stage 1 (cells with lammelipodial and filopodial protrusions), stage 2 (cells with multiple immature neurites), or stage 3 (asymmetric cells with presence of one specified axon). Approximately *n* = 50 cells in three different stitched fields of view from one specific experiment were scored (total of about *n* = 150 cells scored/experiment). SDs are shown. (E) Representative micrographs of tMKK7 and pMKK7 signals in the MKK7 rescue experiment. Images are color-coded so that warm and cold colors represent high and low pMKK7 or tMKK7 signal. Cells were stained with anti tMKK7 or pMKK7 antibodies, imaged and fluorescence intensities scaled with identical conditions within one experiment. Images were acquired with a confocal microscope with a maximally open pinhole for adequate signal quantification. Scale bars: 10 µm. (F) Quantification of pMKK7 signals in the neurite. Mean neurite fluorescence intensities per neurite are shown. Only GFP-positive cells were considered. Note robust neurite pMKK7 signal recovery with MKK7-GFP/3-UTR1 and 3′UTR2 constructs. *n* = 40 neurites. SD is shown. (G) Quantification of average tMKK7 signals/cell. Note loss of tMKK7 signal with *MKK7* KN and rescue of tMKK7 upon expression of different MKK7-GFP variants. SD is shown.

We then explored if the above described signaling network also operates in E18 mouse hippocampal neurons, which is a well-characterized model system for neurite outgrowth and axonal specification. For that purpose, we used day in vitro (DIV) 1 neurons, a stage at which cells mostly exhibit short neurites that are on the verge of axon specification. *MKK7* mRNA containing punctate structures were prominently enriched in neuronal growth cones whereas faint signal was observed in the soma (Figure 8A and 8B). This was observed with a FISH probe directed against the 3′-UTR2 region. DLK, pMKK7, pJNK T183, pJNK T183Y185, and Map1b localized in all neurites with identical subcellular localizations as in N1E-115 neuronal-like cells (Figure 8C and 8D). We then studied the function of MKK7 in hippocampal neurons. *MKK7* KN led to reduced neurite outgrowth when evaluated in hippocampal neurons at DIV 1 (Figure S18A–S18C). This correlated with loss of the ability of mts to bundle in the neurite (Figures 9A and S18D). These results show that MKK7 performs identical functions in hippocampal neurons and N1E-115 cells.

To address if growth cone *MKK7* mRNA localization is also a relevant mechanism for neurite elongation in hippocampal neurons, we performed *MKK7* rescue experiments. Here, we evaluated the hippocampal neurons at DIV 2 in order to explore any potential effects on axonal specification. Using FISH, we observed that exogenously expressed *GFP* or *MKK7-GFP/-* mRNAs selectively localized to the soma, while *MKK7-GFP/3′-UTR1* and *3′-UTR2* mRNAs displayed punctate signals in the growth cone, as well as diffuse signal in the soma (Figure S19). Rescue with the *MKK7-GFP/-* and *MKK7-GFP/3′-UTR1* mRNAs led to longer neurites, but not to the extent observed in control cells. In contrast, the *MKK7-GFP/3′-UTR2* mRNA enabled full rescue of neurite outgrowth (Figure 9B and 9C). To explore any impact on axonal specification, we scored the relative number of cells in different differentiation states as classically described by Dotti and Banker [36]. We found that *MKK7* KN led to a decrease in the number of neurons that had specified their axon (Figure 9D). Intermediate levels of axonal specification were observed with the *MKK7-GFP/-* and *MKK7-GFP/3′-UTR1* mRNAs, while full rescue was observed with the *MKK7-GFP/3′-UTR2* mRNA (Figure 9D). Importantly, all stage 3 neurons retained the ability of specifying a single axon. Finally, we correlated these responses with the subcellular localization of pMKK7 and tMKK7 in the rescue experiment (Figure 9E and 9F). MKK7 originating from somatically localized *MKK7-GFP/-* mRNA and *MKK7-GFP/3′-UTR1* were aberrantly phosphorylated in the cell soma and to a low extent in the neurite. In contrast, MKK7 originating from *MKK7-GFP/3′-UTR2* mRNA was specifically and robustly phosphorylated in the neurite but not in the soma (Figure 9E and 9F). MKK7 proteins originating from all three mRNAs were mostly localized in the soma, and displayed similar expression levels as endogenous MKK7 protein (Figure 9E and 9G). We also observed that the subcellular patterns of the downstream target pJNK T183 paralleled those of pMKK7 (Figure S20) in the MKK7 rescue experiment. These experiments show a strong correlation between pMKK7 subcellular localization, neurite outgrowth, and axonal specification rescue. These results are however slightly different than in N1E-115 cells. First, in hippocampal neurons, exogenously expressed *MKK7-GFP/3′-UTR1* and *3′-UTR2* mRNAs localize to both the soma and growth cone. Second, only the *MKK7-GFP/3′-UTR2* mRNA allows for robust MKK7 phosphorylation in the neurite and full rescue of neurite outgrowth in hippocampal neurons.

Globally, these results show that growth cone *MKK7* mRNA localization is a conserved mechanism for mt bundling and neurite elongation in both N1E-115 cells and hippocampal neurons.

## Discussion

### Localized Transcriptomes during Neurite Outgrowth

Fine tuning of local proteomes through mRNA localization and translation is a widely used mechanism in neurons. In highly polarized neuronal cells, the general view is that local mRNA translation allows axonal or dendritic compartments to rapidly respond to extrinsic signals such as chemotropic responses or regeneration after injury. Because these compartments are distant from the soma, this allows them to bypass the need for recruitment of somatically translated proteins. Here, we identify a set of 80 mRNAs that localize to neuronal processes during the initial neurite outgrowth process. This suggests that even in these relatively undifferentiated cellular compartments, in which diffusion of somatically translated proteins most likely is not limiting, anchoring of mRNAs at specific subcellular regions and, possibly, their local translation plays a role in cell morphogenesis. Consistently, it is important to note that mRNA translation paradigms have also been observed in cell systems in which diffusion of cytosolically translated proteins is not limiting. One important example is β*-actin* mRNA translation at the leading edge of motile fibroblasts [37].

These mRNAs encode a large variety of different signaling, cytoskeletal, motor, and trafficking proteins that are consistent with local functions in the neurite. Some of these mRNAs have also been found in fibroblast lamellipodia [31], in axons from retinal neurons [10], and axons from embryonic or adult sensory neurons (Table S1) [11]. Globally, these studies hint that complex local transcriptomes exist in neuronal processes that will need to be further studied.

### *MKK7* mRNA Localization Regulates Spatio-Temporal Activation of a JNK Signaling Network Controlling mt Bundling and Neurite Elongation

We explored the function of growth cone localization of an mRNA that encodes MKK7, a MAPKK for JNKs. We found that *MKK7* mRNA localization is essential for its selective phosphorylation and activation within the neurite. At this location, it functions within a spatio-temporal JNK signaling network that we identify to contain DLK (MAP3K), MKK7 (MAP2K), and JNK1 (MAP1K) leading to MAP1b phosphorylation, which then controls mt bundling in the neurite to allow its elongation.

Evidence for this spatio-temporal signaling mechanism stems from the observations that the products of *MKK7* mRNA engineered to have somatic and/or growth cone localization are phosphorylated at these specific subcellular localizations. In our N1E-115 rescue experiment, unlike in wild-type (wt) cells, somatically localized *MKK7* mRNA (originating from the MKK7-GFP/- construct) is aberrantly phosphorylated in the soma. Some weak neurite pMKK7 signal is also observed in the neurite, most likely resulting from diffusion from the soma, and might allow for the limited rescue of the neurite elongation phenotype. In contrast, *MKK7* mRNA with robust growth cone localization (originating from the MKK7-GFP/3′-UTR2 construct) leads to exclusive phosphorylation in the neurite, and allows for robust rescue of neurite elongation. Here, the specific pMKK7 signal also exhibits the characteristic gradient (high at the neurite base decreasing towards the growth cone) as observed in wt cells. Finally, the *MKK7* mRNA species containing the 3′-UTR1, which localizes to both the growth cone and the soma, leads to MKK7 phosphorylation at both subcellular localizations and also enables full rescue of neurite elongation. Identical results are observed with pJNK T183, which is the substrate of MKK7, clearly showing that spatio-temporal control of MKK7 activation is transmitted to downstream signaling.

This mechanism for spatio-temporal control of MKK7 activity is also observed in primary hippocampal neurons, with the difference that the *MKK7* mRNA species containing the 3′-UTR1 does neither rescue neurite pMKK7 and pJNK T183 signals, nor robust neurite elongation. One possible explanation for this difference is that MKK7 proteins are expressed at high level in N1E-115 cells (Figure S8B and S8C), but to near endogenous levels in hippocampal neurons (Figure 9G). Overexpression of the *MKK7-GFP/3′-UTR1* mRNA might therefore provide functional compensation and allow the strong neurite pMKK7 signal in the N1E-115 cells, concomitant with rescue of neurite outgrowth.

### Local *MKK7* mRNA Translation as a Mechanism for Spatio-temporal Control of MKK7 Phosphorylation

The most likely mechanism that explains how growth cone *MKK7* mRNA localization allows MKK7 phosphorylation in the neurite, is that it is translated at this location and subsequently phosphorylated. This is supported by the finding that the *MKK7* mRNA 3′-UTR1 and 3′-UTR2 sequences not only allow growth cone mRNA localization but also translation. Furthermore, our rescue experiments with CI MKK7 clearly show that both the *MKK7* 3′-UTR mRNA sequences, and MKK7 activity are necessary for proper neurite elongation, excluding an independent role for the 3′-UTR. No obvious difference in MKK7 protein levels and subcellular localization were observed with rescue of the *MKK7-GFP/-*, */3′-UTR1*, or */3′-UTR2* mRNAs. This suggests that only a small pool of MKK7 is locally translated (and phosphorylated), which has been previously observed for β*-actin* mRNA in axons [38]. We were however not able to formally show that local mRNA translation is necessary for selective neurite MKK7 phosphorylation and subsequent neurite elongation using translation inhibitors. Bulk application of anisomycin did not affect pMKK7 signals in a time window of 1 h, possibly reflecting the existence of a stable pMKK7 signaling complex, until the N1E-115 cells died (unpublished data). At the early differentiation stage we are studying, it is not possible to fluidically isolate neurites using microfluidic devices [39] to selectively apply translation inhibitors to the growth cone. In the future, this might be studied using novel chemical biology techniques that can specifically label newly synthesized proteins [40].

**Figure 10:**
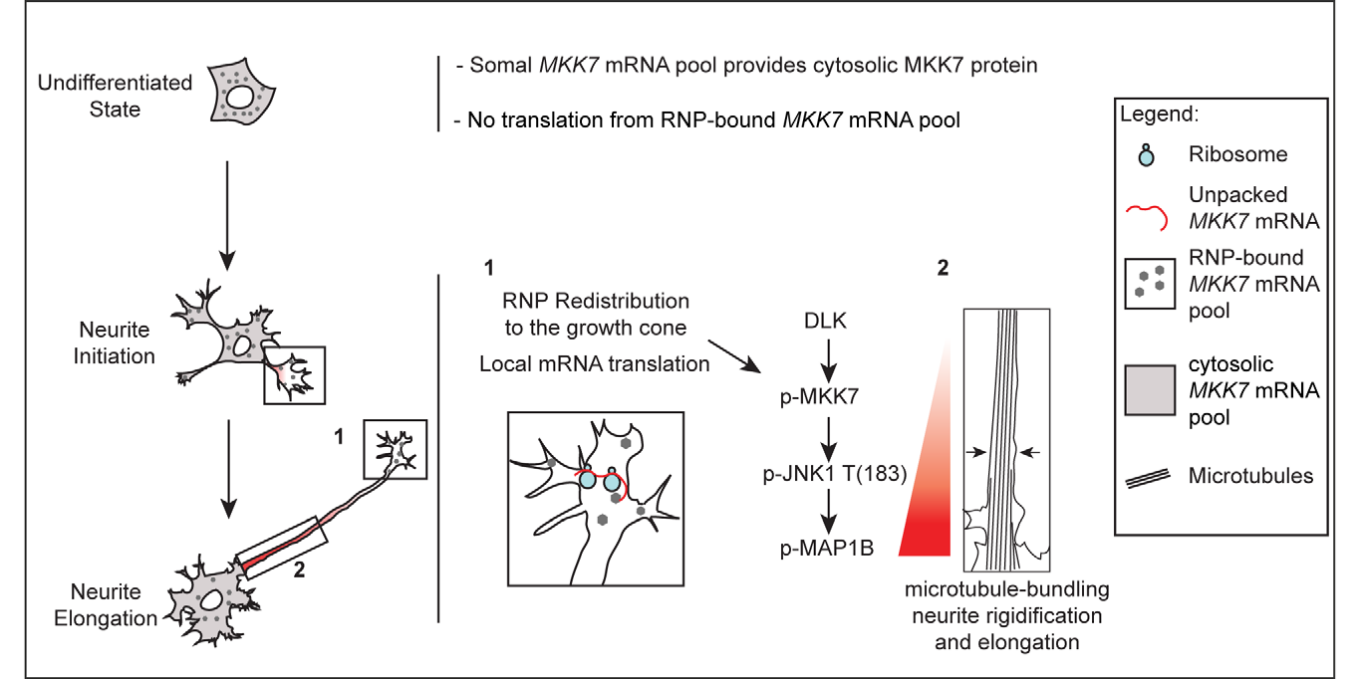
Model of localized JNK signaling during neurite elongation. In the undifferentiated state, in the absence of growth cones, RNP-tethered, most likely 3′-UTR1 and 3′-UTR2 containing *MKK7* mRNAs remain in a translation incompetent state. A somal *MKK7* mRNA pool, most likely containing the 3′-UTR1 sequence, might allow production of the cytosolic MKK7 protein pool that can be engaged for stress responses. During neurite initiation, *MKK7* mRNA containing RNPs are transported to the growth cone where local translation occurs. Local MKK7 synthesis and its phosphorylation by DLK allows to switch on JNK1, MAP1b phosphorylation, and mt bundling to ultimately allow neurite elongation.

Our results show that growth cone *MKK7* mRNA localization, and most likely translation, is a mechanism for spatio-temporal regulation of JNK signaling. We present a model on how this mechanism allows regulation of neurite outgrowth during initial differentiation (Figure 10). This mechanism allows for several important features for the regulation of neurite outgrowth.

First, our results suggest that growth cone *MKK7* mRNA translation provides a mechanism to specifically position mt bundling activity in the neurite necessary for its rigidification and proper elongation. This seems also to be important for generation of the pMKK7 gradient, which in turn allows high MAP1b phosphorylation at the base of the neurite. This might allow robust mt stabilization and stiffness at this location versus more flexibility in the neurite distal region that might be important for growth cone motility. In that respect, it will be important in the future to explore the molecular mechanisms that allow retention of pMKK7 in the neurite and the formation of the characteristic pMKK7 gradient. One potential mechanism might be that growth cone translated MKK7 protein diffuses throughout the neurite and gets phosphorylated by DLK, which displays an identical subcellular localization than pMKK7. DLK might therefore be a key player that shapes the specific pMKK7 signaling zone. However, the observation that somatically-translated *MKK7* mRNA cannot reproduce the characteristic pMKK7 gradient implies that there are additional mechanisms, controlled by MKK7 mRNA localization, that regulate the diffusion of this pMKK7 pool so that it remains in the neurite. Furthermore, our experiments with CA MKK7 formally show that *MKK7* mRNA growth cone localization is essential for MKK7 protein function independently of simply being in an activated state. One possibility is that locally translated and phosphorylated MKK7 is endowed with specific post-translational modifications and/or ability to interact with particular scaffolds that tether it to the neurite. One can also imagine the local action of MKK7-specific phosphatases at the base of the neurite. However, the underlying signaling machinery allowing this spatio-temporal regulation remains elusive since our perturbation studies targeting a potential MKK7-JNK interactome (including scaffold proteins and phosphatases), suggested by our previous proteomic analysis of the neurite [23], did not yield any significant neurite outgrowth phenotype. Globally, these results suggest that the function of *MKK7* mRNA localization and translation is the formation of a specific signaling complex that can activate a precise spatio-temporal MAPK module.

Second, while local mRNA translation has been mostly studied in the context of acute chemotropic or neuronal injury responses in axons, the mechanism we report here might continuously occur during neurite outgrowth. We propose that local *MKK7* mRNA localization and translation allows for a cell-geometry dependent mechanism to switch on the JNK pathway during neuronal differentiation. Here, simple production of a growth cone, by providing a platform for *MKK7* mRNA translation, will trigger and appropriately position pMKK7 and the specific MAPK module leading to mt bundling. In that respect, there is evidence that local mRNA translation is coupled with adhesion signaling which is specifically taking place in the growth cone. Ribosomes, RNA binding proteins and mRNAs have been shown to localize to adhesion complexes [41],[42] and regulation of β*-actin* mRNA translation is regulated by Src-dependent phosphorylation of zipcode binding protein in neurites [43]. An important consequence of a growth cone mediated adhesion signal that switches on local MKK7 mRNA translation, is that simple growth cone removal due to neurite collapse is susceptible to switch off this specific spatio-temporal JNK signaling network. Consistently, we observe that neurite JNK activation is lost during growth cone collapse using the JNKAR FRET probe (unpublished data). Thus, this might allow to co-ordinate growth cone de-attachment with mt unbundling in the neurite shaft during collapse. Finally, it is important to mention that *MAP1b* mRNA has also been observed in neuronal processes [44]. This suggests that additional components of the spatio-temporal JNK signaling network we have defined might also be subjected to regulation by mRNA localization and translation.

Third, growth cone *MKK7* mRNA localization and translation as a mechanism to segregate active JNK in the neurite also provides an elegant mechanism for its uncoupling from nuclear translocation and activation of transcription, which typically occurs in response to cellular stress or neuronal injury. Importantly, in the latter process, retrograde transport of JNK containing signaling endosomes from the axon to the nucleus are used as a mechanism to switch on stress responses [14]. An interesting question is therefore if neurite-tethered active JNK also represents a reservoir that can be mobilized for retrograde signaling in response to neuronal injury.

### Function of *MKK7* mRNA Species with Distinct 3′-UTRs

As mentioned above (Figure S5), the MKK7 locus produces multiple mRNAs that contain either the short 3′-UTR1 or the long 3′-UTR2, and encode different MKK7 isoforms. It will be important to explore the structural nature of the *MKK7* mRNA 3′-UTR1 and 2 determinants, as well as the RNA binding proteins that control *MKK7* mRNA transport and local translation. Our preliminary bioinformatic analysis was not able to pinpoint any RNA motif in the *MKK7* mRNA 3′-UTRs that could bind to specific RNA binding proteins. Importantly, the 3′-UTR2 containing mRNAs are generated by retention of an intron, which has recently been shown to occur in a panel of dendritically targeted mRNAs [45]. A correlate of this is that the 3′-UTR1 is embedded within the 3′-UTR2 sequence. Thus, because both 3′-UTR1 and 3′-UTR2 sequences lead to MKK7 mRNA localization, this suggests that 3′-UTR1 might contain a minimal growth cone targeting motif.

What might be the functional significance of two different 3′-UTRs? The observation that 3′-UTR1 containing MKK7 mRNA species are predominantly localized in the soma suggests that they provide the bulk of cytosolic MKK7 that can be used during stress responses irrespectively of the differentiation state. The more diffuse staining observed with chimeric 3′-UTR1 constructs suggests that not all mRNA is bound to RNPs. A subset of the 3′-UTR1 containing *MKK7* mRNA species however localizes to the growth cone, and might initiate the JNK network upon growth cone formation during the differentiation process. Such a dual localization pattern (soma and growth cone) is also observed for the β-*actin* mRNA in N1E-115 cells (Figure 1G) and, generally, is thought to occur because RNA binding proteins are limiting for packaging and transport of mRNAs to the periphery [46]. Surprisingly, 3′-UTR2 containing *MKK7* mRNA species robustly localize to the growth cone, and are mostly absent from the soma. It will therefore be interesting to study how this striking localization pattern is achieved. This might involve low expression levels and efficient packaging and/or mRNA decay in the soma. This might be controlled by additional sequences in the 3′-UTR2 compared to the 3′-UTR1. We observe an increase in the level of 3′-UTR2 containing *MKK7* mRNA species during N1E-115 differentiation, suggesting a more specialized function in the regulation of mts.

### Function of Growth Cone *MKK7* mRNA Localization and of the DLK-MKK7-JNK1-MAP1b Signaling Module in Hippocampal Neurons

Evaluation of the subcellular location of the signaling components (in N1E-115 cells and hippocampal neurons), as well as the mapping of the hierarchy in the signaling module (in N1E-115 cells), clearly identifies a DLK-MKK7-JNK1 spatio-temporal signaling module leading to mono-phosphorylation of T183 on JNK, ultimately leading to Map1b phosphorylation. This is consistent with documented biochemical interactions: (1) DLK preferentially phosphorylates MKK7 (versus MKK4) in vitro [47]; (2) MKK7 preferentially phosphorylate T183 (versus Y185) on JNKs [48]; (3) Map1b is a direct substrate of JNK1 [15].

Our results show that growth cone *MKK7* mRNA localization also modulates this JNK signaling module in hippocampal neurons. However, in this cellular context, only 3′-UTR2 containing *MKK7* mRNA seems to contribute to robust neurite outgrowth. The MKK7 rescue experiment clearly shows a strict correlate between neurite length and axonal specification. Furthermore, the DLK-MKK7-JNK1 module clearly operates in each neurite in hippocampal neurons. These results imply that the JNK signaling module does not directly regulate axonal specification, and that the axonal specification phenotypes we observe in response to molecular perturbations are the result of modulation of neurite outgrowth dynamics. This is consistent with experiments using primary neuron cultures isolated from knockout mice that show that loss of DLK [27] or MKK7 [49] impairs stage 1 to 2 transition, and subsequently, axonal specification. Importantly, DLK was specifically linked to JNK1 in this process [27].

Our analysis of additional JNK signaling network components in N1E-115 cells and hippocampal neurons suggests the existence of a second spatio-temporal JNK signaling module that leads to dually phosphorylated JNK T183Y185 specifically in the growth cone, where it co-localizes with pMKK4, JIP1, and pStathmin. Consistently, MKK4 preferentially phosphorylates Y185 (versus T183) on JNKs [48]. In hippocampal neurons, there is clear evidence that both dually phosphorylated JNK T183Y185 [19] and JIP1 [20] become selectively enriched in the axon, and are important for axonal specification. This therefore suggests the existence of a distinct spatio-temporal JNK signaling module solely devoted to regulation of axonal specification, and gives insight about the complexity of spatio-temporal JNK signaling.

In vivo studies show that DLK, MKK7, and JNK1 loss of function animals show defects in axon elongation. Loss of function of each protein seems to affect axon fiber tracts, ultimately leading to defects in commissure formation. Specifically, DLK knockout animals display impaired fiber tract development by neocortical pyramidal neurons in the cerebrum [50]. Conditional genetic ablation of MKK7 in the brain leads to decreased forebrain axon tracts [49]. Finally, in JNK1 knockout mice, axon tracts of the anterior commissure first appear normal until postnatal day 6, but then subsequently degenerate, suggesting a role for JNK1 in axon maintenance [15]. These studies show that defects in the DLK-MKK7-JNK1 signaling module lead to defects in axon outgrowth in vivo.

In short, our results show that localization of mRNAs in the growth cone, and most likely also local mRNA translation also operates during neurite outgrowth, before the axon-dendrite specification step. The function of *MKK7* mRNA growth cone localization, and most likely translation, is the positioning of a specific JNK signaling module with a highly dedicated function in spatio-temporal regulation of the cytoskeleton, allowing “insulation” from other JNK signaling modules.

## Material and Methods

### Cell Culture, Transfection, and Immunofluorescence

N1E-115 neuroblastoma cells (American Tissue Culture Collection) were cultured in Dulbecco's modified Eagle's medium (DMEM) supplemented with 10% FBS, 1% L-glutamine, and 1% penicillin/streptomycin. For differentiation, N1E-115 cells were starved for 24 h in serum-free Neurobasal medium (Invitrogen) supplemented with 1% L-glutamine and 1% penicillin/streptomycin. Cells were detached with PUCK's saline and replated on coverslips previously coated with 10 µg/ml laminin (Millipore-Chemicon). For experiments with primary cells, E18 hippocampal neurons were isolated and plated on coverslips coated with poly-D-lysine. For plasmid transfection, N1E-115 cells were transfected as previously described [51]. For siRNA-mediated KN experiments, 3×10^5^ N1E-115 cells were transfected with 100 pmol of siRNA (Dharmacon siRNA Smartpool Plus) with 6 µl of Dharmafect-2 transfection reagent (Dharmacon) per well (six-well plate) in presence of serum. 48 h post-transfection cells were starved in neurobasal medium. 72 h post-transfection cells were used in the different assays. For combinations of siRNA-mediated KN and plasmid transfection, cells were transfected as previously described [51] and 100 pmol of siRNA were added to the transfection mix. In the MKK7 rescue experiments, a single MKK7-specific siRNA (Invitrogen Stealth Select) was used for KN.

### Neurite Purification, Genechip, and RT-qPCR Analysis

Neurite purification was performed as described elsewhere [23]. For RNA extraction, the Nucleospin RNA II kit (Macherey–Nagel) was used according to manufacturer's protocol. For Western blot analysis, a 1% SDS buffer containing protease inhibitors and 2 mM Vanadate was used. Genechip analysis of soma and neurite total RNA was performed in duplicate. CRNA target synthesis was done starting from 200 ng total RNA using the WT Expression kit (Ambion, In Vitrogen Life Sciences) following standard recommendations. The further steps were performed according to manufacturer's protocol (Affymetrix). To select differently expressed genes a one-way ANOVA model was applied. Genes were filtered on the basis of an adjusted *p*-value lower than 0.01. For RT-qPCR analysis, 1 µg of total mRNA lysate was retrotranscribed to cDNA using the ImProm-II Reverse Transcription System (Promega). RT-qPCR analysis was performed using a SYBR green mix (Applied Biosystems), appropriate primers, and RPL19 primers for normalization.

### Immunofluorescence

N1E-115 cells were washed with PBS, fixed in 80 mM PIPES, 1 mM MgCl2, 1 mM EGTA, pH 6.8 containing 0.25% glutaraldehyde for 45 s and permeabilized in the same buffer containing 0.1% Triton-X for 10 min. Coverslips were incubated with 0.2% sodium borohydride in PBS for 20 min, and blocked in 2% BSA, 0.1% Triton-X in PBS for 15 min. Cells were stained with primary antibodies for 1 h, and then with secondary antibodies for 30 min (Alexa-fluor 488 labeled phalloidin, Alexa-fluor 546 secondary antibody, and DAPI for 30 min [all Invitrogen]). For all the other immunofluorescence experiments, N1E-115 cells or DIV 1 mouse hippocampal neurons were washed in PBS, fixed in PBS containing 4% paraformaldehyde (Sigma Aldrich) for 20 min, and permeabilized in PBS containing 1% of Triton-X for 2 min. Coverslips were then washed, blocked, stained, and mounted as described above.

### FISH

Labeled probes were generated by in vitro transcription from restriction-digested plasmids using the DIG RNA labeling mix (Roche) according to the manufacturer's protocol. The FISH protocol is described elsewhere [52] and was adapted with the following changes: probes (1 ng/µl) were heated to 70°C for 7 min and incubated on ice for 2 min before applying to fixed cells; after overnight hybridization at 65°C and extensive washes in PBS-0.1% Tween, cells were blocked in blocking buffer (2% BSA PBS-Tween) for 2 h; detection by TSA-Alexa488 or TSA-Alexa546 (Invitrogen) were performed according to the manufacturer's protocol.

### Microscopy, Image Acquisition, and Analysis

All wide field microscope experiments were performed on an inverted Eclipse Ti microscope (Nikon). Phase contrast live imaging of neurite dynamics: N1E-115 cells were replated on laminin-coated glass-bottom multi-well plates (MatTek). 3–4 h after plating, cells were imaged in Neurobasal medium (Invitrogen) in a heated closed chamber. GFP-tubulin, JNKAR FRET, and PalX2-dendra2 live cells imaging experiments: serum-starved, N1E-115 cells transfected with the different constructs were replated on laminin-coated coverslips for different times and imaged in Neurobasal medium supplemented with 10 µg/ml oxyrase reagent (Oxyrase Inc.) in a closed chamber. FRET ratio imaging was performed as described elsewhere [53]. In the bleaching experiments, a FRAP3D module (Roper Scientific) was used to bleach a region of interest with 488 nm laser light. Neurite outgrowth analysis: automated neurite segmentation was performed using Metamorph software. For confocal imaging of fixed, stained samples, a Leica TCS SP5 confocal microscope steered was used.

### Bioinformatic and Statistic Analysis

The JNK signaling network was extracted from our previously published neurite proteome dataset [23], using Ingenuity pathways software (Ingenuity Systems). Only proteins that were enriched in the neurite or found in the neurite and soma fractions were considered. MKK7, MKK4, JNK1, JNK2, and JNK3 were used as “bait” to discover proteins that can directly interact with them. Statistical analysis was performed using GraphPad Prism 5 software (Mozilla Labs). For multiple comparisons one-way ANOVA with Dunnet test with 95% confidence intervals was used, for single comparisons a two-tail unpaired *t*-test was used.

### Antibodies and Plasmids

For Western blot and immunofluorescence experiments the following antibodies were used: anti-MKK7, anti-JNK1, anti-JIP1, anti-JIP3 (all Santa Cruz Biotechnology), anti-phospho-MKK7 (S271+T275), anti-phospho-JNK (T183+Y185), anti-phospho-JNK (T183), anti-DLK, anti-phospho-MKK4 (S257+T161), anti-phospho-doublecortin (S28), anti eEF1A1, anti-GluTubulin (all Abcam), anti-phospho-stathmin (S38), anti-phospho-MAP2 (S136) (all Cell Signaling), anti-MAP2 (Millipore), anti-α-tubulin, anti-MAP kinase activated (T183−Y185), anti-ERK1 (Sigma Aldrich), anti-GFP (Roche), Alexa Fluor350 phalloidin, Alexa Fluor488 phalloidin, Alexa Fluor555 phalloidin (all Invitrogen), DAPI (Sigma Aldrich). Anti-phospho-MAP1b was a gift from Fatiha Nothias (Universite Pierre et Marie Curie, Paris). GFP-MKK7 (gift of Jiyan Zhang, Institute of Basic Medical Science, Beijing) was mutated to confer siRNA resistance. GFP-MKK7, β-globin (gift of Ian G. Macara, University of Virginia, Charlottesville), and PalX2-Dendra (gift of Gary Bassell, Emory University, Atlanta) reporters in eukaryotic expression vectors were all flanked at the 3′ with the *MKK7* 3′-UTR 1 and 2 sequences. JNKAR1 and JNKAR1(T/A) genetically encoded FRET probes were a kind gift of Jin Zhang (John Hopkins University School of Medicine, Baltimore). Detailed construct maps are available on request.

### Primary Neurons Isolation and Transfection

Primary neurons were isolated from mouse embryos at E18. In brief, hippocampi were dissected, trypsinized for 15 min, and dissociated by trituration. Per condition 500,000 cells were transfected with 1 µg plasmid DNA and/or 50 pmol Accell siRNA using the Amaxa Nucleofector I system according to the manufacturer's instructions. Subsequently, cells were plated onto coverslips coated with poly-D-lysine and analyzed after 1 or 2 DIV. For experiments in which CMV promoter-based expression vector was used, low expression levels necessitated anti-GFP immunostaining to identify transfected cells. Coverslips were then scanned using high content microscopy. Neurite outgrowth measurements were performed using metamorph by gating on GFP-positive cells. Staging of differentiation status was performed visually on GFP-positive cells.

## Supporting Information

Figure S1 **Additional validation of neurite-enriched mRNAs by FISH and sense probe controls.** (A) *Trp53INP2* mRNA FISH antisense probe signal. (B) *NET1* mRNA FISH antisense probe signal. (C–H) Sense probe controls for all probes shown in Figures 1 and S1. All samples were stained at the same time than their antisense counterpart, and imaged with identical light conditions. Scale bars: 25 µm.
(TIF)

Figure S2 **Quantification of the spatial patterns exhibited by phosphorylated forms of different JNK signaling network components (and of total DLK).** Confocal fluorescent micrographs of differentiated N1E-115 cells immunostained for different components with similar neurite length were subjected to line scan analysis with a 500-µm long line. The mean ± SEM of normalized fluorescence intensity profiles from ten neurites are shown. The red vertical dotted line denotes the soma/neurite interface. (A) pMKK7. (B) pJNK T183. (C) pJNK T183 Y185. (D) pMAP1B. (E) pDoublecortin. (F) DLK.
(TIF)

Figure S3 **Subcellular localization of additional components of the neurite JNK signaling network.** Representative confocal fluorescent micrographs of differentiated N1E-115 cells immunostained for different components are shown. Images are shown with color-coded fluorescence intensities (warm and cold colors represent high and low fluorescence intensities, respectively). F-actin (phalloidin staining) images are shown in ibw contrast. (A) pMKK4 and tMKK4. Note inverse distribution compared with pMKK7 with high signal intensity in the growth cone decreasing in the neurite (pointed to by arrowhead). Substantial pMKK4 signal is also found in the soma (pointed to by arrowhead). (B) pJNK T183Y185. Note high signal in the growth cone (pointed to by arrowhead), low signal in the neurite. (C) pStathmin. Note high signal in the growth cone (pointed to by arrowhead), low signal in the neurite. (D) JIP-1. Note high signal in the growth cone (pointed to by arrowhead), low signal in the neurite. (E) JIP-3. Note high signal in the growth cone and the soma (pointed to by arrowhead), low signal in the neurite (pointed to by arrowhead). (F) pDoublecortin. Note identical signal intensity in the soma and the neurite. The three arrowheads point to homogeneous signal intensity in the neurite. Scale bars: 25 µm.
(TIF)

Figure S4 **Further characterization of the effect of** ***MKK7*** **KN on cell morphology and the cytoskeleton.** (A) Effect of MKK7 KN on N1E-115 cell morphology in the undifferentiated and differentiated state. Undifferentiated and differentiated cells were plated on laminin-coated coverslips and allowed to adhere for 24 h (F-actin phalloidin staining of control and *MKK7* KN cells are shown (ibw contrast)). Scale bar = 50 µm. (B) Cell surface measurements of (A). Mean ± SD is shown. *n* = 60 cells. (C) Epifluorescence micrographs of the mt cytoskeletons of growth cones of control and *MKK7* KN cells. Adherent growth cones in the morphodynamic state of protrusion were chosen. Top panels: α-tubulin stained growth cones. Bottom panels: manual tracings of mt ends. (D) Quantification of number of mt ends in the growth cone as shown in (C). Mean ± SD is shown. *n* = 20 growth cones. (E) Confocal micrographs of the centrosome in control and *MKK7* KN cells. Cells were immunostained for α-tubulin and pericentrin. ibw contrast is shown. Scale bar = 10 µm. Note the presence of one centrosome in both control and *MKK7* KN cells. This was observed in all cells of *n* = 20 control and *MKK7* KN cells. (F) Close-ups of pictures shown in (E) illustrating the unbundling phenotype. Scale bar = 10 µm.
(TIF)

Figure S5 **Identification of determinants that allow** ***MKK7*** **mRNA localization.** (A) Schematic of the 3′ end of MKK7 gene structure from the ENSEMBL database. Alternative splicing creates transcripts with either 3′-UTR1 (138 nt depicted in green) or 3′-UTR2 by retention of the intron between exons 13 and 14 (depicted in red). Thus, the 3′-UTR2 region also contains the 138-nucleotide stretch from 3′-UTR1. Termination codons (*) and polyadenylation sites (o) are indicated. (B) Relative expression levels of 3′-UTR2 containing mRNAs in non-differentiated and differentiated N1E-115 cells. RT-qPCR with 3′-UTR2 specific primers was performed on equal amounts of total mRNA from N1E-115 cells in the non-differentiated and differentiated state. *n* = 3 experiments. Mean ± SD is shown. *p*-value = 0.03. (C) Human β-globin chimeric constructs schematics. Black boxes, exons; black lines, introns; white boxes, 3′-UTR. (D) Subcellular localization of exogenously expressed constructs. The different constructs were transiently transfected in N1E-115 cells of which neurite and soma fractions were purified. Equal neurite and soma mRNA amounts were then probed by RT-qPCR to determine relative enrichment in each fraction using human β-globin-specific primers. Note that a 3′-UTR sequence of the *RhoA* mRNA, which our genome-wide assay was not identified to be neurite-enriched, did not lead to β-globin mRNA neurite enrichment. *n* = 3 experiments. Mean ± SD is shown.
(TIF)

Figure S6 **Representative FISH micrographs of exogenously expressed** ***GFP*** **,** ***MKK7-GFP/-*** **,** ***MKK7-GFP/3′-UTR1*** **, and** ***MKK7-GFP/3′-UTR2*** **mRNAs.** Representative confocal fluorescence micrographs of *GFP* mRNA FISH in differentiated N1E-115 cells. Images from non-transfected or cells transfected with plasmids encoding *GFP*, *MKK7-GFP/-*, *MKK7-GFP/3′-UTR1*, and *MKK7-GFP/3′-UTR2* are shown. Fluorescence intensity in all images are scaled identically and shown in ibw contrast. An outline of the cell generated using phalloidin staining in another channel is shown. Green arrows point to growth cone, red asterisks point to somata. Scale bar: 30 µm.
(TIF)

Figure S7 **Additional** ***MKK7*** **mRNA rescue experiments: evaluation of mt bundling.** (A) Representative high resolution confocal fluorescence micrographs of α-tubulin stained N1E-115 cells of the MKK7 KN rescue experiment. Ibw contrast is shown. Cells were fixed at 8 h post-replating, allowing for short neurites in all experimental conditions, and fair comparison between wt, MKK7 KN, and rescued cells. (B) Quantification of images observed in (A). Percentage of neurites with bundled/unbundled mts are shown. SEM from two sets of quantifications of 30 cells are shown. Approximately, four neurites/cell were evaluated.
(TIF)

Figure S8 **Additional** ***MKK7*** **mRNA rescue experiments: neurite outgrowth measurements, quantification of tMKK7 expression levels and subcellular localization.** (A) Neurite outgrowth measurements of differentiated N1E-115 cells overexpressing *MKK7-GFP/-*, *MKK7-GFP/3′-UTR1*, and *MKK7-GFP/3′-UTR2* mRNAs. Measurements from 10% cells with longest neurites are shown, *n* = 600 cells. (B) Western blot analysis of expression of endogenous MKK7 (anti-MKK7 antibody) and exogenously expressed MKK7-GFP (anti-GFP) in experiments shown in Figures 5 and S7A. ERK1 serves as loading control. (C) Characterization of tMKK7 fluorescence intensities in control, *MKK7* KN cells rescued with different MKK7 constructs. Absolute fluorescence intensities are shown. Mean ± SD is shown. Note that fluorescence signals are shown in two separate graphs that have been scaled differently so as to highlight relative fluorescence (expression) levels with the adequate dynamic range. (D) Representative tMKK7 micrographs of differentiated N1E-115 MKK7 KN cells rescued with different exogenously expressed MKK7-GFP constructs. Associated line scans are also shown. Images are color-coded so that warm and cold colors represent high and low pMKK7 signal. Cells were stained, imaged, and fluorescence intensities scaled with identical conditions within one experiment. Images were acquired with a confocal microscope with a maximally open pinhole for adequate signal quantification. Absolute fluorescence intensities for the line scans are shown. Note that y-axis is scaled differently for specific experiments. Red, vertical dotted line represents soma/neurite interface. Scale bar: 50 µm.
(TIF)

Figure S9 **Additional** ***MKK7*** **mRNA rescue experiments: rescue of** ***MKK7*** **KN phenotype with CA and inactive forms of MKK7.** (A) Representative micrographs of differentiated N1E-115 *MKK7* KN cells rescued with different exogenously expressed wt, CA, and CI forms of MKK7-GFP/-, *MKK7-GFP/3′-UTR1*, and *MKK7-GFP/3′-UTR2* mRNAs. Cells were immunostained for α-tubulin and are shown in ibw contrast. Scale bar: 100 µm. (B) Neurite outgrowth measurements from micrographs in (A) are shown. Measurements from 25% cells with longest neurites are shown, *n* = 100 cells.
(TIF)

Figure S10 **Additional** ***MKK7*** **mRNA rescue experiments: quantification of pMKK7 subcellular patterns.** (A) Representative pMKK7 micrographs of differentiated N1E-115 *MKK7* KN cells rescued with different exogenously expressed MKK7-GFP constructs as shown in Figure 5D. Associated line scans are also shown. Absolute fluorescence intensities for the line scans are shown. Red, vertical dotted line represents soma/neurite interface. Scale bar: 25 µm. (B) Quantification of soma pMKK7 signals. Mean fluorescence intensity per soma are shown. Error bars represent SD, *n* = 20 cells.
(TIF)

Figure S11 **Additional** ***MKK7*** **mRNA rescue experiments: effect on downstream pJNK T183 signals.** (A) Representative micrographs of pJNK T183 signals of differentiated N1E-115 *MKK7* KN cells rescued with different exogenously expressed MKK7-GFP constructs. Images are color-coded so that warm and cold colors represent high and low pMKK7 signal. Cells were stained, imaged, and fluorescence intensities scaled with identical conditions within one experiment. Images were acquired with a confocal microscope with a maximally open pinhole for adequate signal quantification. Scale bar: 25 µm. (B) Quantification of pJNK T183 signals in the neurite. Mean neurite fluorescence intensities per neurite are shown. Note robust neurite pMKK7 signal recovery with MKK7-GFP/3-UTR1 and 3′UTR2 constructs. Error bars represent SD, *n* = 20 cells. (C) Quantification of pJNK T183 signals in the soma. Mean soma fluorescence intensities per cell are shown. Error bars represent SD, *n* = 20 cells.
(TIF)

Figure S12 **Additional controls for Palx2-Dendra2 reporter constructs.** (A) Representative micrographs of PalX2-Dendra2/-, 3′-UTR1, 3′-UTR2 reporters in live growth cones without any bleaching procedure. Images are presented in ibw contrast. Scale bar: 5 µm. Note homogeneous distribution of fluorescence signal. (B) Quantification of PalX2-Dendra2/-, 3′-UTR1, 3′-UTR2 reporters expression levels. Average fluorescence intensities/cell are shown. Mean ± SD is shown.
(TIF)

Figure S13 **Neurite-localized JNK network siRNA screen.** (A) Total neurite outgrowth measurements. Total neurite outgrowth length measurements of the 10% cells with longest neurites in response to KN of different gene products are shown. Results of three independent experiments are shown. Statistical significance is shown. In all the experiments mean ± SD is shown, *n* = 400 (experiment 1), *n* = 340 (experiment 2), *n* = 500 cells (experiment 3). (B) Averaged neurite measurements from the three experiments (A) expressed as percent change of control. Mean ± SD and statistical significance are shown. (C) Representative neurite outgrowth phenotypes. Representative micrographs of α-tubulin immunostained control of siRNA-transfected cells in ibw contrast. Example from one representative experiments is shown. Scale bar: 100 µm. (D) Assessment of KN efficiency. Western blot analysis of relative protein level in equal amounts of lysates of cells transfected with a non-targeting control or specific siRNA are shown. Quantification of KN efficiency normalized to ERK1 loading control are also shown.
(TIF)

Figure S14 **Hierarchy mapping of signaling components in the MKK7 signaling module:** ***DLK*** **KN.** Control and *DLK* KN N1E-115 cells were allowed to differentiate on laminin-coated coverslips and were immunostained for pMKK7, pJNK T183, pMAP1b, or pMKK4. Images were then acquired with confocal microscope with identical exposure settings and with a maximally open pinhole for adequate comparison of fluorescence intensities. Only cells with short neurites were considered for a fair comparison. Left panels: representative images of control and KN cells are shown. Immunostain signal intensity is color-coded so that warm and cold colors represent high and low signals, respectively. Fluorescence micrographs have been scaled identically. Middle panels: average fluorescence intensities on a per cell basis. Right panels: somata and neurite average fluorescence intensities. Middle and right panels: y-axis represents fluorescence intensities. (A) pMKK7. (B) pJNK T183. (C) pMAP1b. (D) pMKK4. Note loss of phospho-signals in (A–C) consistent with the proposed hierarchy in the signaling module. Note that pMKK4 signal is not affected (D). Mean ± SD and statistical significance are shown. Scale bar: 25 µm.
(TIF)

Figure S15 **Hierarchy mapping of signaling components in the MKK7 signaling module:** ***MKK7*** **KN.** Experiments were performed as in Figure S14. (A) pMKK7. (B) pJNK T183. (C) pMAP1b. (D) pMKK4. Note loss of phospho-signals in (A–C) consistent with the proposed hierarchy in the signaling module. Note that pMKK4 signal is not affected (D). Mean ± SD and statistical significance are shown. Scale bar: 25 µm.
(TIF)

Figure S16 **Hierarchy mapping of signaling components in the MKK7 signaling module:** ***JNK1*** **KN.** Experiments were performed as in Figure S14. (A) pMKK7. (B) pJNK T183. (C) pMAP1b. (D) pMKK4. Note absence of loss of pMKK7 in (A) and loss of phospho-signals in (B, C) consistent with the proposed hierarchy in the signaling module. However, pMKK4 (D) is not affected. Mean ± SD and statistical significance are shown. Scale bar: 25 µm.
(TIF)

Figure S17 **Hierarchy mapping of signaling components in the MKK7 signaling module:** ***JNK2*** **KN.** Experiments were performed as in Figure S14. (A) pMKK7. (B) pJNK T183. (C) pMAP1b. (D) pMKK4. Note absence of loss of phospho-signal in (A–D), consistently with no impact on the MKK7 signaling module. Mean ± SD and statistical significance are shown. Scale bar: 25 µm.
(TIF)

Figure S18 ***MKK7*** **KN in hippocampal neurons at DIV 1.** (A) Representative fluorescent micrographs of control or *MKK7* siRNA-transfected E18 embryonal hippocampal neurons. Neurons were fixed at DIV 1. Left panel: GFP marker fluorescence; middle panel: α-tubulin stain in ibw contrast; right panel: α-tubulin signal (black)/neurite segmentation (red). Scale bar: 10 µm. (B) Quantification of neurite outgrowth from *n* = 50 cells. (C) Quantification of tMKK7 signal in GFP-positive cells from *n* = 20 cells. (D) Additional examples of high resolution pictures of tubulin-stained control or *MKK7* siRNA transfected hippocampal neurons. Scale bar: 10 µm.
(TIF)

Figure S19 **Representative FISH micrographs of exogenously expressed** ***GFP*** **,** ***MKK7-GFP/-*** **,** ***MKK7-GFP/3′-UTR1*** **, and** ***MKK7-GFP/3′-UTR2*** **mRNAs in primary hippocampal neurons.** Representative confocal fluorescence micrographs of *GFP* mRNA FISH in primary hippocampal neurons. Images from cells expressing *GFP*, *MKK7-GFP/-*, *MKK7-GFP/3′-UTR1*, and *MKK7-GFP/3′-UTR2* are shown. Cells were co-immunostained with an anti-GFP antibody to provide better signal to noise ratio to identify transfected cells. GFP signal is shown in green. F-actin and FISH signals are shown in ibw contrast and are scaled identically throughout the experiments. Black arrows point to growth cone. Scale bar: 10 µm, 5 µm (close-up).
(TIF)

Figure S20 **Subcellular localization of pJNK T183 in the MKK7 rescue experiment in primary hippocampal neurons.** (A) Representative micrographs of pJNK T183 signals in the MKK7 rescue experiment. Images are color-coded so that warm and cold colors represent high and low pMKK7 or tMKK7 signal. Cells were stained with and anti pJNK T183 antibodies, imaged, and fluorescence intensities scaled with identical conditions within one experiment. Images were acquired with a confocal microscope with a maximally open pinhole for adequate signal quantification. Scale bars: 10 µm. (B) Quantification of pJNK T183 signals in the neurite. Mean neurite fluorescence intensities per neurite are shown. Only GFP-positive cells were considered. Note robust neurite pJNK T183 signal recovery with MKK7-GFP/3′-UTR2 constructs. *n* = 40 cells. SD is shown. (C) Quantification of pJNK T183 signals in the soma. Mean soma fluorescence intensities per cell are shown. Error bars represent SD, *n* = 40 cells.
(TIF)

Table S1 **Neurite-enriched mRNAs identified in genome-wide screen.** Hugo Gene Symbol and Entrez gene names of mRNAs that are enriched more than 1.5 times in the neurite versus the soma fraction are shown. Neurite or soma localization of the products encoded by the different mRNAs are also shown according to our previously published N1E-115 neurite and soma proteomes [23]. Some proteins were not identified in our proteome and are marked as non-available (n/a). Otherwise, the ratio of the spectral peptide count in the neurite versus the soma is shown, with positive values showing neurite enriched and negative values showing soma enriched proteins. For one of the protein, peptides were only found in the neurite (neurite unique). MRNAs that have also been found to be enriched in fibroblast pseudopods [31], in axons from mouse retinal neurons [10], or embryonal or adult sensory neurons [11] are also shown. Finally, the function of the different genes is shown.
(XLSX)

Table S2 **Description of neurite-enriched JNK signaling network genes targeted in the siRNA screen.** Hugo gene symbol and Entrez Gene name and ID are shown along with protein enrichment according to our previously published N1E-115 neurite and soma proteomes [23].
(XLS)

Video S1 **JNK phosphorylation dynamics in differentiating N1E-115 cells.** FRET emission ratio is shown for JNKAR and control, non-phosphorylatable JNKAR probes. Images are color-coded so that warm and cold colors represent high and low levels of JNK activation, respectively. Timescale is in hours∶minutes. Scale bar: 25 µm.
(MOV)

Video S2 **Neurite extension dynamics of control and MKK7-siRNA transfected N1E-115 differentiating cells.** Phase contrast time-lapse imaging of control and MKK7 KN are shown. Timescale is in hours∶minutes. Scale bar: 50 µm. Note highly instable neurite outgrowth in the MKK7 KN cells.
(MOV)

Video S3 **Microtubule dynamics of control and MKK7-siRNA transfected N1E-115 differentiating cells.** Fluorescence confocal microscopy time-lapse videos of control and MKK7 KN cells expressing GFP-tubulin are shown in ibw contrast. In the control cell, arrowhead indicates a neurite that elongates and bundles its mts during this process. In the MKK7 KN cell, arrowhead shows that mts cannot bundle during neurite extension. Timescale is in hours∶minutes. Scale bar: 25 µm.
(MOV)

Video S4 **F-actin dynamics of control and MKK7-siRNA transfected N1E-115 differentiating cells.** Epifluorescence microscopy time-lapse videos of control and MKK7 KN cells co-transfected with Lifeact-GFP are shown in ibw contrast. Timescale is in hours∶minutes. Scale bar: 25 µm.
(MOV)

Video S5 **MKK7 KN rescue experiment I.** Neurite extension dynamics of differentiating N1E-115 cells transfected with: (1) control siRNA and GFP; (2) *MKK7* siRNA and GFP; or (3) MKK7 siRNA and GFP-MKK7/-. Phase contrast time-lapse imaging and GFP fluorescence signal in ibw contrast are shown. GFP channel was acquired every fifth frame. Timescale is in hours∶minutes. Scale bar: 50 µm. Note partial rescue of neurite elongation with the GFP-MKK7/- construct.
(MOV)

Video S6 **MKK7 KN rescue experiment II.** Neurite extension dynamics of differentiating N1E-115 cells transfected with: (1) control siRNA and GFP; (2) *MKK7* siRNA and GFP-MKK7/3′-UTR1; or (3) MKK7 siRNA and GFP-MKK7/3′-UTR2. Phase contrast time-lapse imaging and GFP fluorescence signal in ibw contrast are shown. GFP channel was acquired every fifth frame. Timescale is in hours∶minutes. Scale bar: 50 µm. Note robust rescue of neurite elongation with the GFP-MKK7/3′-UTR1 and GFP-MKK7/3′-UTR2 constructs. Also note highly unstable neurite extension in GFP-negative cells.
(MOV)

Video S7 **Time-lapse imaging of PalX2-Dendra2 reporters.** Time-lapse video of growth cones of N1E-115 cells expressing the PalX2-Dendra2/-, PalX2-dendra2/3′-UTR1, or PalX2-dendra2/3′-UTR2 reporters without any photobleaching. Ibw contrast is shown. Timescale is in minutes. Scale bar: 12 µm. Note homogeneous plasma membrane reporter localization and lack of any vesicular transport.
(MOV)

Video S8 **Visualization of growth cone mRNA translation dynamics using PalX2-Dendra2 reporters.** Time-lapse video of growth cones of N1E-115 cells expressing the PalX2-Dendra2/-, PalX2-dendra2/3′-UTR1. or PalX2-dendra2/3′-UTR2 reporters pre- and post-bleaching are shown. Dendra2 fluorescence signals and differential interference contrast (DIC) images are shown. Fluorescence images are color-coded so that warm and cold colors represent high and low fluorescence intensities. Pre- and post-bleaching images are scaled differently so that signal is not saturated in the pre-bleached state. Note that the bleached region of interest is much larger than the growth cone portion that is shown. Note robust fluorescence recovery of PalX2-Dendra2 reporters flanked with *MKK7* mRNA 3′-UTRs. Timescale is in minutes. Scale bar: 12 µm.
(MOV)

Video S9 **Neurite extension dynamics of control and DLK, JNK1. or MAP1b siRNA transfected N1E-115 differentiating cells.** Phase contrast time-lapse imaging of control and the different KN cells are shown. Timescale is in hours∶minutes. Scale bar: 50 µm. Note highly instable neurite outgrowth in all KN cells.
(MOV)

## Abbreviations

CA: constitutively active
CI: constitutively inactive
DIV: day in vitro
DLK: dual leucine zipper kinase
FISH: fluorescence in situ hybridization
FRET: fluorescence resonance energy transfer
GFP: green fluorescent protein
ibw: inverted black and white contrast
JNK: Jun N-terminal kinase
JNKAR: JNK activity reporter
KN: knockdown
MAP: mt-associated protein
MAP2K: MAP kinase kinase
MAPK: mitogen-activated kinase
MKK: mitogen-activated protein kinase kinase
mt: microtubule
pJNK T183: activated, mono-phosphorylated form of JNK isoforms 1, 2, and 3
pMKK: phosphorylated MKK protein
RNP: ribonucleoprotein particle
RT-qPCR: quantitative PCR coupled with reverse transcription
SD: standard deviation
SEM: standard error of the mean
tMKK7: total MKK7 protein
WT: wild type
