# Supplementary figures and images for: Growth Cone MKK7 mRNA Targeting Regulates MAP1b-Dependent Microtubule Bundling to Control Neurite Elongation
Source: PLoS Biol. 2012 Dec 4;10(12):e1001439. doi: 10.1371/journal.pbio.1001439 (PMC3514283; doi:10.1371/journal.pbio.1001439)

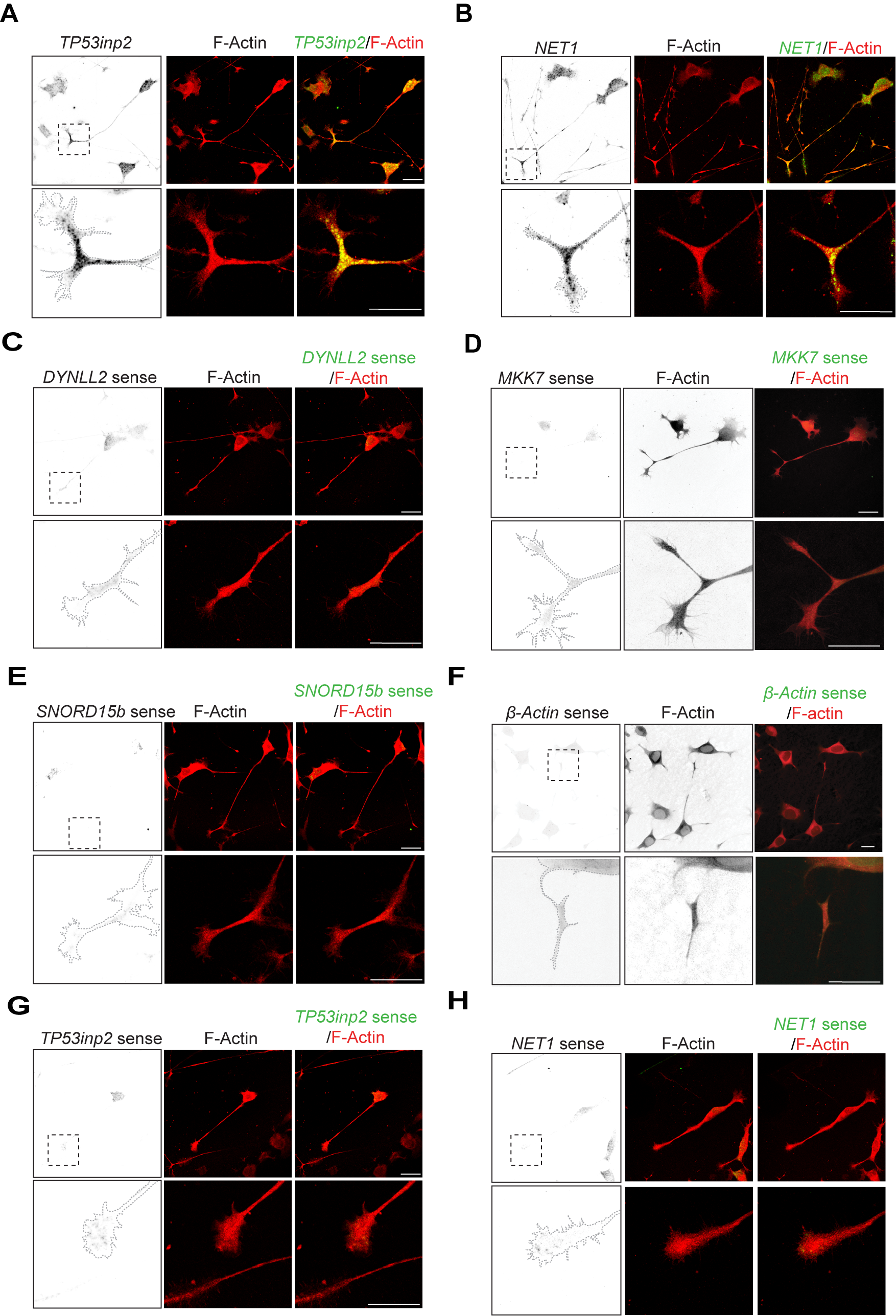

Supplement: Figure S1 — Additional validation of neurite-enriched mRNAs by FISH and sense probe controls. (A) Trp53INP2 mRNA FISH antisense probe signal. (B) NET1 mRNA FISH antisense probe signal. (C–H) Sense probe controls for all probes shown in Figures 1 and S1. All samples were stained at the same time than their antisense counterpart, and imaged with identical light conditions. Scale bars: 25 µm. (TIF) [file pbio.1001439.s001.tif]

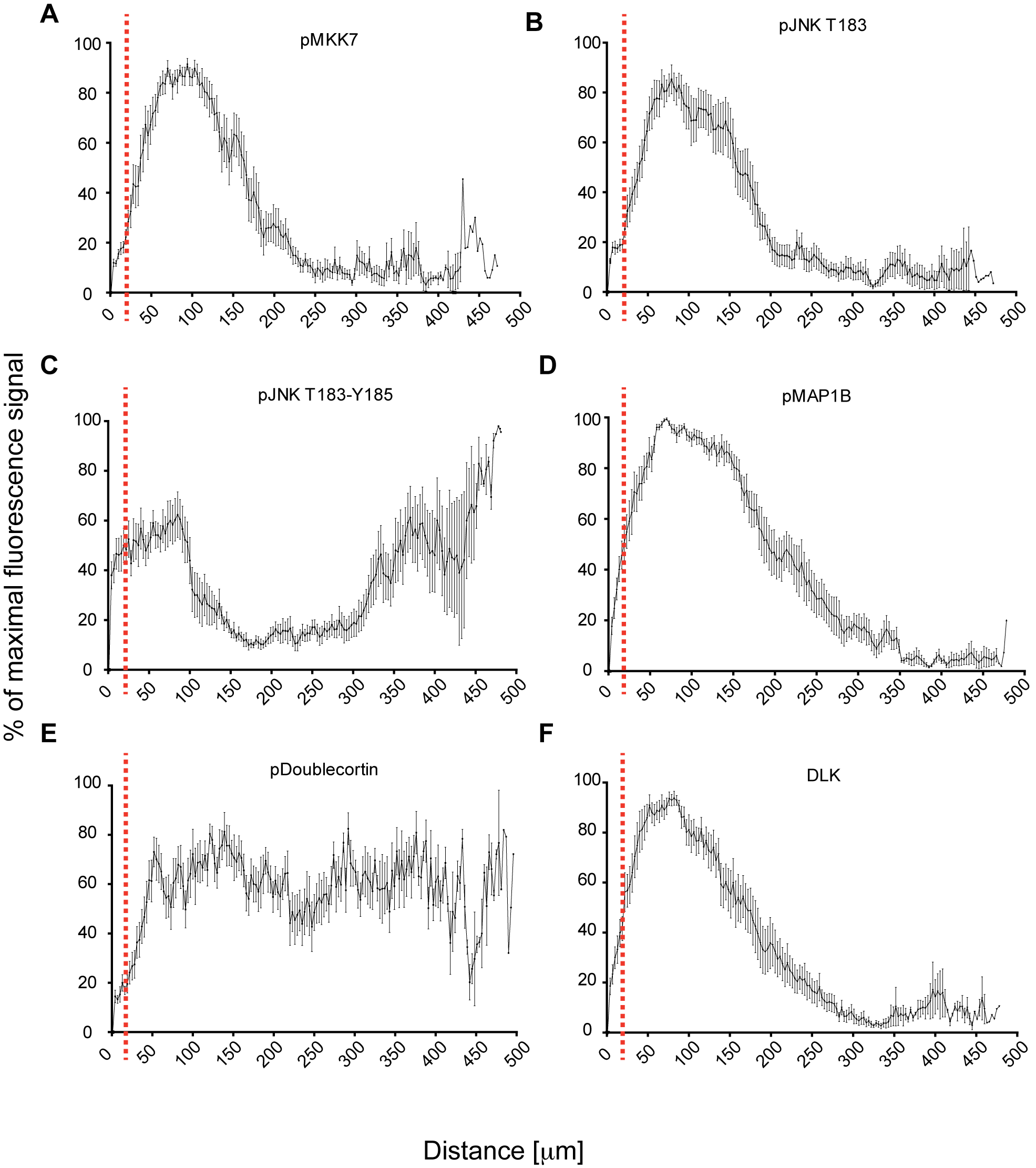

Supplement: Figure S2 — Quantification of the spatial patterns exhibited by phosphorylated forms of different JNK signaling network components (and of total DLK). Confocal fluorescent micrographs of differentiated N1E-115 cells immunostained for different components with similar neurite length were subjected to line scan analysis with a 500-µm long line. The mean ± SEM of normalized fluorescence intensity profiles from ten neurites are shown. The red vertical dotted line denotes the soma/neurite interface. (A) pMKK7. (B) pJNK T183. (C) pJNK T183 Y185. (D) pMAP1B. (E) pDoublecortin. (F) DLK. (TIF) [file pbio.1001439.s002.tif]

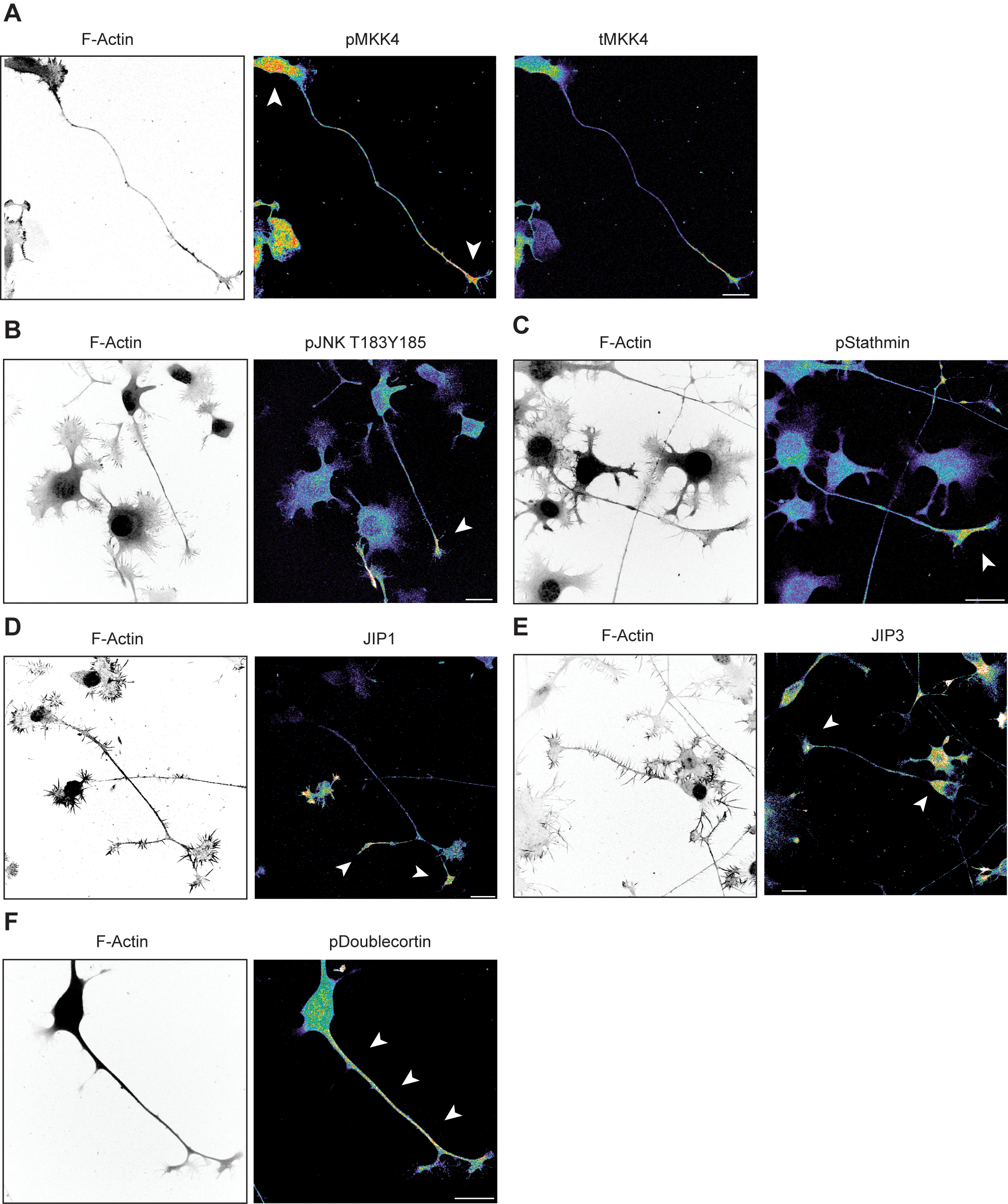

Supplement: Figure S3 — Subcellular localization of additional components of the neurite JNK signaling network. Representative confocal fluorescent micrographs of differentiated N1E-115 cells immunostained for different components are shown. Images are shown with color-coded fluorescence intensities (warm and cold colors represent high and low fluorescence intensities, respectively). F-actin (phalloidin staining) images are shown in ibw contrast. (A) pMKK4 and tMKK4. Note inverse distribution compared with pMKK7 with high signal intensity in the growth cone decreasing in the neurite (pointed to by arrowhead). Substantial pMKK4 signal is also found in the soma (pointed to by arrowhead). (B) pJNK T183Y185. Note high signal in the growth cone (pointed to by arrowhead), low signal in the neurite. (C) pStathmin. Note high signal in the growth cone (pointed to by arrowhead), low signal in the neurite. (D) JIP-1. Note high signal in the growth cone (pointed to by arrowhead), low signal in the neurite. (E) JIP-3. Note high signal in the growth cone and the soma (pointed to by arrowhead), low signal in the neurite (pointed to by arrowhead). (F) pDoublecortin. Note identical signal intensity in the soma and the neurite. The three arrowheads point to homogeneous signal intensity in the neurite. Scale bars: 25 µm. (TIF) [file pbio.1001439.s003.tif]

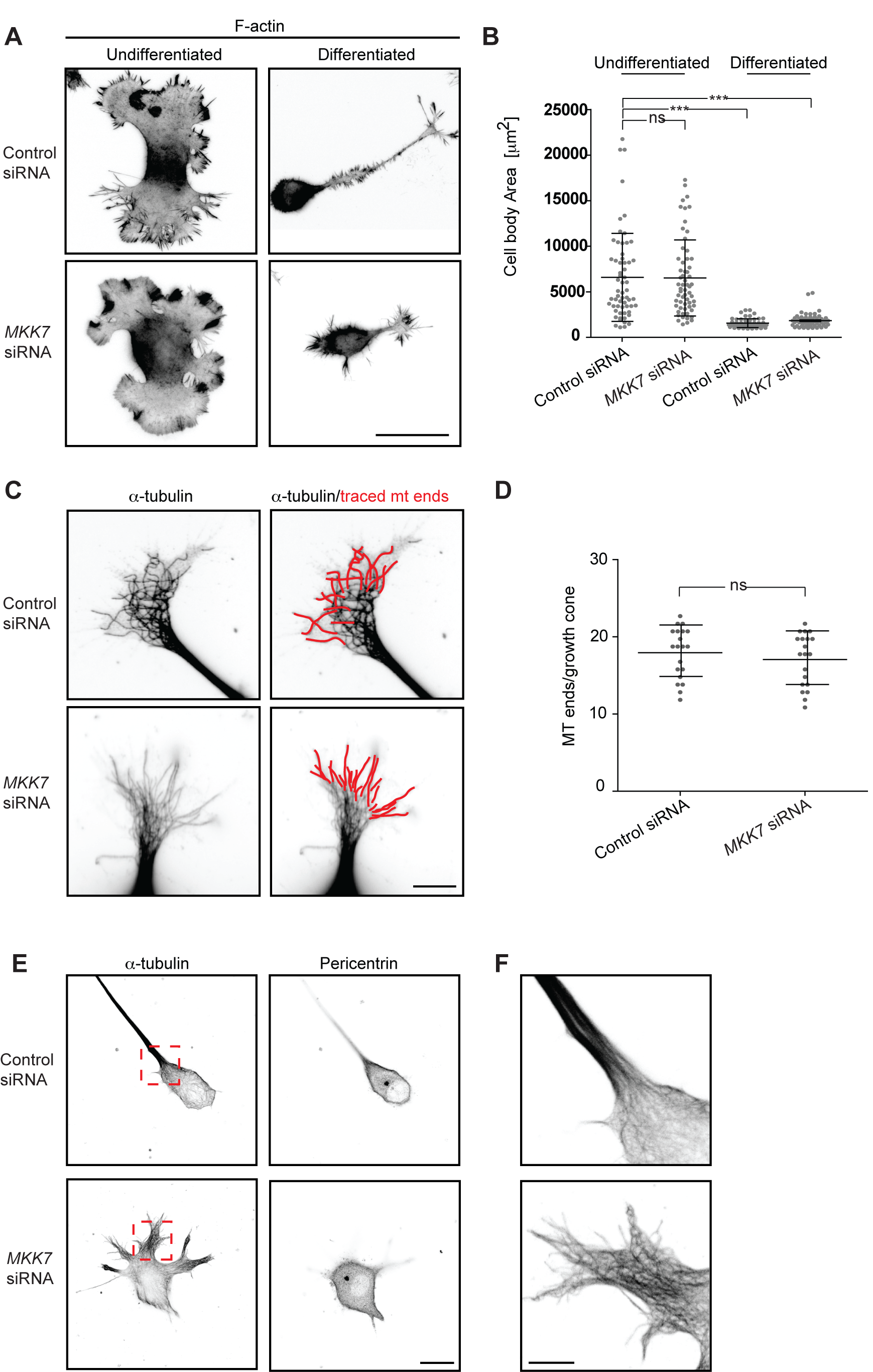

Supplement: Figure S4 — Further characterization of the effect of MKK7 KN on cell morphology and the cytoskeleton. (A) Effect of MKK7 KN on N1E-115 cell morphology in the undifferentiated and differentiated state. Undifferentiated and differentiated cells were plated on laminin-coated coverslips and allowed to adhere for 24 h (F-actin phalloidin staining of control and MKK7 KN cells are shown (ibw contrast)). Scale bar = 50 µm. (B) Cell surface measurements of (A). Mean ± SD is shown. n = 60 cells. (C) Epifluorescence micrographs of the mt cytoskeletons of growth cones of control and MKK7 KN cells. Adherent growth cones in the morphodynamic state of protrusion were chosen. Top panels: α-tubulin stained growth cones. Bottom panels: manual tracings of mt ends. (D) Quantification of number of mt ends in the growth cone as shown in (C). Mean ± SD is shown. n = 20 growth cones. (E) Confocal micrographs of the centrosome in control and MKK7 KN cells. Cells were immunostained for α-tubulin and pericentrin. ibw contrast is shown. Scale bar = 10 µm. Note the presence of one centrosome in both control and MKK7 KN cells. This was observed in all cells of n = 20 control and MKK7 KN cells. (F) Close-ups of pictures shown in (E) illustrating the unbundling phenotype. Scale bar = 10 µm. (TIF) [file pbio.1001439.s004.tif]

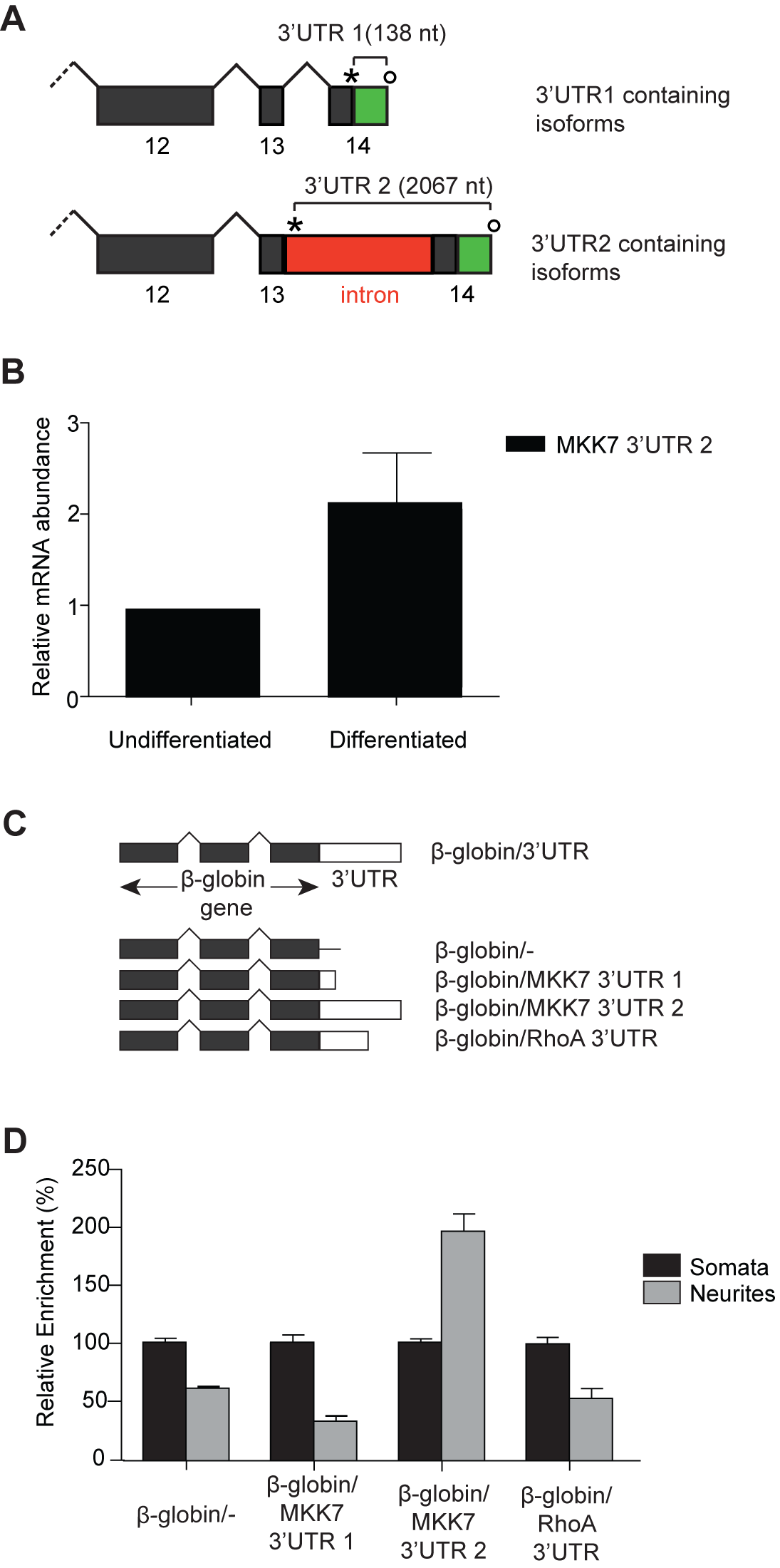

Supplement: Figure S5 — Identification of determinants that allow MKK7 mRNA localization. (A) Schematic of the 3′ end of MKK7 gene structure from the ENSEMBL database. Alternative splicing creates transcripts with either 3′-UTR1 (138 nt depicted in green) or 3′-UTR2 by retention of the intron between exons 13 and 14 (depicted in red). Thus, the 3′-UTR2 region also contains the 138-nucleotide stretch from 3′-UTR1. Termination codons (*) and polyadenylation sites (o) are indicated. (B) Relative expression levels of 3′-UTR2 containing mRNAs in non-differentiated and differentiated N1E-115 cells. RT-qPCR with 3′-UTR2 specific primers was performed on equal amounts of total mRNA from N1E-115 cells in the non-differentiated and differentiated state. n = 3 experiments. Mean ± SD is shown. p-value = 0.03. (C) Human β-globin chimeric constructs schematics. Black boxes, exons; black lines, introns; white boxes, 3′-UTR. (D) Subcellular localization of exogenously expressed constructs. The different constructs were transiently transfected in N1E-115 cells of which neurite and soma fractions were purified. Equal neurite and soma mRNA amounts were then probed by RT-qPCR to determine relative enrichment in each fraction using human β-globin-specific primers. Note that a 3′-UTR sequence of the RhoA mRNA, which our genome-wide assay was not identified to be neurite-enriched, did not lead to β-globin mRNA neurite enrichment. n = 3 experiments. Mean ± SD is shown. (TIF) [file pbio.1001439.s005.tif]

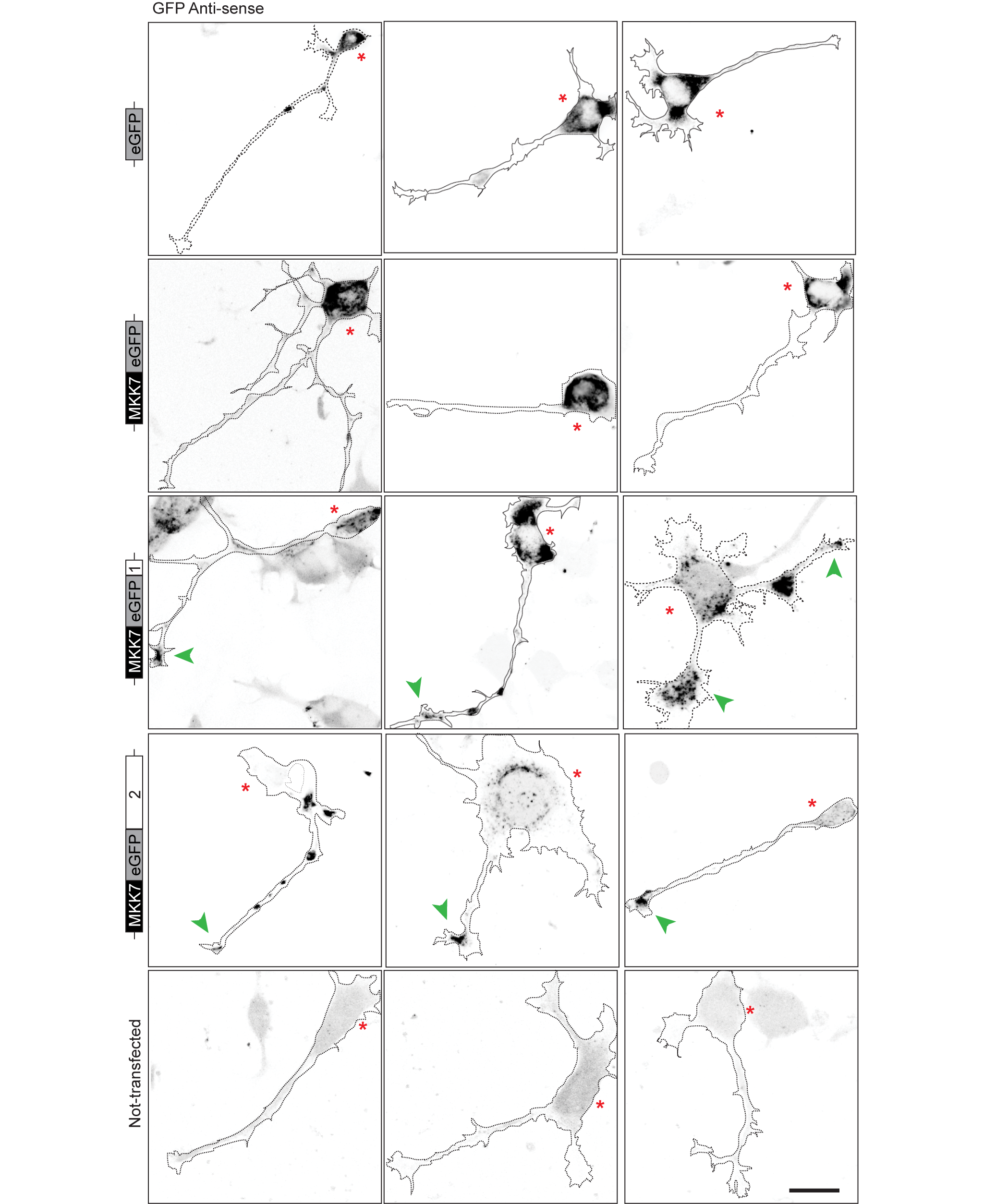

Supplement: Figure S6 — Representative FISH micrographs of exogenously expressed GFP , MKK7-GFP/- , MKK7-GFP/3′-UTR1 , and MKK7-GFP/3′-UTR2 mRNAs. Representative confocal fluorescence micrographs of GFP mRNA FISH in differentiated N1E-115 cells. Images from non-transfected or cells transfected with plasmids encoding GFP, MKK7-GFP/-, MKK7-GFP/3′-UTR1, and MKK7-GFP/3′-UTR2 are shown. Fluorescence intensity in all images are scaled identically and shown in ibw contrast. An outline of the cell generated using phalloidin staining in another channel is shown. Green arrows point to growth cone, red asterisks point to somata. Scale bar: 30 µm. (TIF) [file pbio.1001439.s006.tif]

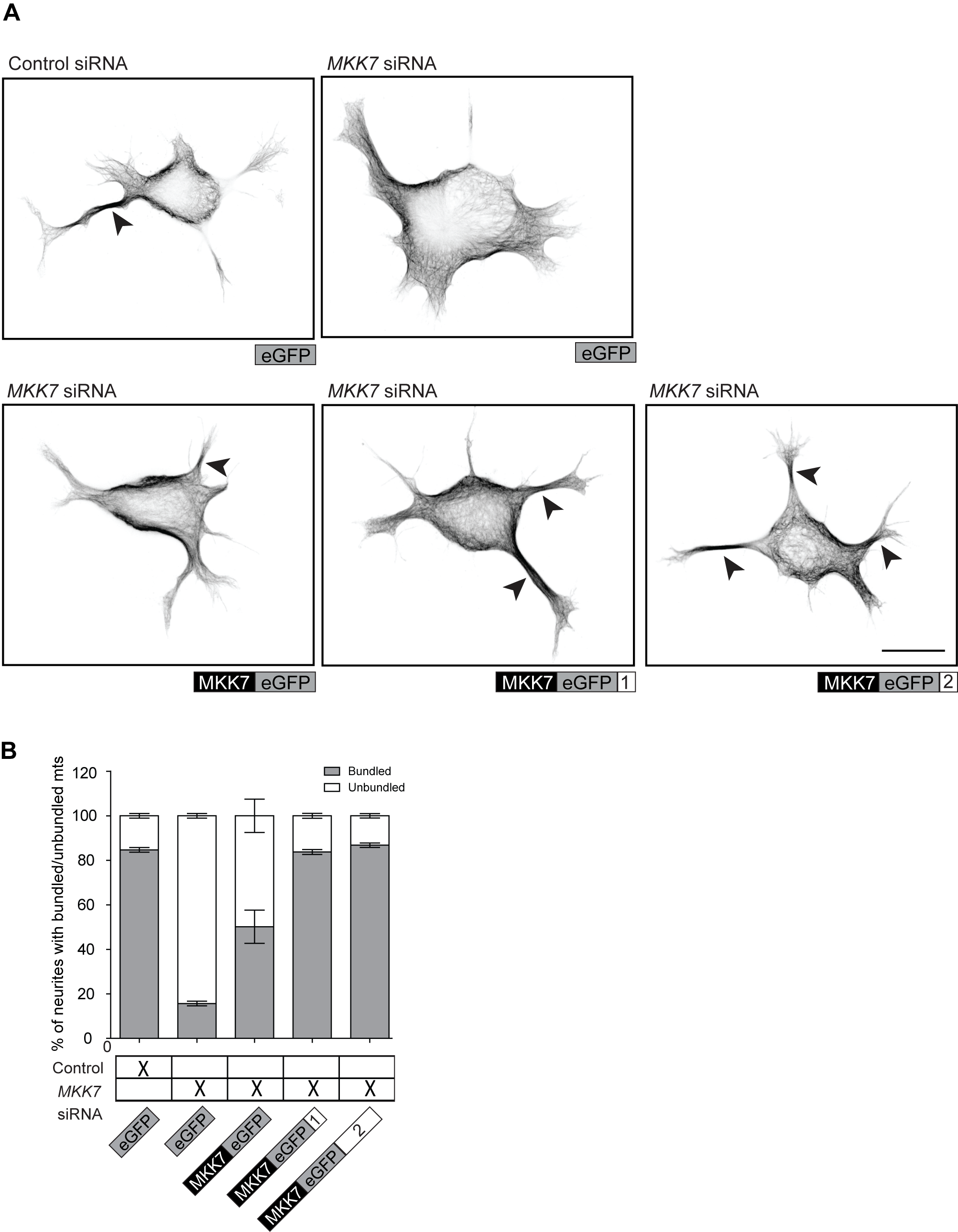

Supplement: Figure S7 — Additional MKK7 mRNA rescue experiments: evaluation of mt bundling. (A) Representative high resolution confocal fluorescence micrographs of α-tubulin stained N1E-115 cells of the MKK7 KN rescue experiment. Ibw contrast is shown. Cells were fixed at 8 h post-replating, allowing for short neurites in all experimental conditions, and fair comparison between wt, MKK7 KN, and rescued cells. (B) Quantification of images observed in (A). Percentage of neurites with bundled/unbundled mts are shown. SEM from two sets of quantifications of 30 cells are shown. Approximately, four neurites/cell were evaluated. (TIF) [file pbio.1001439.s007.tif]

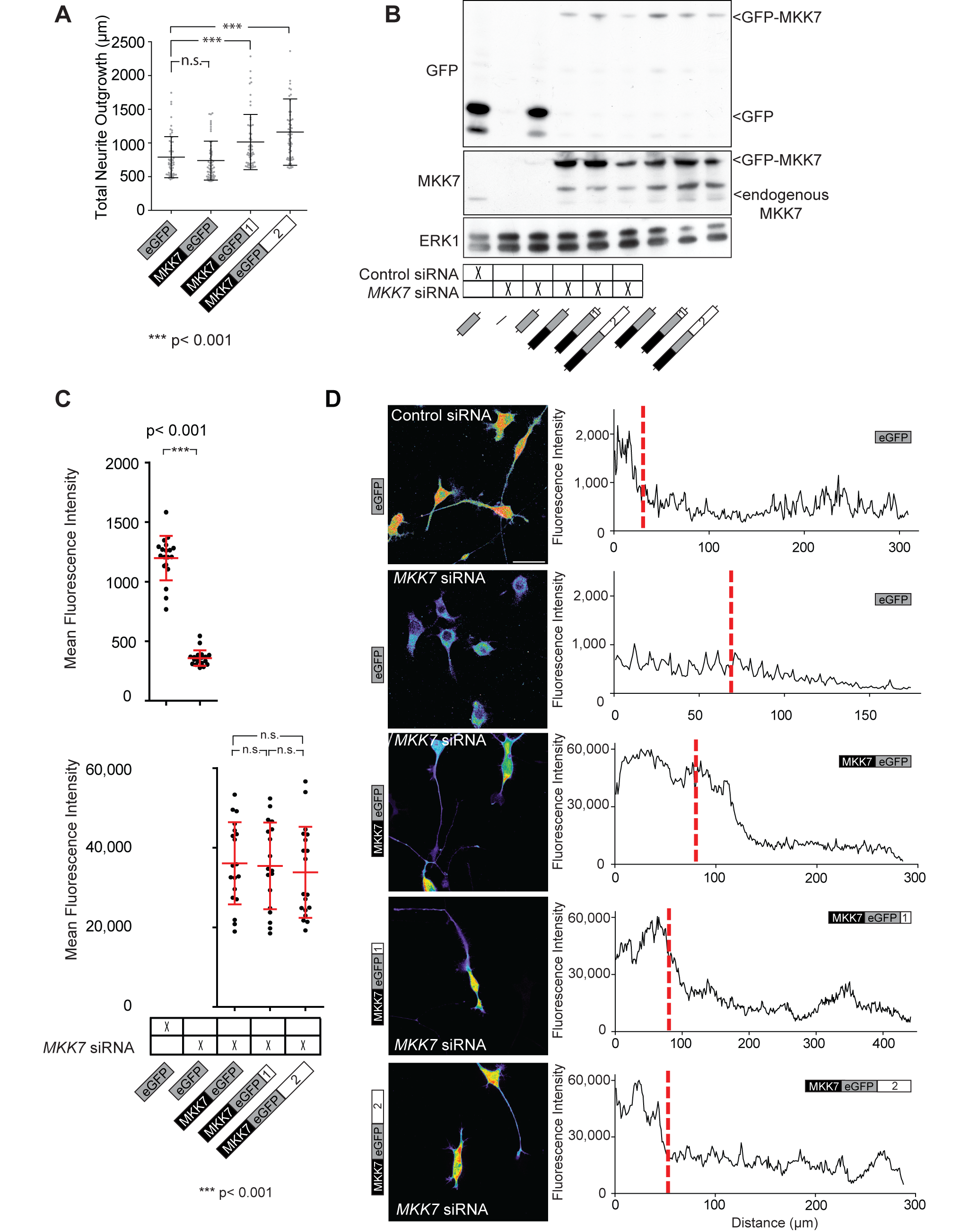

Supplement: Figure S8 — Additional MKK7 mRNA rescue experiments: neurite outgrowth measurements, quantification of tMKK7 expression levels and subcellular localization. (A) Neurite outgrowth measurements of differentiated N1E-115 cells overexpressing MKK7-GFP/-, MKK7-GFP/3′-UTR1, and MKK7-GFP/3′-UTR2 mRNAs. Measurements from 10% cells with longest neurites are shown, n = 600 cells. (B) Western blot analysis of expression of endogenous MKK7 (anti-MKK7 antibody) and exogenously expressed MKK7-GFP (anti-GFP) in experiments shown in Figures 5 and S7A. ERK1 serves as loading control. (C) Characterization of tMKK7 fluorescence intensities in control, MKK7 KN cells rescued with different MKK7 constructs. Absolute fluorescence intensities are shown. Mean ± SD is shown. Note that fluorescence signals are shown in two separate graphs that have been scaled differently so as to highlight relative fluorescence (expression) levels with the adequate dynamic range. (D) Representative tMKK7 micrographs of differentiated N1E-115 MKK7 KN cells rescued with different exogenously expressed MKK7-GFP constructs. Associated line scans are also shown. Images are color-coded so that warm and cold colors represent high and low pMKK7 signal. Cells were stained, imaged, and fluorescence intensities scaled with identical conditions within one experiment. Images were acquired with a confocal microscope with a maximally open pinhole for adequate signal quantification. Absolute fluorescence intensities for the line scans are shown. Note that y-axis is scaled differently for specific experiments. Red, vertical dotted line represents soma/neurite interface. Scale bar: 50 µm. (TIF) [file pbio.1001439.s008.tif]

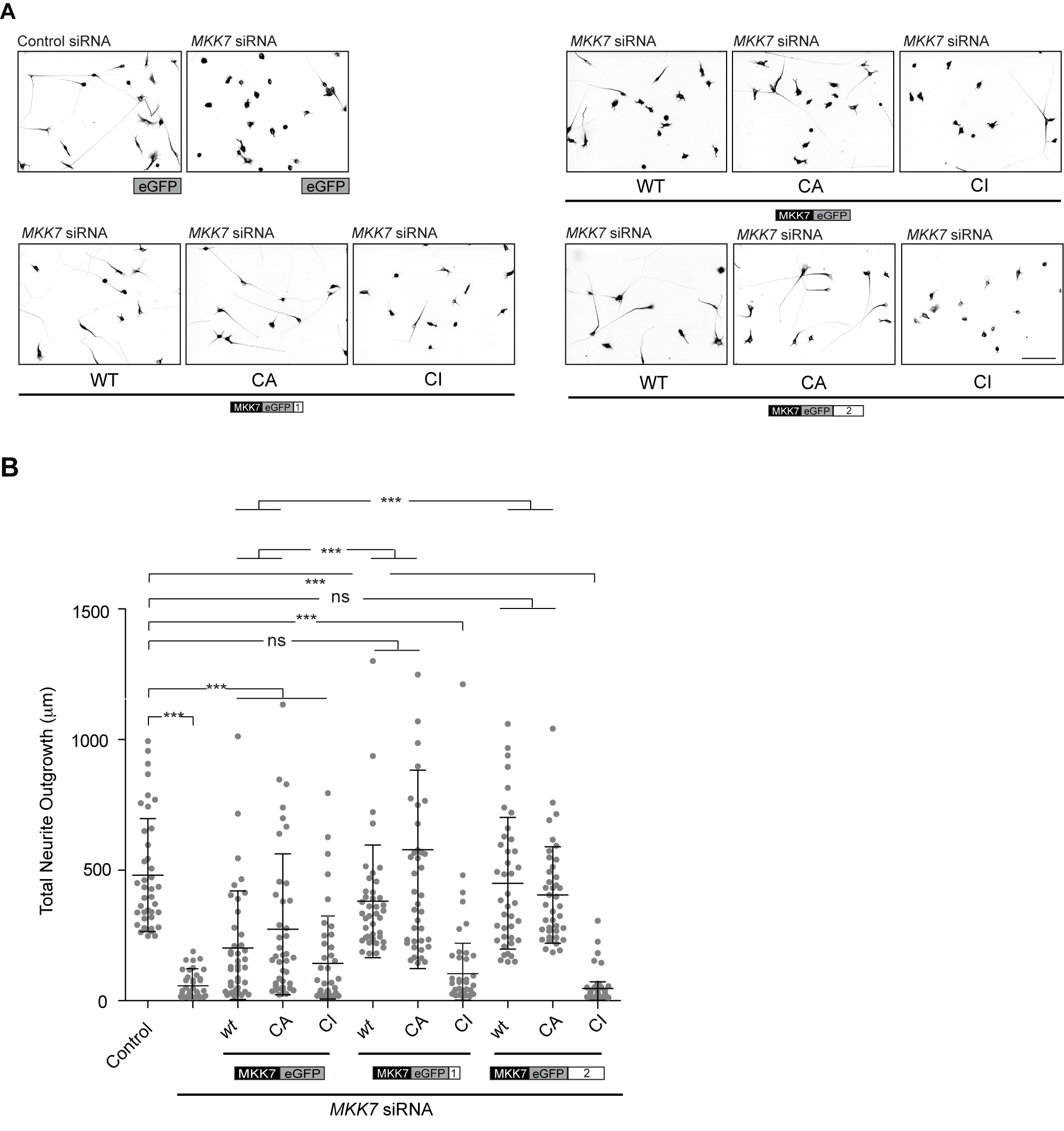

Supplement: Figure S9 — Additional MKK7 mRNA rescue experiments: rescue of MKK7 KN phenotype with CA and inactive forms of MKK7. (A) Representative micrographs of differentiated N1E-115 MKK7 KN cells rescued with different exogenously expressed wt, CA, and CI forms of MKK7-GFP/-, MKK7-GFP/3′-UTR1, and MKK7-GFP/3′-UTR2 mRNAs. Cells were immunostained for α-tubulin and are shown in ibw contrast. Scale bar: 100 µm. (B) Neurite outgrowth measurements from micrographs in (A) are shown. Measurements from 25% cells with longest neurites are shown, n = 100 cells. (TIF) [file pbio.1001439.s009.tif]

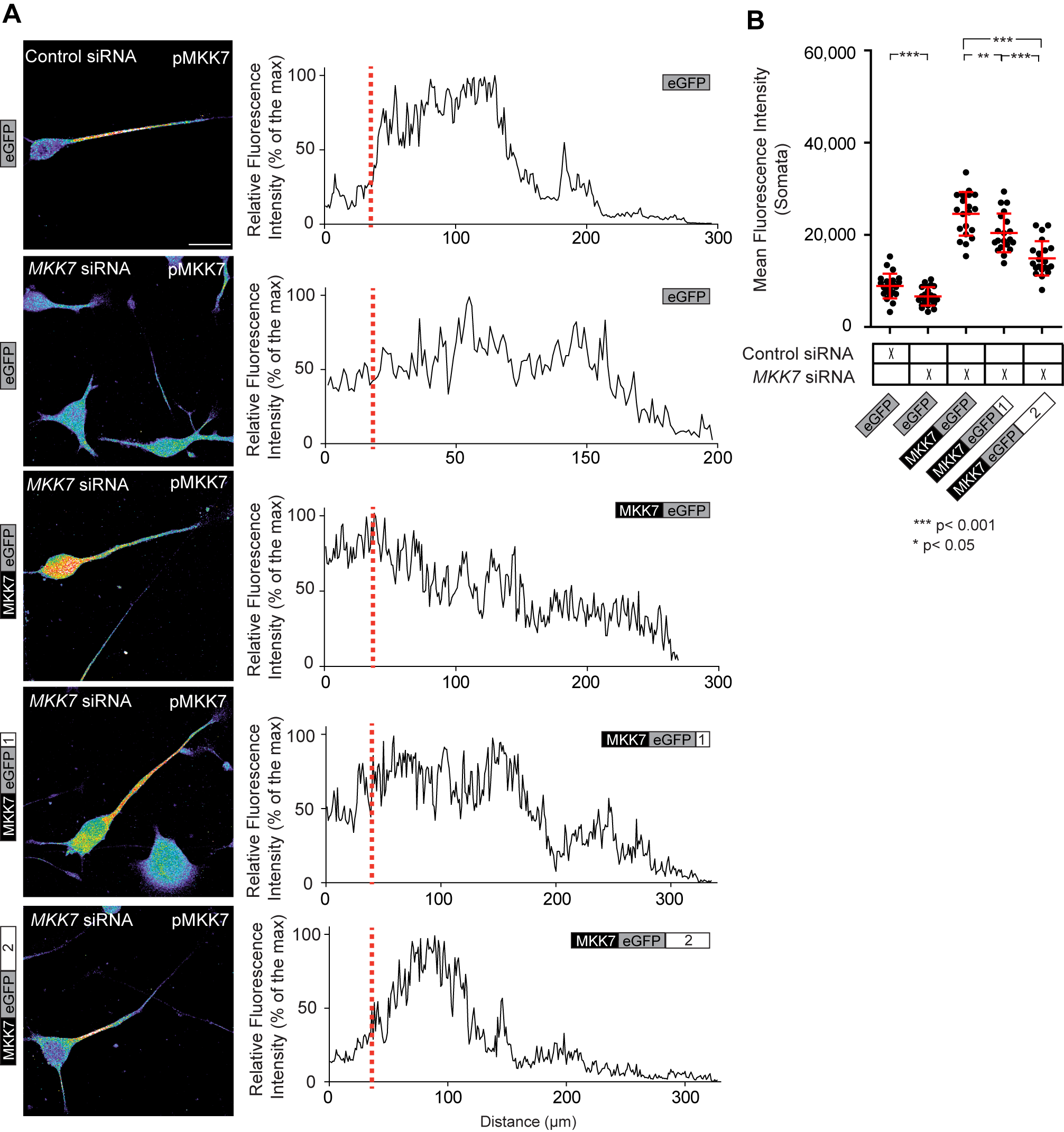

Supplement: Figure S10 — Additional MKK7 mRNA rescue experiments: quantification of pMKK7 subcellular patterns. (A) Representative pMKK7 micrographs of differentiated N1E-115 MKK7 KN cells rescued with different exogenously expressed MKK7-GFP constructs as shown in Figure 5D. Associated line scans are also shown. Absolute fluorescence intensities for the line scans are shown. Red, vertical dotted line represents soma/neurite interface. Scale bar: 25 µm. (B) Quantification of soma pMKK7 signals. Mean fluorescence intensity per soma are shown. Error bars represent SD, n = 20 cells. (TIF) [file pbio.1001439.s010.tif]

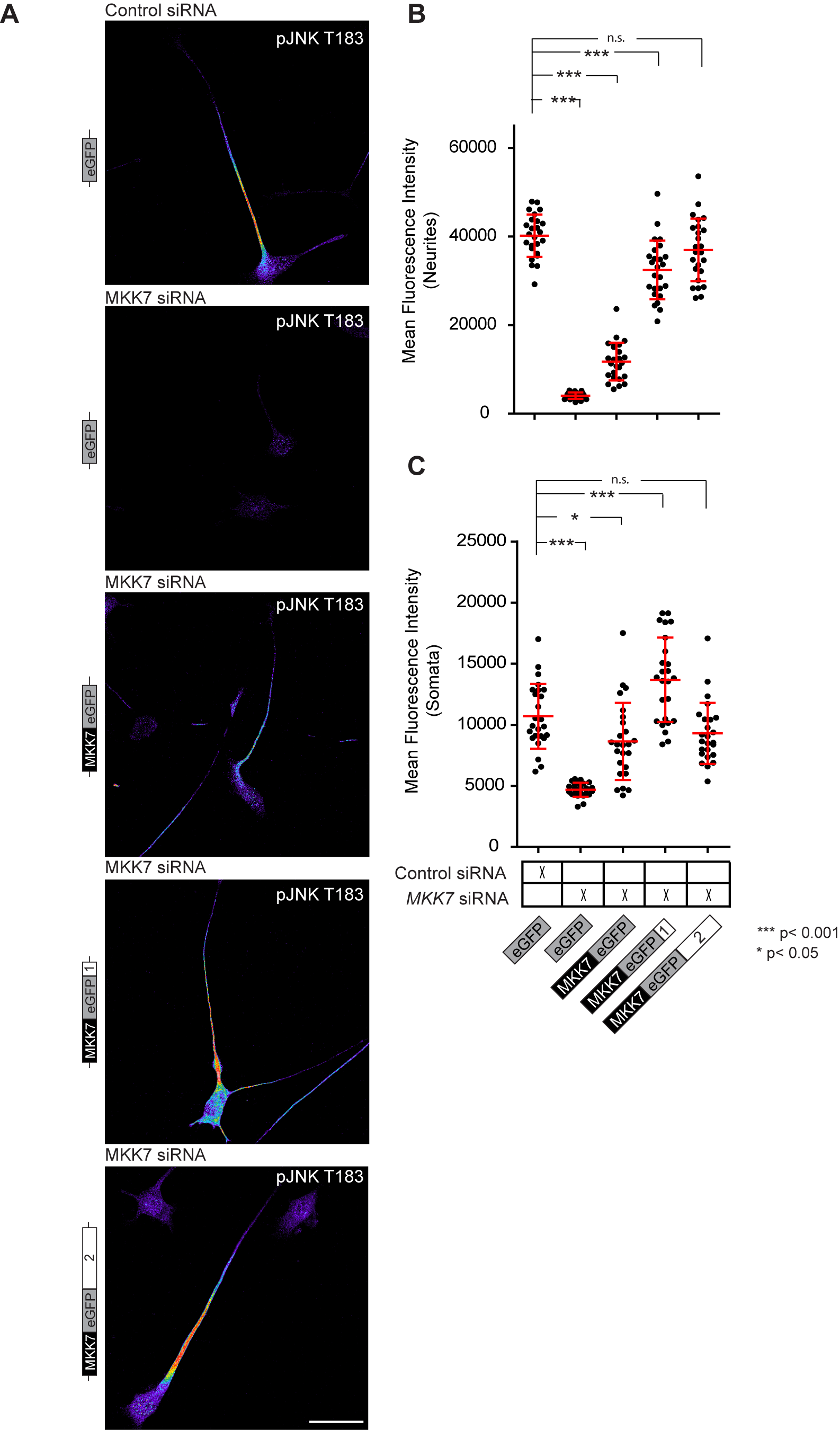

Supplement: Figure S11 — Additional MKK7 mRNA rescue experiments: effect on downstream pJNK T183 signals. (A) Representative micrographs of pJNK T183 signals of differentiated N1E-115 MKK7 KN cells rescued with different exogenously expressed MKK7-GFP constructs. Images are color-coded so that warm and cold colors represent high and low pMKK7 signal. Cells were stained, imaged, and fluorescence intensities scaled with identical conditions within one experiment. Images were acquired with a confocal microscope with a maximally open pinhole for adequate signal quantification. Scale bar: 25 µm. (B) Quantification of pJNK T183 signals in the neurite. Mean neurite fluorescence intensities per neurite are shown. Note robust neurite pMKK7 signal recovery with MKK7-GFP/3-UTR1 and 3′UTR2 constructs. Error bars represent SD, n = 20 cells. (C) Quantification of pJNK T183 signals in the soma. Mean soma fluorescence intensities per cell are shown. Error bars represent SD, n = 20 cells. (TIF) [file pbio.1001439.s011.tif]

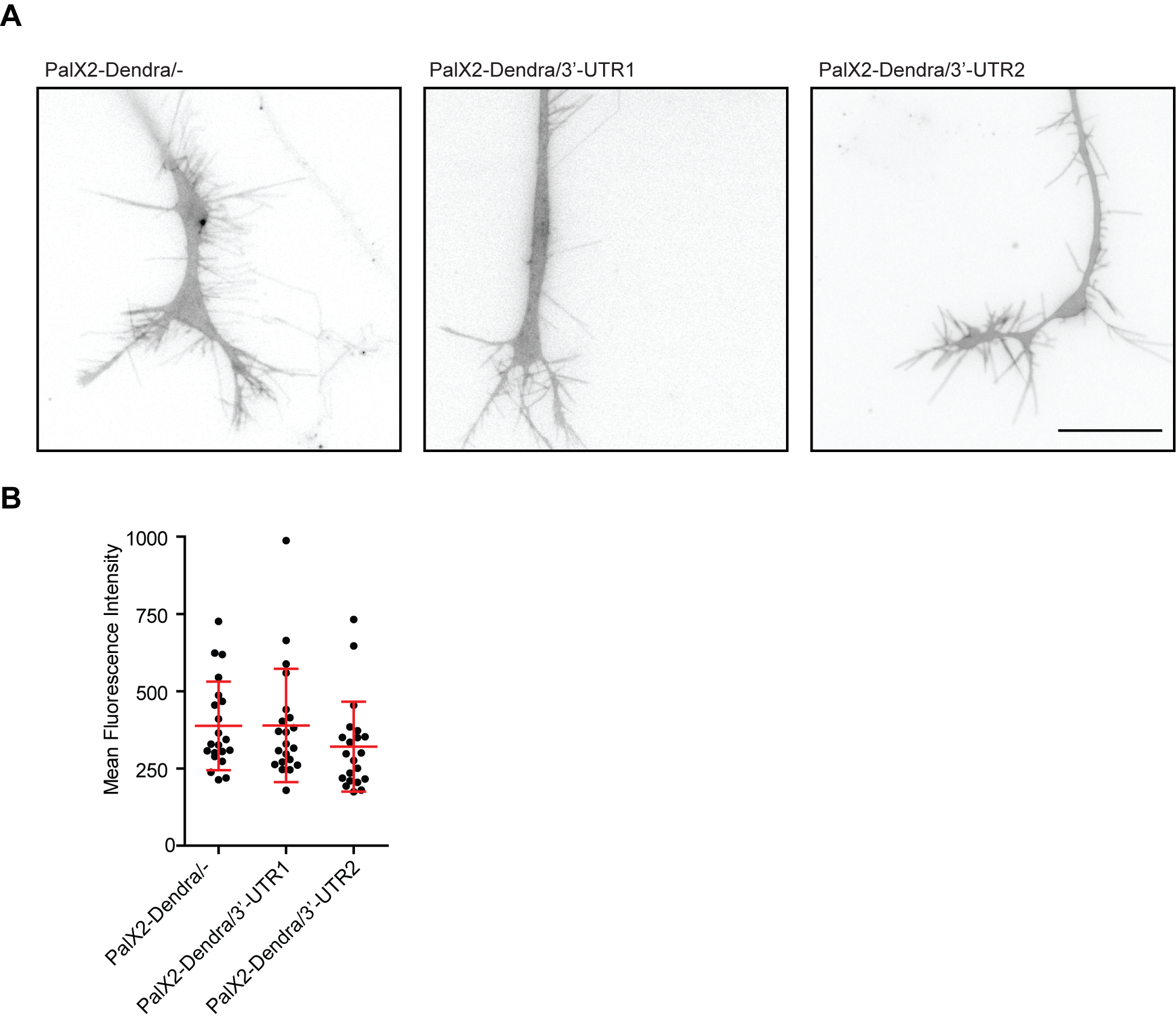

Supplement: Figure S12 — Additional controls for Palx2-Dendra2 reporter constructs. (A) Representative micrographs of PalX2-Dendra2/-, 3′-UTR1, 3′-UTR2 reporters in live growth cones without any bleaching procedure. Images are presented in ibw contrast. Scale bar: 5 µm. Note homogeneous distribution of fluorescence signal. (B) Quantification of PalX2-Dendra2/-, 3′-UTR1, 3′-UTR2 reporters expression levels. Average fluorescence intensities/cell are shown. Mean ± SD is shown. (TIF) [file pbio.1001439.s012.tif]

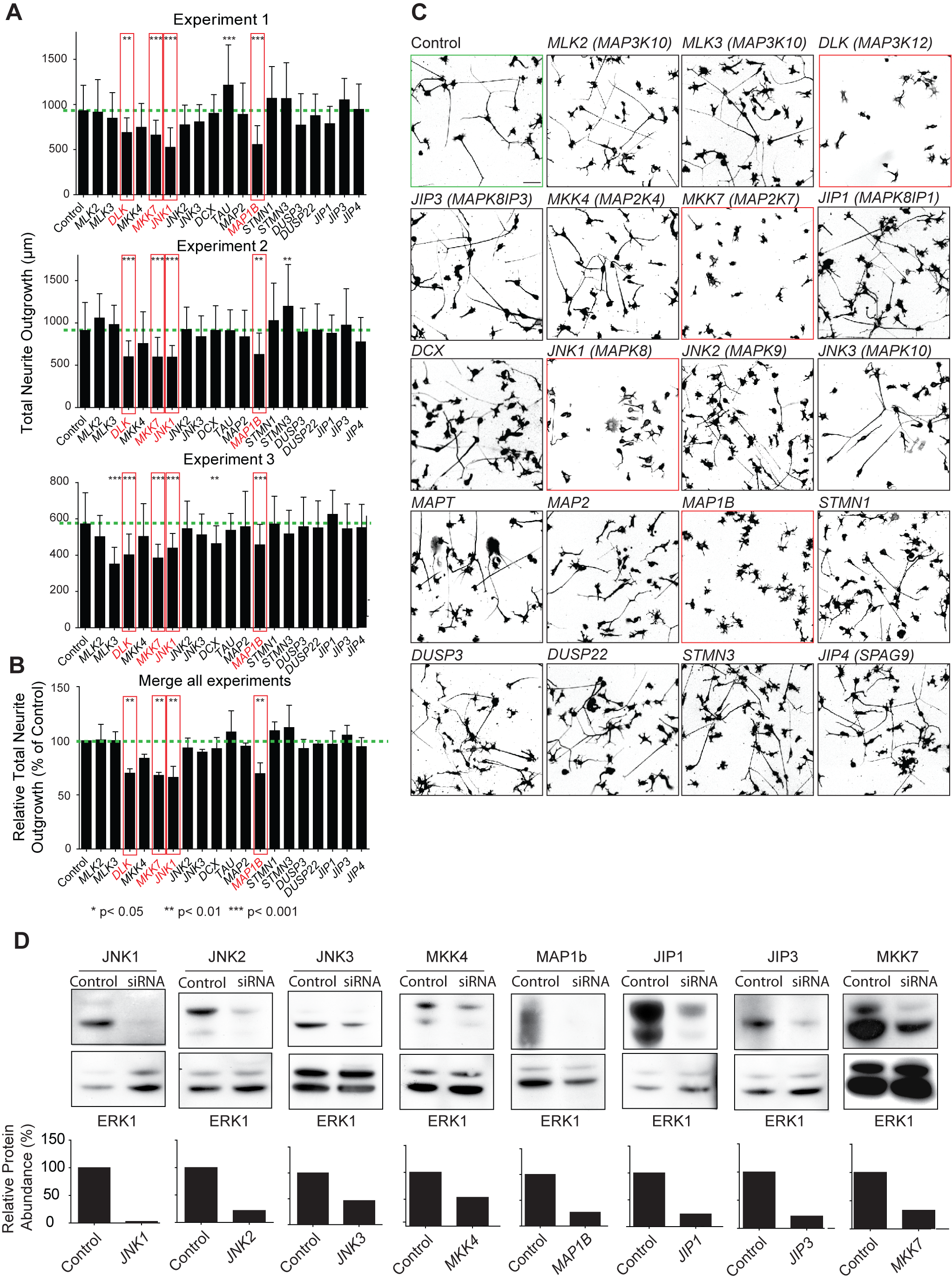

Supplement: Figure S13 — Neurite-localized JNK network siRNA screen. (A) Total neurite outgrowth measurements. Total neurite outgrowth length measurements of the 10% cells with longest neurites in response to KN of different gene products are shown. Results of three independent experiments are shown. Statistical significance is shown. In all the experiments mean ± SD is shown, n = 400 (experiment 1), n = 340 (experiment 2), n = 500 cells (experiment 3). (B) Averaged neurite measurements from the three experiments (A) expressed as percent change of control. Mean ± SD and statistical significance are shown. (C) Representative neurite outgrowth phenotypes. Representative micrographs of α-tubulin immunostained control of siRNA-transfected cells in ibw contrast. Example from one representative experiments is shown. Scale bar: 100 µm. (D) Assessment of KN efficiency. Western blot analysis of relative protein level in equal amounts of lysates of cells transfected with a non-targeting control or specific siRNA are shown. Quantification of KN efficiency normalized to ERK1 loading control are also shown. (TIF) [file pbio.1001439.s013.tif]

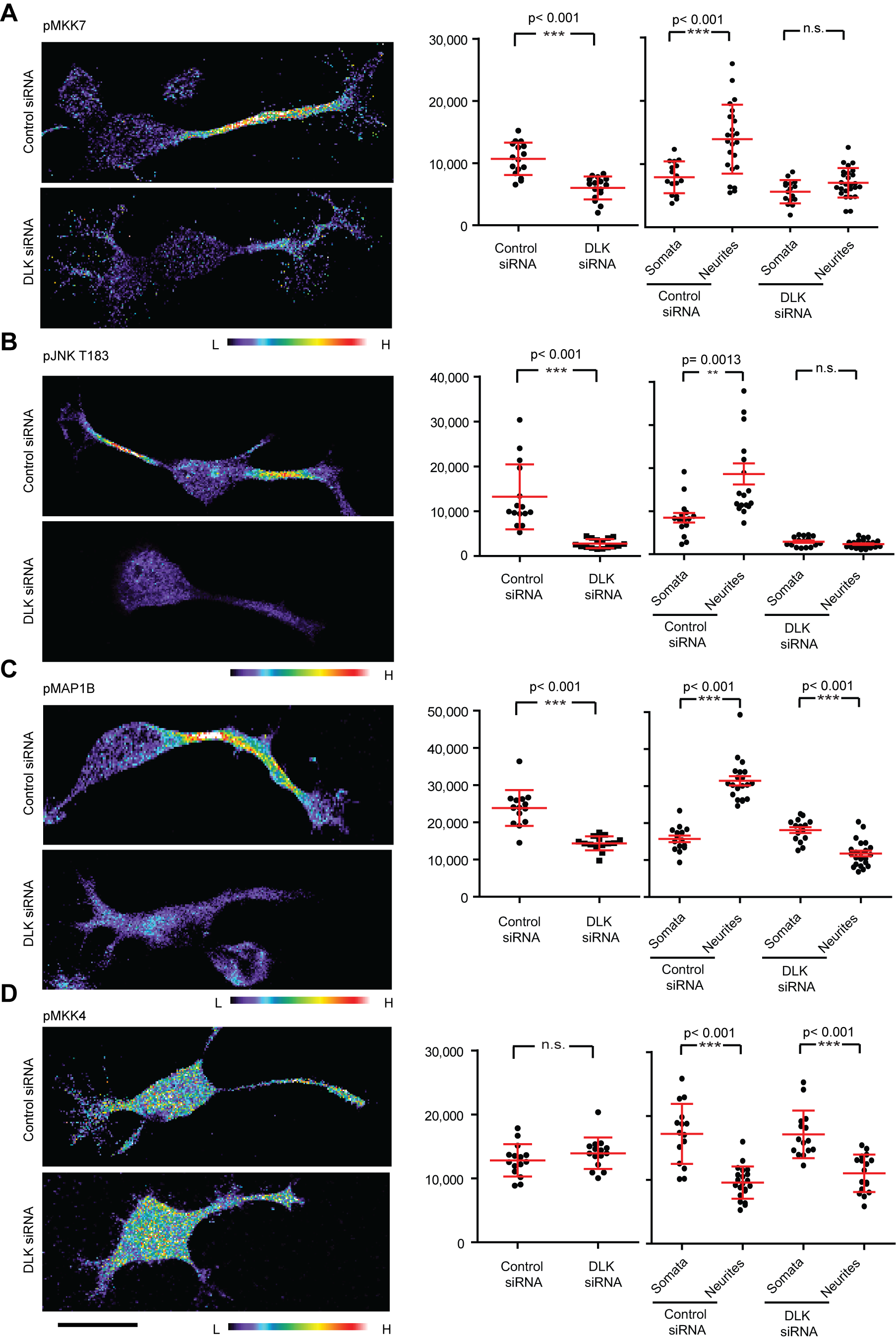

Supplement: Figure S14 — Hierarchy mapping of signaling components in the MKK7 signaling module: DLK KN. Control and DLK KN N1E-115 cells were allowed to differentiate on laminin-coated coverslips and were immunostained for pMKK7, pJNK T183, pMAP1b, or pMKK4. Images were then acquired with confocal microscope with identical exposure settings and with a maximally open pinhole for adequate comparison of fluorescence intensities. Only cells with short neurites were considered for a fair comparison. Left panels: representative images of control and KN cells are shown. Immunostain signal intensity is color-coded so that warm and cold colors represent high and low signals, respectively. Fluorescence micrographs have been scaled identically. Middle panels: average fluorescence intensities on a per cell basis. Right panels: somata and neurite average fluorescence intensities. Middle and right panels: y-axis represents fluorescence intensities. (A) pMKK7. (B) pJNK T183. (C) pMAP1b. (D) pMKK4. Note loss of phospho-signals in (A–C) consistent with the proposed hierarchy in the signaling module. Note that pMKK4 signal is not affected (D). Mean ± SD and statistical significance are shown. Scale bar: 25 µm. (TIF) [file pbio.1001439.s014.tif]

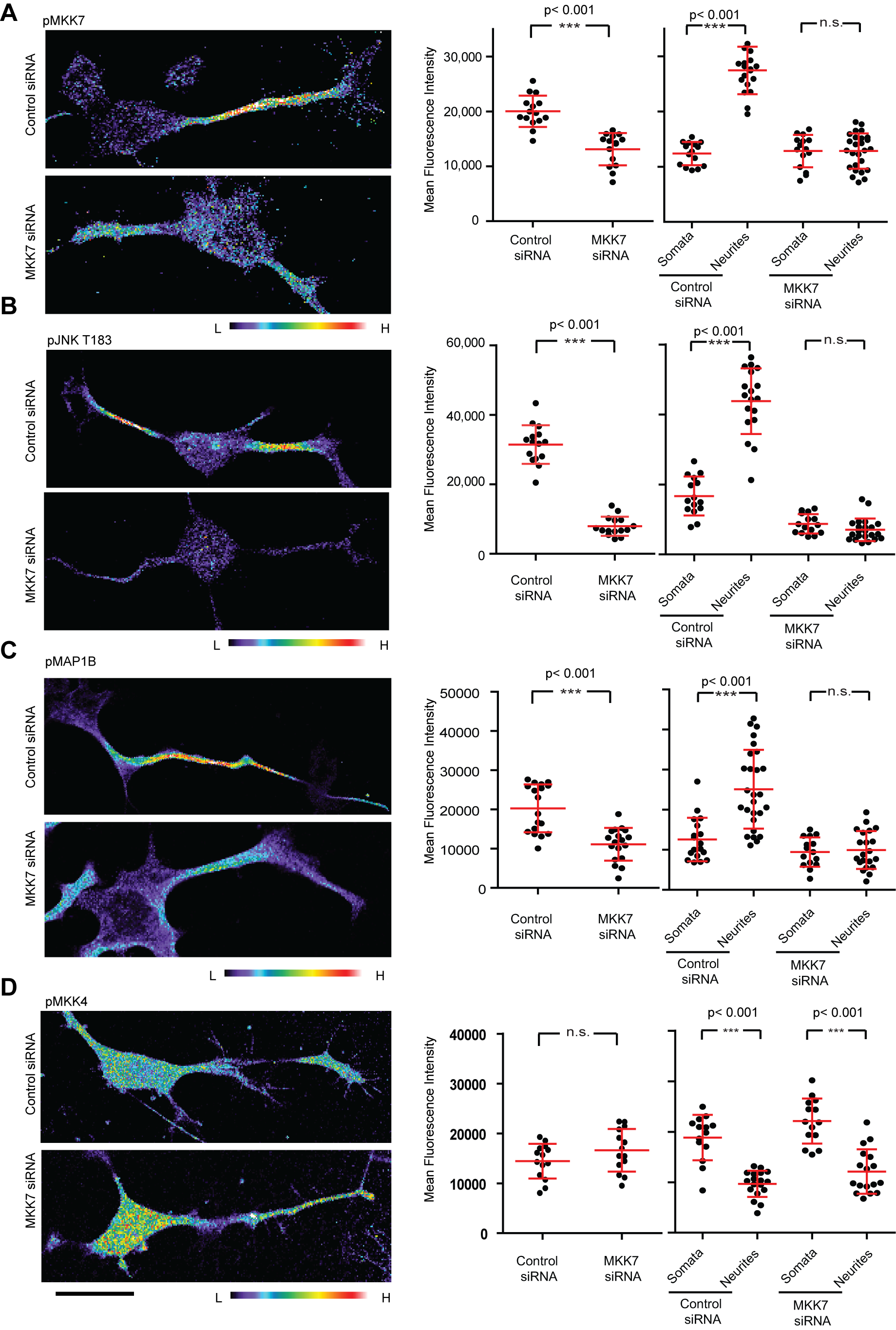

Supplement: Figure S15 — Hierarchy mapping of signaling components in the MKK7 signaling module: MKK7 KN. Experiments were performed as in Figure S14. (A) pMKK7. (B) pJNK T183. (C) pMAP1b. (D) pMKK4. Note loss of phospho-signals in (A–C) consistent with the proposed hierarchy in the signaling module. Note that pMKK4 signal is not affected (D). Mean ± SD and statistical significance are shown. Scale bar: 25 µm. (TIF) [file pbio.1001439.s015.tif]

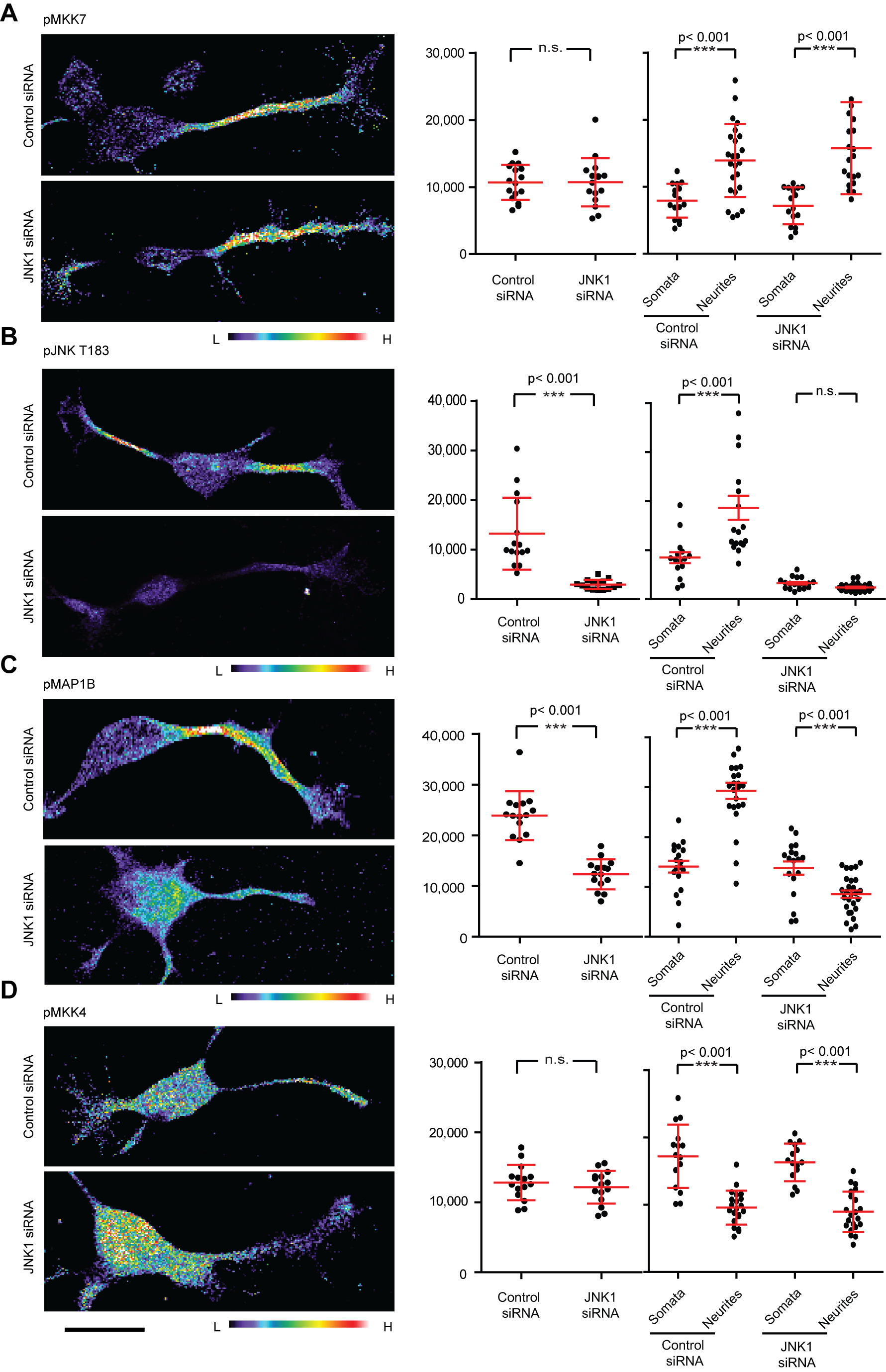

Supplement: Figure S16 — Hierarchy mapping of signaling components in the MKK7 signaling module: JNK1 KN. Experiments were performed as in Figure S14. (A) pMKK7. (B) pJNK T183. (C) pMAP1b. (D) pMKK4. Note absence of loss of pMKK7 in (A) and loss of phospho-signals in (B, C) consistent with the proposed hierarchy in the signaling module. However, pMKK4 (D) is not affected. Mean ± SD and statistical significance are shown. Scale bar: 25 µm. (TIF) [file pbio.1001439.s016.tif]

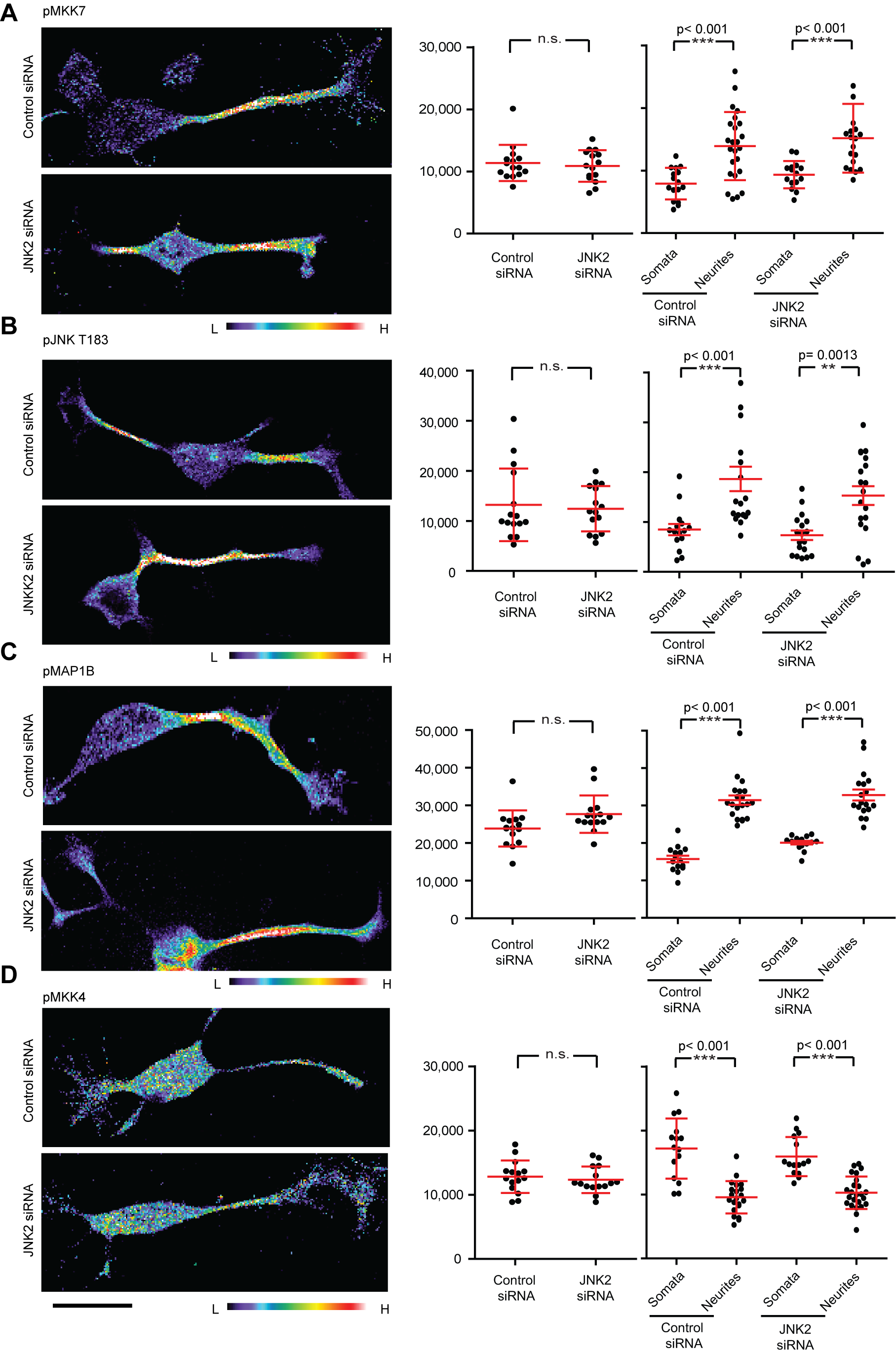

Supplement: Figure S17 — Hierarchy mapping of signaling components in the MKK7 signaling module: JNK2 KN. Experiments were performed as in Figure S14. (A) pMKK7. (B) pJNK T183. (C) pMAP1b. (D) pMKK4. Note absence of loss of phospho-signal in (A–D), consistently with no impact on the MKK7 signaling module. Mean ± SD and statistical significance are shown. Scale bar: 25 µm. (TIF) [file pbio.1001439.s017.tif]

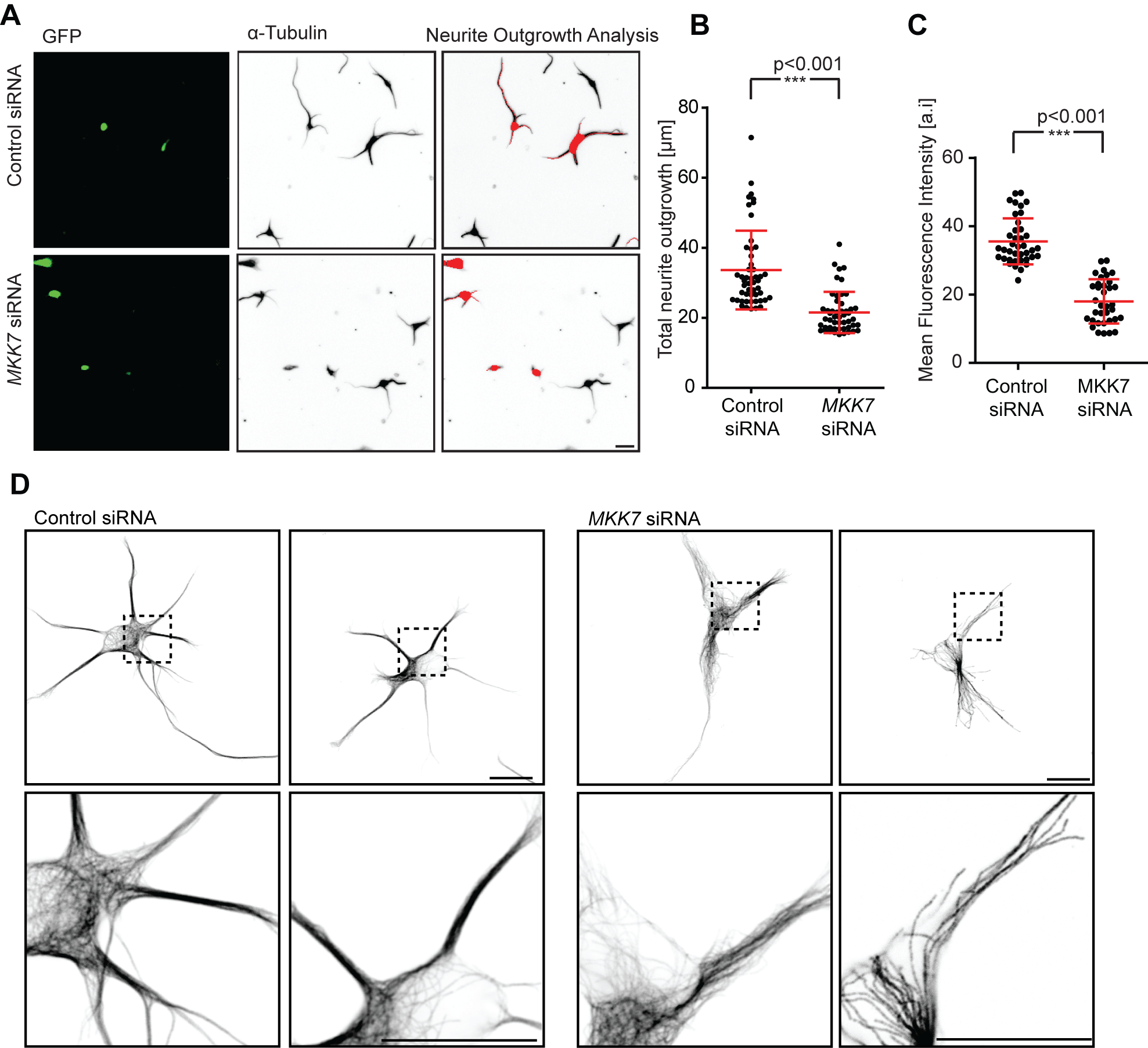

Supplement: Figure S18 — MKK7 KN in hippocampal neurons at DIV 1. (A) Representative fluorescent micrographs of control or MKK7 siRNA-transfected E18 embryonal hippocampal neurons. Neurons were fixed at DIV 1. Left panel: GFP marker fluorescence; middle panel: α-tubulin stain in ibw contrast; right panel: α-tubulin signal (black)/neurite segmentation (red). Scale bar: 10 µm. (B) Quantification of neurite outgrowth from n = 50 cells. (C) Quantification of tMKK7 signal in GFP-positive cells from n = 20 cells. (D) Additional examples of high resolution pictures of tubulin-stained control or MKK7 siRNA transfected hippocampal neurons. Scale bar: 10 µm. (TIF) [file pbio.1001439.s018.tif]

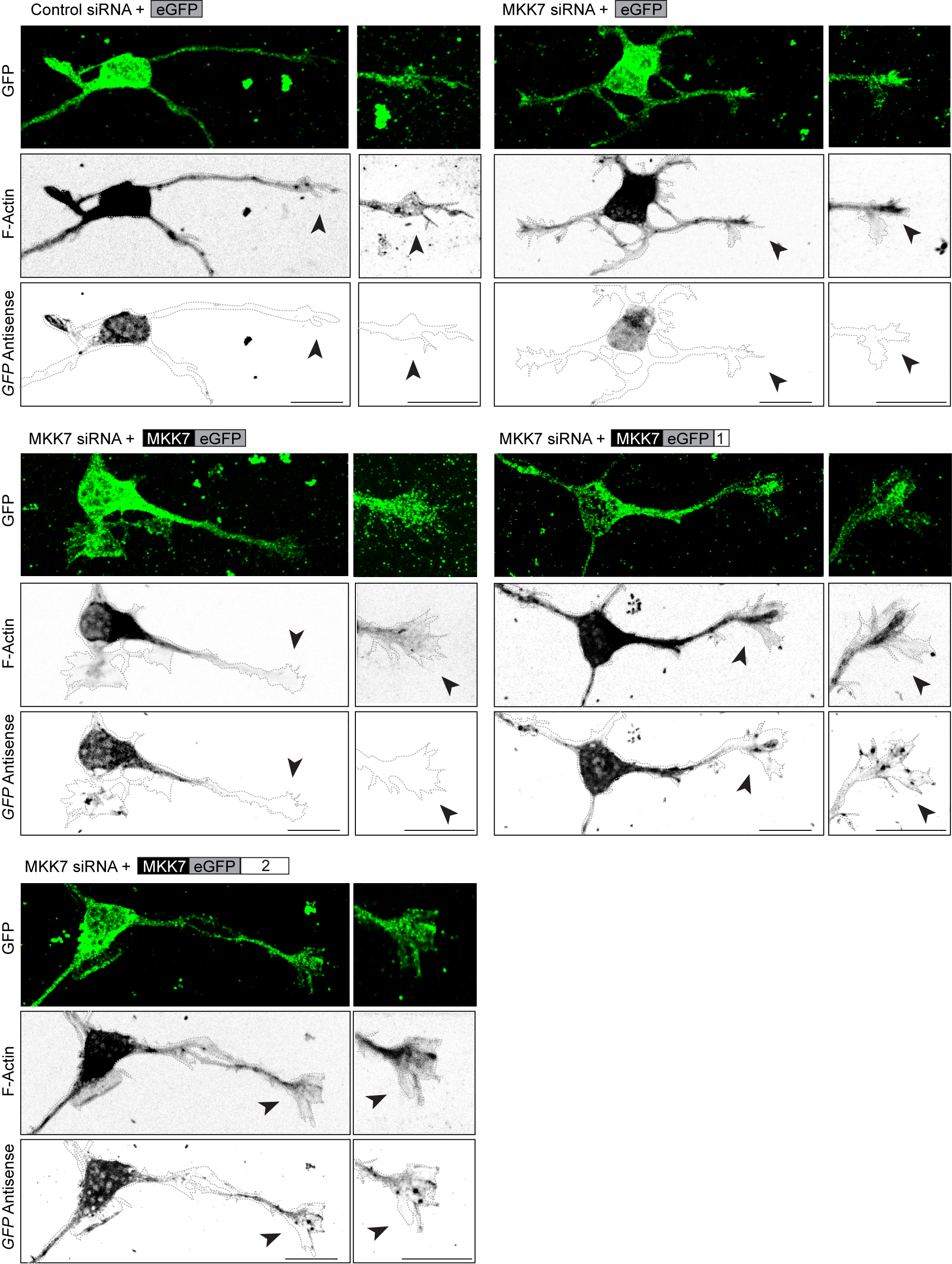

Supplement: Figure S19 — Representative FISH micrographs of exogenously expressed GFP , MKK7-GFP/- , MKK7-GFP/3′-UTR1 , and MKK7-GFP/3′-UTR2 mRNAs in primary hippocampal neurons. Representative confocal fluorescence micrographs of GFP mRNA FISH in primary hippocampal neurons. Images from cells expressing GFP, MKK7-GFP/-, MKK7-GFP/3′-UTR1, and MKK7-GFP/3′-UTR2 are shown. Cells were co-immunostained with an anti-GFP antibody to provide better signal to noise ratio to identify transfected cells. GFP signal is shown in green. F-actin and FISH signals are shown in ibw contrast and are scaled identically throughout the experiments. Black arrows point to growth cone. Scale bar: 10 µm, 5 µm (close-up). (TIF) [file pbio.1001439.s019.tif]

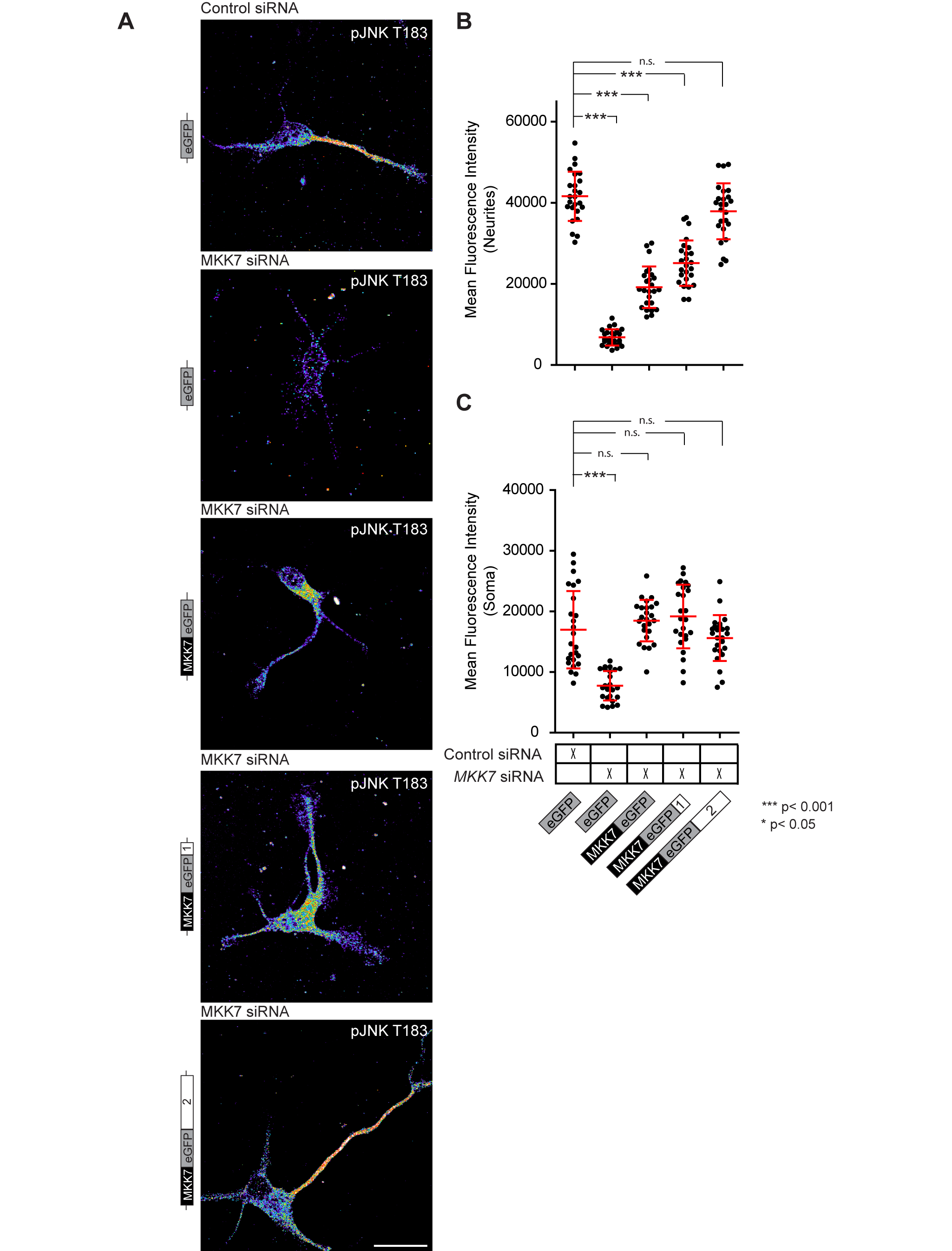

Supplement: Figure S20 — Subcellular localization of pJNK T183 in the MKK7 rescue experiment in primary hippocampal neurons. (A) Representative micrographs of pJNK T183 signals in the MKK7 rescue experiment. Images are color-coded so that warm and cold colors represent high and low pMKK7 or tMKK7 signal. Cells were stained with and anti pJNK T183 antibodies, imaged, and fluorescence intensities scaled with identical conditions within one experiment. Images were acquired with a confocal microscope with a maximally open pinhole for adequate signal quantification. Scale bars: 10 µm. (B) Quantification of pJNK T183 signals in the neurite. Mean neurite fluorescence intensities per neurite are shown. Only GFP-positive cells were considered. Note robust neurite pJNK T183 signal recovery with MKK7-GFP/3′-UTR2 constructs. n = 40 cells. SD is shown. (C) Quantification of pJNK T183 signals in the soma. Mean soma fluorescence intensities per cell are shown. Error bars represent SD, n = 40 cells. (TIF) [file pbio.1001439.s020.tif]
